# Long extrachromosomal circular DNA identification by fusing sequence-derived features of physicochemical properties and nucleotide distribution patterns

**DOI:** 10.1038/s41598-024-57457-5

**Published:** 2024-04-24

**Authors:** Ahtisham Fazeel Abbasi, Muhammad Nabeel Asim, Sheraz Ahmed, Andreas Dengel

**Affiliations:** 1 Department of Computer Science, Rhineland-Palatinate Technical University of Kaiserslautern-Landau, 67663 Kaiserslautern, Germany; 2 German Research Center for Artificial Intelligence GmbH, 67663 Kaiserslautern, Germany

**Keywords:** Computational biology and bioinformatics, Machine learning

## Abstract

Long extrachromosomal circular DNA (leccDNA) regulates several biological processes such as genomic instability, gene amplification, and oncogenesis. The identification of leccDNA holds significant importance to investigate its potential associations with cancer, autoimmune, cardiovascular, and neurological diseases. In addition, understanding these associations can provide valuable insights about disease mechanisms and potential therapeutic approaches. Conventionally, wet lab-based methods are utilized to identify leccDNA, which are hindered by the need for prior knowledge, and resource-intensive processes, potentially limiting their broader applicability. To empower the process of leccDNA identification across multiple species, the paper in hand presents the very first computational predictor. The proposed iLEC-DNA predictor makes use of SVM classifier along with sequence-derived nucleotide distribution patterns and physicochemical properties-based features. In addition, the study introduces a set of 12 benchmark leccDNA datasets related to three species, namely Homo sapiens (HM), Arabidopsis Thaliana (AT), and Saccharomyces cerevisiae (SC/YS). It performs large-scale experimentation across 12 benchmark datasets under different experimental settings using the proposed predictor, more than 140 baseline predictors, and 858 encoder ensembles. The proposed predictor outperforms baseline predictors and encoder ensembles across diverse leccDNA datasets by producing average performance values of 81.09%, 62.2% and 81.08% in terms of ACC, MCC and AUC-ROC across all the datasets. The source code of the proposed and baseline predictors is available at https://github.com/FAhtisham/Extrachrosmosomal-DNA-Prediction. To facilitate the scientific community, a web application for leccDNA identification is available at https://sds_genetic_analysis.opendfki.de/iLEC_DNA/.

## Introduction

Deoxyribonucleic acid (DNA) is comprised of billions of nucleotides and special arrangements of these nucleotides contain essential information for the development, functioning and inheritance of living organisms^1,2^. These nucleotides represent 25,000 protein-coding genes and various regulatory elements that control gene regulation^2^. In DNA sequence, these genetic components are organized in a structured manner and the sequence is wrapped around histone octamers also known as nucleosomes. Together around 30 million nucleosomes lead to the formation of chromosomes^3^. These chromosomes control vital biological processes like gene regulation, DNA replication, DNA damage response, and cell division^1,4^. However, aberrations within these processes produce additional genetic elements like extrachromosomal circular DNA (eccDNA)^5^.

To grasp the concept of eccDNA formation, one can examine the process of cell division^5,6^. During cell division, DNA replicates itself to ensure the transmission of chromosomes from parent to child cell. Within the replication process, DNA can incur damages, subsequently resulting in the fragmentation of chromosomes. DNA repair mechanisms reassemble these smaller segments and during the reassembling process, apart from chromosomes as a by-product eccDNAs are produced^5,6^. The lengths of these eccDNAs range from a few hundred to several thousand nucleotides because they are generated through random combinations of multiple segments^6^. Such fragments often harbor protein-coding genes, further complicating their impact on cellular processes such as gene expression, DNA replication, and DNA damage response

EccDNAs can be classified into two distinct categories based on their size and characteristics: short eccDNAs and long eccDNAs (leccDNA). EccDNAs with shorter lengths typically have tens to a few hundred nucleotides^6,7^. They are commonly found in the nucleus and cytoplasm of the cell and facilitate movement between genomic loci and drive genetic diversity along with adaptation. Moreover, they store genetic information and replicate independently as episomes. On the other hand, leccDNAs are longer and contain thousands of nucleotides^8^. LeccDNA are not formed only as the by-product of abnormal DNA replication process, but they can also form due to the recombination events in smaller eccDNA. Recent studies also provide similar evidence in agricultural weed systems^9,10^ and nuclear genomes^11^. LeccDNA are found only in the nucleus and contribute to genomic instability, gene amplification, cellular adaptation, and gene expression^6^. The presence of both types of eccDNA leads to excessive production of specific proteins, including oncogenes, enhancing the cell oncogenic potential and driving uncontrolled cell growth^12^. Futher, eccDNAs contribute to various diseases in multiple systems, such as glioblastoma, neuroblastoma, irregular immune response, and myocardial infarction^6,13–15^.

Identification of short eccDNA can reveal their roles in gene transfer, and genetic diversity. It is useful in understanding the molecular events of oncogene over-expression and therapeutic resistance. LeccDNA identification provides useful information about indications of genomic instability, genome organization as well as gene regulation. Furthermore, its identification is also useful for unveiling potential mechanisms responsible for the initiation and propagation of diseases such as cancer. Researchers are actively trying to explore its potential as a cancer biomarker and therapeutic resistance indicator^7,16^.

The identification of eccDNA is accomplished using a variety of wet-lab experimental methods, including pulsed-field gel electrophoresis (PFGE)^17^, southern blotting^18^, whole genome sequencing (WGS)^19^, fluorescence in situ hybridization (FISH)^20^, RT-PCR^18^, electron microscopy^21^, and rolling circle amplification (RCA)^22^. However, these methods often require prior knowledge or specific probes capable to bind with eccDNA which can limit their applicability to previously characterized eccDNAs. In addition, it is quite laborious, expensive, and time-consuming to identify eccDNAs at a larger scale across different organisms or cells.

The limitations of wet lab based methods and exceptional performance of AI based applications in natural language processing (NLP) tasks, have prompted a marathon of developing AI methods for DNA sequence analysis. Several AI models have been developed for various DNA analysis tasks such as enhancer identification^23,24^, DNA modification prediction^25,26^, promoter prediction^27^, DNA cyclizability prediction^28^, nucleosome position detection^29^ and so on. On the other hand, the identification of eccDNA is still being performed through wet lab-based methods due to the deficiency of AI applications for this particular task. According to the best of our knowledge, one predictor named DeepCircle^30^ is developed for the identification of short eccDNA sequences. There is currently no single predictor available for the identification of leccDNA, and also DeepCircle is not suitable for this specific purpose. The primary obstacle in utilizing DeepCircle for leccDNA identification lies in its reliance on the BERT model^30^, which can only handle sequence lengths of up to 512 tokens and leccDNA sequences exceed this token limit^31^.

In order to expedite and enhance research pertaining to the identification of leccDNA, there is an urgent necessity of a robust computational predictor. With an aim to develop a robust and precise computational predictor for leccDNA identification, the contributions of this study are manifold. Following the need for leccDNA identification datasets, it presents 12 benchmark datasets related to leccDNA sequences belonging to 3 different species i.e., *Homo sapiens* (HM), *Arabidopsis thaliana* (AT), and *Saccharomyces cerevisiae* (SC). It presents a robust and precise iLEC-DNA predictor that reaps the benefits of 2 different sequence encoding methods for transforming raw sequences into statistical vectors. Furthermore, to discriminate leccDNA and non-leccDNA sequences, it employs support vector machine (SVM) classifier that extracts more useful discriminative features from statistical vectors having nucleotide distribution patterns and physicochemical properties based information. Furthermore, it compares the performance of proposed predictor with more than 140 baseline predictors and 858 encoder ensembles that are developed by using 13 most widely used sequence encoding methods and 11 machine learning (ML) classifiers. It conducts extensive experimentation over 3 different species datasets to find important answers of  the following research questions; I) Do leccDNA sequences exhibit any distinctive nucleotide patterns that distinguish them from non-leccDNA sequences? II) How can variable-length leccDNA sequences be effectively handled to train ML classifiers? III) Which sequence encoding method is more competent in transforming raw leccDNA sequences into statistical vectors by incorporating discriminatory information? IV) Which sequence encoding method demonstrates better performance with which ML classifier? V) Which specific ensemble of sequence encoding methods  provide better classification performance? VI) Does the combined potential of the multiple sequence encoding methods enhance the classification efficacy? We believe answers to these questions will provide valuable guidance to the research community when it comes to selecting the optimal combination of encoding methods and classifiers. This will significantly contribute to the creation of an efficient end-to-end predictive pipeline.

## Results

### Key idea

Over the newly developed 12 benchmark leccDNA datasets, we generate an effective statistical representation based on the gap-kmer distribution and physicochemical properties based information using two sequence encoding methods namely, complementary k-spaced nucleic acid pairs (CKSNAP), and pseudo electron-ion interaction pseudopotentials of trinucleotides (PseEIIP). Using discriminatory features from CKSNAP and PseEIIP we develop a novel predictor based on SVM for leccDNA identification namely, iLEC-DNA. In order to prepare fixed-length leccDNA and non-leccDNA sequences without losing information-rich regions, we perform a thorough intrinsic 2-mer distribution analysis. To validate the observations from the intrinsic analyses, an extrinsic performance analysis is conducted which affirms the information-rich regions that play a critical role in leccDNA identification. In addition, we compared the performance of the proposed predictor with more than 140 baseline 857 advanced predictive pipelines developed based on 13 commonly used sequence encoding methods and 11 ML classifiers. Extensive experimentation shows that the proposed predictor is able to achieve suitable performance across diverse benchmark datasets for leccDNA identification.

### Summary of results

This section provides a comprehensive overview of the research objectives pertaining to the prediction of leccDNA. First, it investigates whether leccDNA sequences exhibit nucleotides distinctive patterns that can differentiate them from non-leccDNA sequences. It illustrates and compares the performance values of 143 baseline and 857 advanced predictors across 12 benchmark LeccDNA datasets. Finally, it illustrates the performance values of the proposed leccDNA predictor on 12 benchmark datasets.

### RQ I: nucleotide patterns in LeccDNA and non-leccDNA sequences

**Figure 1:**
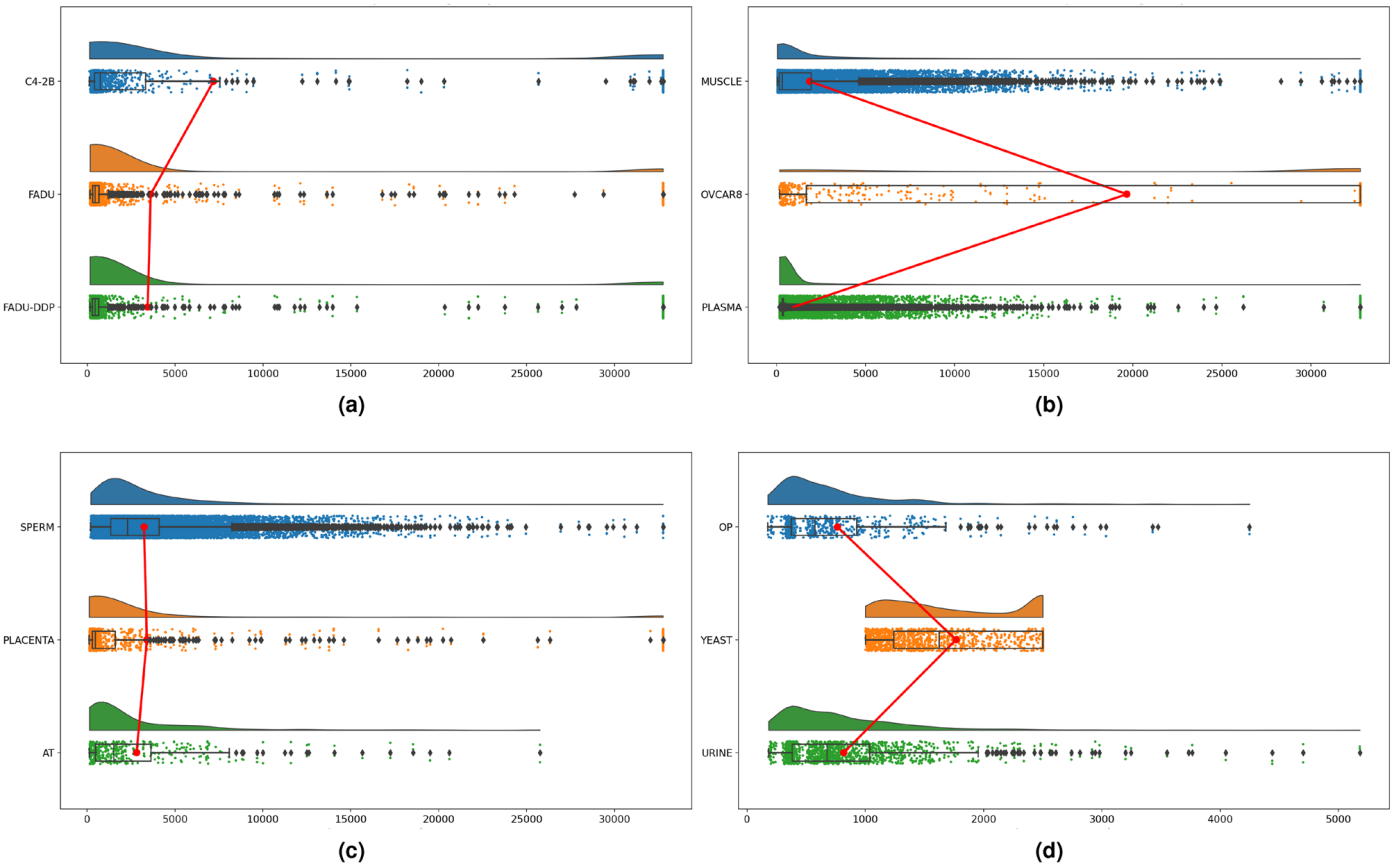
The distribution of sequence lengths across all benchmark datasets. X-axis represents the length of leccDNA and non-leccDNA sequences and y-axis represents the distribution of leccDNA and non-leccDNA sequences. The red line represents the median sequence lengths across a dataset.

In order to perform DNA sequence classification, ML predictors require uniform length of DNA sequences and distinct nucleotide patterns across various classes. As depicted in Fig. 1, there is considerable variability in the lengths of both leccDNA and non-leccDNA sequences. However, for the purpose of training ML classifiers, these sequences must be of a fixed length. To tackle this issue, one solution involves the direct addition of a padding character ’P’ within sequences. However, the substantial variations in leccDNA sequence lengths, spanning from five to thirty thousand nucleotides necessitate more padding values which introduce noise and bias in data. This influx of padding values not only disrupts the original data distribution but also undermines the model’s potential to generalize effectively on unseen data. In an alternate strategy^32^, first information-rich regions are explored in the sequences, and padding values are added after truncating all sequences with a specific length threshold. This approach produces fixed-length DNA sequences without introducing substantial bias in data.

With the objective of delineating information-rich regions and obtaining uniform-length DNA sequences, an initial step involves the segmentation of DNA sequences into discrete subsequences. Further, to gain insights into the density and patterns of distinct nucleotide pairs, in 4 different steps an intrinsic analysis is performed. First, the occurrences of 16 unique 2-mers within each subsequence are calculated in leccDNA and non-leccDNA subsequences. Next, The subsequence-based densities of 2-mers are computed separately for leccDNA and non-leccDNA sequences across 10 different sequence lengths. In the subsequent step, the density-based values are normalized with a total number of sequences. Finally, the density differences of 2-mers among leccDNA and non-leccDNA sequences are computed to reveal distinctive nucleotide patterns. Details related to information-rich region analyses are provided in Supplementary Sect. S1.

**Figure 2:**
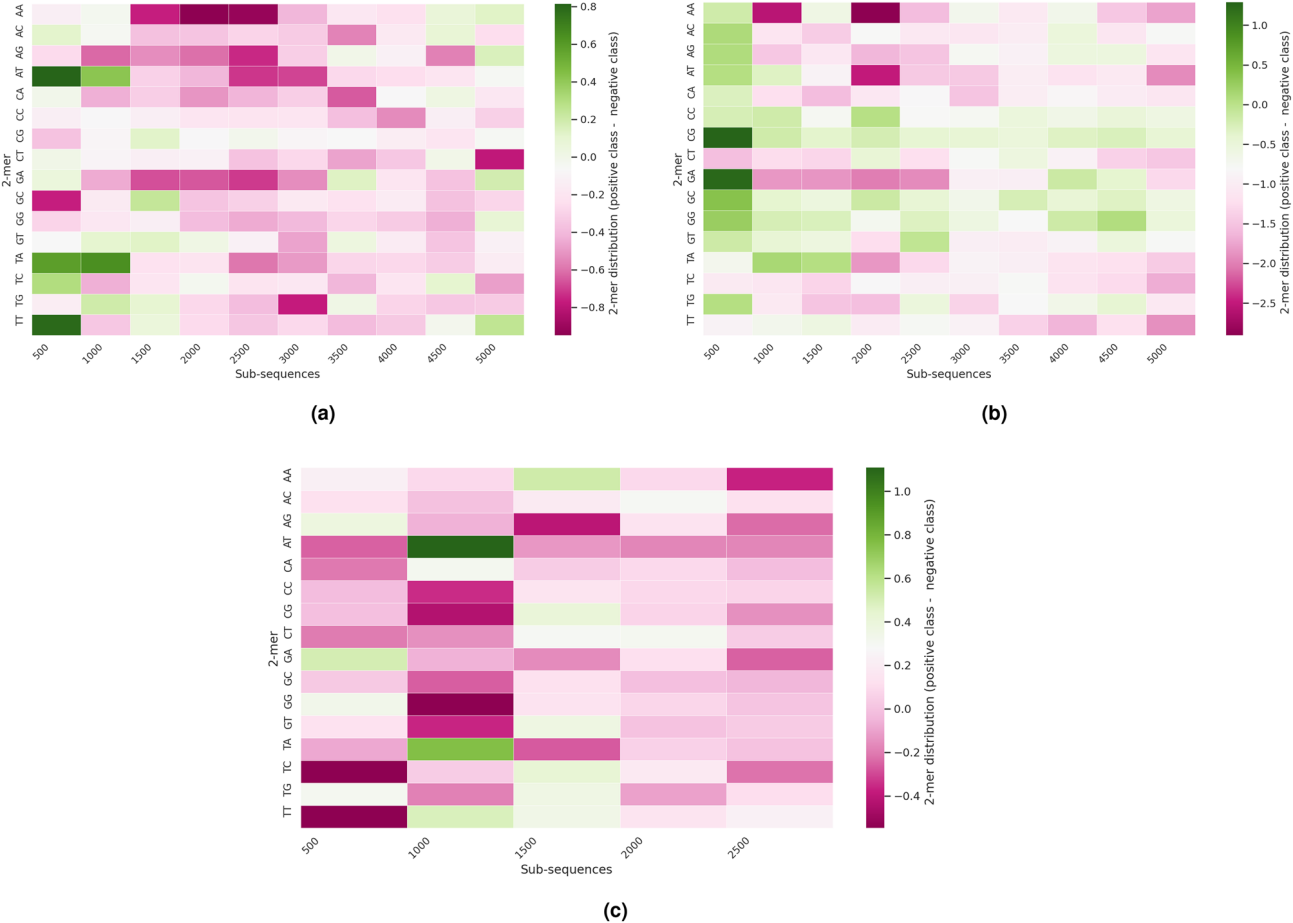
Subsequence-based density distribution of k-mers in 3 different benchmark datasets i.e. (**a**) C4-2B, (**b**) AT, and (**c**) YS.

Figure 2 shows the subsequence density-based differences of different 2-mers across 3 different leccDNA benchmark datasets namely, C4-2B, AT, and YS. The majority of density-based differences lie within the initial subsequences, whereas among the last subsequences, the densities of 2-mers are similar among leccDNA and non-leccDNA sequences. These differences in the densities of specific nucleotide pairs indicate that certain regions of the DNA sequences exhibit distinct nucleotide distribution patterns, which are characteristic of leccDNA sequences and can differentiate leccDNA from non-leccDNA sequences. Particularly certain 2-mers such as TT, TC, TA, AT, AC, GG, GC, and GA, have notably higher densities in leccDNA sequences, and 2-mers i.e., AG, GC, TG, CA, GA, and TC, show higher densities in non-leccDNA sequences. These distinguishing factors are captured with the help of specific sequence encoding methods and can be utilized for the identification of leccDNA sequences. In addition, similar patterns and 2-mer density-based differences across leccDNA and non-leccDNA sequences of other benchmark datasets are provided in the Supplementary File.

The intrinsic nucleotide pattern analysis affirms that leccDNA sequences display discernible nucleotide patterns that differentiate them from non-leccDNA sequences. These distinguishing features are predominantly situated in the initial regions of leccDNA sequences which are further investigated in the subsequent extrinsic performance analyses, as elaborated in the following subsection.

### RQ II: handling variable length of LeccDNA sequences

With an aim to analyze the impact of 5 different regions (1000, 2000, 3000, 4000, and 5000) in leccDNA identification, using 13 encoding methods and 11 classifiers based 143 predictive pipelines an extrinsic performance outcome is discussed in this section.

**Figure 3:**
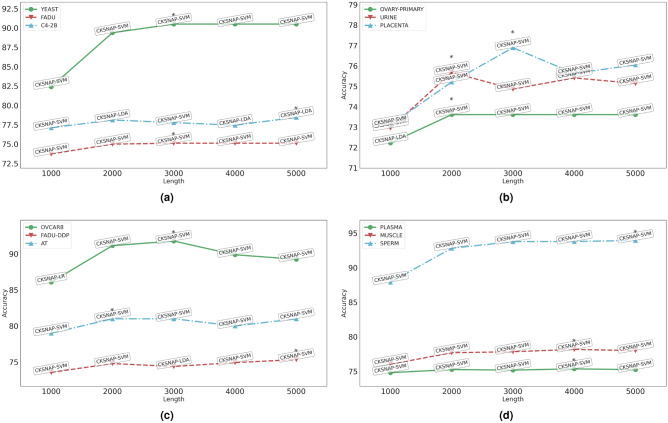
Performance comparison of 13 sequence encoding methods and 11 ML classifiers in terms of ACC over 12 leccDNA benchmark datasets at 5 different sequence lengths: 1000, 2000, 3000, 4000, and 5000.

Figure 3 illustrates top performing baseline predictors ACC values, across 5 different sequence lengths for 12 distinct benchmark datasets under independent test setting. Two different types of performance trends are observed across benchmark leccDNA datasets with respect to sequence lengths i.e., (I) steady linear performance increase with sequence length, (II) linear performance increase up to a specific sequence length. The leccDNA benchmark datasets namely, C4-2B, FAD, and SP lie under the trend category I as they have a gradual increase in performance with respect to the increase in the sequence length. These benchmark datasets have maximum performance value at the sequence length of 5000. In addition, the rest of the benchmark datasets fall under the trend category II, as the performance values increase up to a certain sequence length and afterward the performance decreases. For instance, OVCAR8, PC, and FaDu show a gradual increase in performance up to the 3000 nucleotides and a performance decline afterward. In addition, OP, UR, and AT show similar patterns until 2000 and PL, and MS show performance improvement up to 4000 nucleotides after which the performance deteriorates.

It is important to note that while dealing with leccDNA sequences, extracting meaningful information from subsequences that contain initial sequence nucleotides proves to be advantageous in achieving optimal classification efficacy. This highlights the potential benefits of breaking down complex sequences into manageable chunks, allowing for better classification performance and more efficient analysis. Furthermore, the superior performance of the baseline predictors for the initial sequence lengths reinforces the previously discussed observations that most of the discriminative patterns related to nucleotides are concentrated in the initial regions of leccDNA sequences.

### RQ III and IV: effectiveness of sequence encoding methods

**Figure 4:**
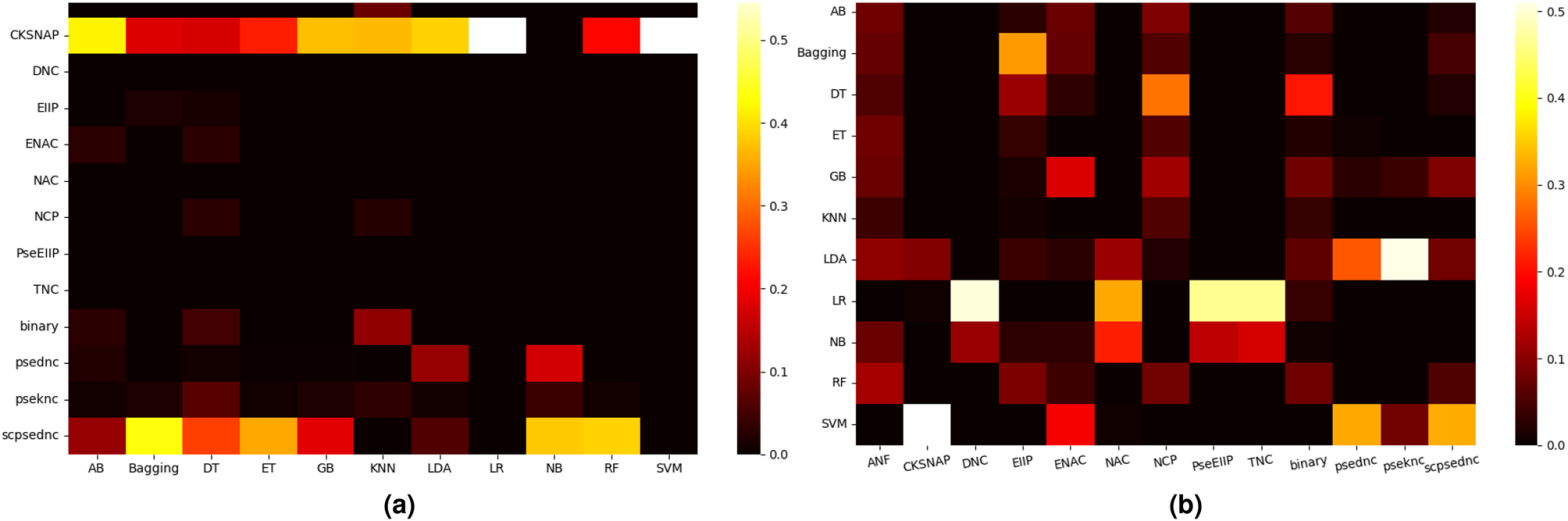
(**a**) Unraveling the top-ranked scores of 13 sequence encoding methods across 11 classifiers over multiple datasets (AT, C4-2B, OP, OV, PL, UR, and YS) and sequence lengths: 1000, 2000, 3000, 4000, and 5000. (**b**) Exploring the ranking scores of 11 ML classifiers on 13 sequence encoding methods, shedding light on their comparative performance.

This section briefly addresses research questions (III and IV) pertaining to the optimal sequence encoding methods and ML classifiers for effective leccDNA identification. To achieve this, two analyses are conducted here, first the performance rank scores of each sequence encoding method are calculated across diverse classifiers on 7 datasets. Additionally, the rank scores of classifiers are computed to identify consistently superior classifiers across all sequence encoding methods. Figure 4a shows the rank scores of 13 different sequence encoding methods with 11 ML classifiers across 7 datasets namely, AT, C4-2B, OP, OV, PL, UR, and YS. The rank scores are computed by determining the maximum performance of a sequence encoding method across a classifier for different datasets.

Three distinct categories of sequence encoders are established based on performance rank scores: (I) encoders with the lowest rank scores, (II) encoders with inconsistent performance across certain classifiers, (III) encoders with consistent performance across all classifiers. Among 13 sequence encoding methods, DNC, NAC, TNC, and EIIP fall under category I as these methods consistently show the lowest rank scores across 11 different ML classifiers. This attributes to the limited discriminatory power of these methods in capturing relevant patterns, nuanced variations, and characteristics in leccDNA sequences. Similarly, ANF, ENAC, NCP, PSEDNC and binary fall under category II as these methods show consistent ranks scores across few ML classifiers such as K-nearest neighbor (KNN), adaptive boosting (AB), decision tree (DT), Naive Bayes and linear discriminant analysis (LDA). Particularly, two sequence encoders namely, CKSNAP and SCPSEDNC lie under category III as they show consistent rank scores across majority of the classifiers. The consistent performance of these methods lies with their ability to efficiently capture nucleotide distribution and physicochemical properties that enable ML classifiers to identify leccDNA sequences with more efficacy as compared to the other sequence encoding methods.

Figure 4b shows the rank scores of 11 different ML classifiers with 13 distinct sequence encoding methods across AT, C4-2B, OP, OV, PL, UR, and YS datasets. Notably, the KNN classifier consistently exhibits the lowest predictive performance across all the sequence encoding methods. Similarly, AB classifier demonstrates inconsistent and comparatively lower performance ranks across all sequence encoding methods. Various classifiers including gradient boosting (GB), bagging, extra-trees (ET), DT, and NB exhibit relatively inconsistent performance rank scores, as they have marginal rank scores only with EIIP, NCP, and binary sequence encoding methods. On the other hand, SVM, LDA, random forest (RF) and logistic regression (LR) classifiers stand out with the highest rank scores, showing their effectiveness in the identification of leccDNA sequences with diverse sequence encoding methods.

**Table 1:** Average performance values of top performing combinations of sequence encoders with ML classifiers.

Type	Classifier	Accuracy	Sensitivity	Specificity	F1	MCC	AUC-ROC
CKSNAP-PseEIIP	SVM	**80.47**	78.14	**82.81**	**80.01**	**61.02**	**80.47**
CKSNAP-NAC	SVM	80.31	78.20	82.43	79.89	60.70	80.32
CKSNAP-PseDNC	SVM	80.02	**78.21**	81.84	79.61	60.10	80.03
CKSNAP-PseKNC	SVM	79.86	78.36	81.35	79.53	59.75	79.86
CKSNAP-TNC	SVM	79.79	77.39	82.18	79.28	59.65	79.79
CKSNAP-SCPSEDNC	SVM	79.52	77.50	81.56	79.08	59.11	79.53
CKSNAP-DNC	SVM	79.34	78.01	80.67	79.01	58.71	79.34
CKSNAP-SCPSEDNC	LDA	75.65	74.46	76.84	75.38	51.34	75.65
CKSNAP-NAC	LDA	75.39	75.48	75.30	75.55	50.86	75.39
CKSNAP-PseKNC	LDA	75.29	74.42	76.17	75.10	50.64	75.29
CKSNAP-DNC-	LDA	75.26	74.66	75.86	75.17	50.57	75.26
CKSNAP-PseDNC	LDA	74.85	74.17	75.54	74.72	49.75	74.85
SCPSEDNC-PseKNC	LDA	74.40	73.79	75.01	74.25	48.85	74.40
PseDNC-PseKNC	LDA	73.87	73.78	73.97	73.96	47.83	73.87
CKSNAP-PseEIIP	LDA	73.85	72.08	75.63	73.51	47.79	73.85
CKSNAP-TNC	LDA	73.85	72.08	75.63	73.51	47.79	73.85
CKSNAP-SCPSEDNC	GB	73.48	73.01	73.95	73.44	47.04	73.48
SCPSEDNC-PseKNC	SVM	73.27	70.35	76.18	72.47	46.63	73.27
PseDNC-SCPSEDNC	SVM	72.02	69.20	74.85	71.28	44.17	72.02
PseDNC-PseKNC	SVM	71.83	68.62	75.04	70.84	43.77	71.83

**Figure 5:**
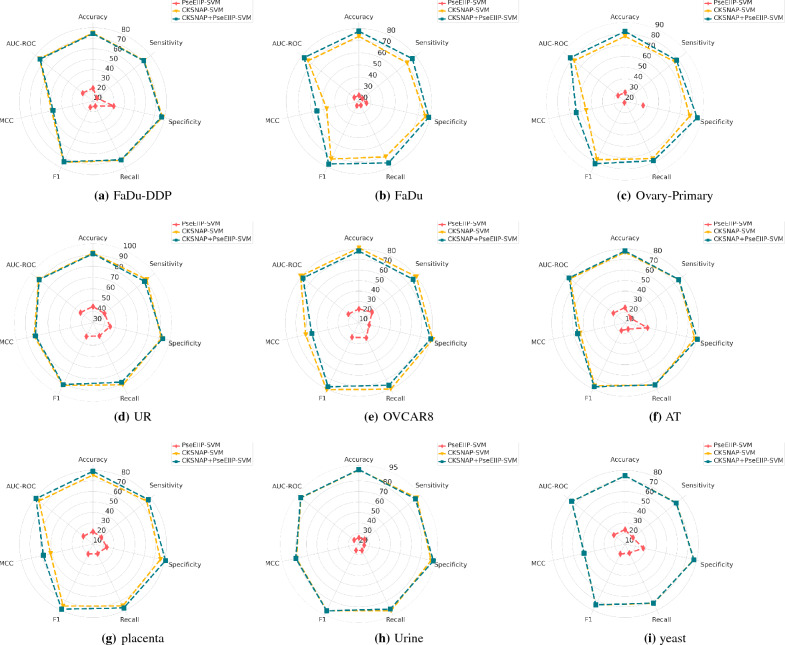
Performance scores of 9 different datasets over 1st and second stage of classification. (**a**–**i**) show the performance scores of SVM classifier over 6 distinct evaluation measures namely, ACC, SN, SP, MCC, F1, and AUC-ROC.

### RQ V: the combined potential of multiple sequence encoding methods

To address research question V, we explore the potential of 11 ML classifiers in conjunction with 78 combinations of 13 sequence encoders. The primary aim of this analysis is to identify a combination of sequence encoders that consistently demonstrate high performance in leccDNA identification. Specifically, we examine 78 unique combinations of sequence encoders paired with the 11 ML classifiers which results in approximately 858 results for a single leccDNA benchmark dataset. Additionally, due to the substantial training time involved, the analysis spans 9 different leccDNA benchmark datasets: AT, YS, C4-2B, FaDu, FAD, PC, UR, OVCAR8, and OP. Notably, 3 datasets MS, SP, and PS have been excluded from this analysis due to their large number of samples and sequence sizes.

Table 1 summarizes the average performance measures for the top 10 sequence encoders and classifier combinations which are selected based on ACC. Among 858 total combinations, CKSNAP with physicochemical properties-based sequence encoders exhibit consistent performance across 9 diverse datasets. In general, the combination of CKSNAP and the PseEIIP encoder shows the maximum performance in terms of 5 out of 6 distinct evaluation measures i.e., ACC, SP, F1, MCC, and AUC-ROC. Particularly, CKSNAP-PseEIIP combination achieves superior performance as compared to 19 other top performing combinations with average performance margins of 4.583 % in terms of ACC, 5.436% over SP, 4.451% in terms of F1, 9.71% over MCC and 4.58% over AUC-ROC. It is important to mention that the combination of CKSNAP and PseEIIP produces maximum performance with SVM classifier. Hence, CKSNAP-PseEIIP combination and SVM are selected for the final performance analyses across all benchmark leccDNA datasets. Finally, 858 different results for 78 encoder combinations across 11 ML classifiers in terms of 6 distinct evaluation measures are provided in Supplementary Files.

### RQ VI: the combined potential of CKSNAP and PseEIIP

To find the answer to research question VI, we explore the potential of CKSNAP and PseEIIP with SVM classifier. Here the objective is to analyze whether SVM produces better performance with statistical vectors of standalone encoders or with their combined statistical vectors. Figure 5a–i showcases the evaluation results based on performance values generated by standalone and combined statistical representations with SVM classifiers. It highlights the performance gains achieved through the utilization of diverse discriminatory features from CKSNAP and PseEIIP with SVM classifier across 9 different leccDNA benchmark datasets.

Out of 9 different benchmark leccDNA datasets, the combined potential of CKSNAP and PseEIIP shows performance improvements over 7 different datasets except AT and FaDu. Particularly, it shows average perfromance gains of 1.5 % in terms of ACC, 1.466 % and 1.97 % across SN and SP, 1.457% and 2.88% in terms of F1 and MCC, and 1.50% across AUCROC. Particularly, in terms of AT and FaDu datasets, CKSNAP along with SVM classifier manages to produce better performance as compared to CKSNAP and PseEIIP combination across 6 distinct evaluation measures i.e., ACC, SP, SN, MCC, F1, and AUC-ROC.

**Figure 6:**
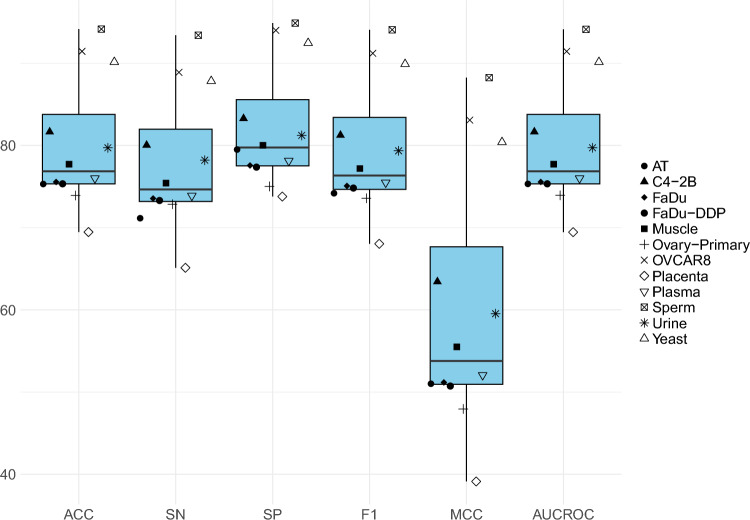
Performance values of the proposed predictor across 12 different leccDNA datasets in terms of  6 distinct evaluation measures in terms of 5-fold validation.

**Figure 7:**
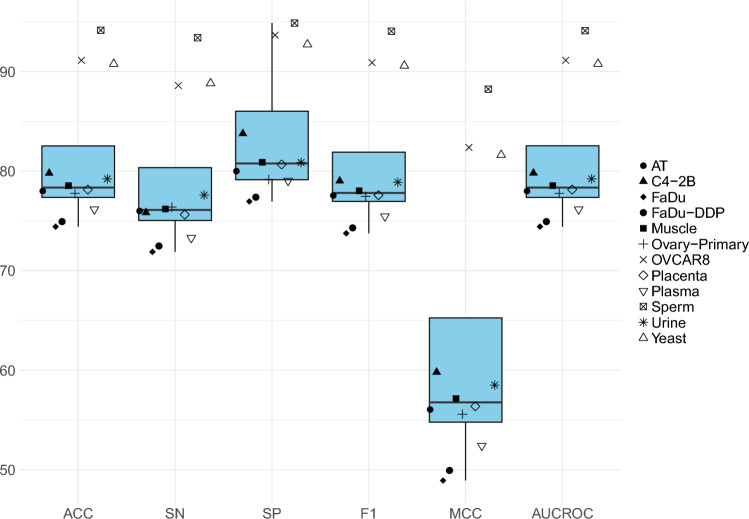
Performance values of the proposed predictor across 12 different leccDNA datasets over 6 distinct evaluation measures in terms of the independent test set.

Overall, it is observed that both sequence encoding methods provide unique and discriminatory information to the classifiers for leccDNA identification. This discriminatory and unique information when presented to the ML classifiers in a concatenated way leads to signification performance gains which suggests the importance of using multiple sets of information while training a classifier for leccDNA identification. Therefore, the final experimentation over leccDNA benchmark datasets is performed by utilizing SVM, CKSNAP, and PseEIIP.

### Performance analyses over 5-fold cross validation

Figure 6 illustrates the performance results of the proposed leccDNA predictor in terms of 5-fold cross-validation across 12 benchmark leccDNA datasets. The proposed predictor shows high-performance values over the dataset of SP, OVCAR8, and YS ranging from 90-94$\%$ in terms of ACC, and AUC-ROC. For OVCAR8, SP and YS datasets there is an average gap of 3.73% in terms of SP and SN, which suggests that the proposed predictor is less prone to type I and type II error. This implies that the proposed leccDNA predictor is quite robust in predicting samples belonging to positive and negative classes. The high performance of the proposed predictor is due to the sufficient number of samples present to train the proposed predictor.

In addition, across the datasets of OP, FaDu, PL, MS, UR, C4-2B, and FAD, the performance of the proposed predictor ranges from 75-82% in terms of ACC and AUC-ROC. The proposed predictor is not highly prone to type I and type II errors due to an average gap of 4% among SP and SN values. Moreover, the proposed predictor shows low performance on the PC and AT datasets with the performance values ranging from 69-73% in terms of ACC and AUC-ROC. In addition, over both of the datasets, the proposed predictor is prone to either type I or type II error due to an average gap of more than 5% in terms of SP and SN and lower AUC-ROC. The predictor is more prone to type II error as it is not able to successfully identify positive samples with a higher ratio as compared to the negative samples. The low performance of the proposed predictor on these datasets is due to the presence of a limited number of samples for positive and negative class samples (200 leccDNA sequences for AT and 400 leccDNA sequences in terms of PC).

### Performance analyses over independent test set

Figure 7 illustrates the performance analyses over 6 distinct evaluation measures across 12 different benchmark datasets in terms of independent test sets. A closer look at the performance values at the 5-fold validation and independent test sets reveals that the performance of the proposed predictor either remains the same, increases, or decreases as compared to the 5-fold validation across various datasets. The performance of the proposed predictor remains the same across 6 different datasets such as FaDu, FAD, MS, YS, PL, and OVCAR8. Similarly, there is a slight decrease in the performance of the proposed predictor over C4-2B dataset, and an increase in the performance over 4 datasets namely, MS, PC, AT and OP.

### Webserver

This article performs experimentation on 12 benchmark leccDNA datasets of 3 different species. To facilitate readers, we have provided all 12 benchmark datasets in the download section of our leccDNA prediction web application (https://sds_genetic_analysis.opendfki.de/iLEC_DNA/). In addition, users can train iLec-DNA on different datasets by using the training module of the web application.

## Discussion

Intrinsic and extrinsic performance analyses of experimental results reveal that initial regions of leccDNA sequences carry significant discriminatory information for leccDNA identification. In addition, experimental results on 12 benchmark datasets from 3 different species, reveal that among 13 diverse types of encoding methods, two encoders CKSNAP and PseEIIP generate more comprehensive statistical vectors. A prime reason behind generating better statistical vectors is the extraction of both simple as well as gap-based nucleotide patterns. Specifically, CKSNAP encoder transforms raw DNA sequences into statistical vectors by computing occurrence distribution of simple as well as gap-based bimers. PseEIIP encoder makes used of predefined electron ionic pseudo potentials of trinucleotides, along with their frequency. In a nutshell, it can be concluded that those encoding methods are more suitable for transforming raw DNA sequences into statistical vectors that emphasize on gap-based nucleotides distribution. Furthermore, the ensembling of both encoders generates better statistical vectors. Primarily, the concatenation of statistical vectors of both encoders facilitates the extraction of two different types of features, gap based bimers distribution and gap-based nucleotides correlational information extracted through physicochemical properties. Among 11 different classifiers, SVM remains top performer because it finds optimal hyperplanes for discriminating sequences into leccDNA and non-leccDNA classes. Experimental results reveal that across all species benchmark datasets, it successfully designed optimal hyperplanes. Although several classifiers such as RF, LDA, and LR produce SVM comparable performance on a few datasets, overall they fail to produce consistent performance across all datasets.

Although the incorporation of distinct physicochemical, and nucleotide distribution information achieves a notable reduction in prediction errors across diverse leccDNA datasets, the robustness and efficacy of the proposed model are limited over the AT and PC datasets due to a bias towards type II errors. The proposed predictor suffers from this problem due to the availability of a lower number of leccDNA sequences across these two benchmark datasets. In the future, we intend to leverage additional sequence encoding methods, feature selection methods and incorporate certain deep learning models to enhance the classification efficacy and robustness across diverse leccDNA datasets. Moreover, by following the criteria of existing sequence analysis tools, hyperparameters of ML models can be optimized to further improve the predictive performance^33–37.^

## Conclusion

The primary objective of this study is to introduce AI-based web application that can accurately predict leccDNA in various cell types and species. With an aim to develop a powerful web application, it presents 12 benchmark datasets that are utilized to evaluate the performance of leccDNA predictive pipelines and for the development of web application. In addition, the unique distribution of nucleotides is explored with an aim to decode the discriminatory potential in leccDNA sequences. To design a robust and precise leccDNA predictive pipeline, it explores the potential of 13 different sequence encoding methods in conjunction with 11 ML classifiers. Comprehensive experimentation across 12 benchmark datasets reveals that SVM classifier and 2 sequence encoding methods namely, PseEIIP and CKSNAP give superior and consistent performance across diverse leccDNA datasets. Furthermore, the concatenation of statistical vectors generated through CKSNAP and PseEIIP leads to significant performance gains. On top of the proposed predictor, the web application is developed that will facilitate biological researchers for conducting more comprehensive research for leccDNAs. In future, we will further enhance the scope of application by collecting data related to other species such as Drosophila (DM), Chimpanzee (CH), and Mouse (MM). We will perform cross-species analysis, where the model is trained on one species (AT, YS, DM, CH, MM) and evaluated on other species (HM), this will help in identifying leccDNA from other species at a larger scale. Based on various performance analysis, iLEC-DNA, a novel predictor for leccDNA, is proposed that captures discriminatory information through pseudo ionic potentials and nucleotide distribution information. iLEC-DNA is evaluated over 12 distinct benchmark datastes namely, MS, PS, SP, FaDU, FAD, PC, UR, CB, OV, OP, AT and YS. iLEC-DNA is a valuable tool for researchers examining intricate and lengthy eccDNA. Its capabilities can enable the exploration of leccDNA and their involvement in genomic instability and the onset of cancer.

## Materials and methods

This section demonstrates comprehensive details of proposed and baseline predictors. It provides a comprehensive overview of benchmark datasets development process and characteristics of datasets. Finally, it presents evaluation measures that are used to evaluate and compare the performance of proposed and baseline predictors.

**Figure 8:**
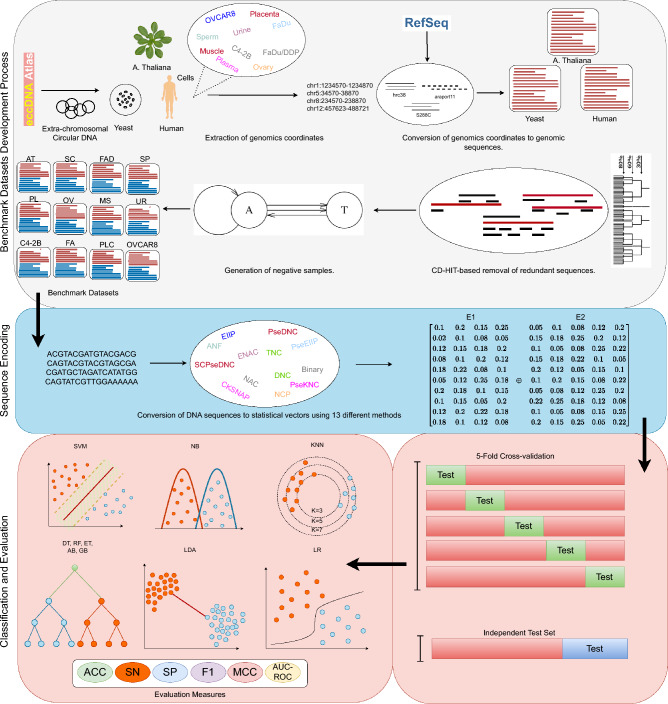
Graphical illustration of Benchmark datasets development process, proposed and baseline predictors. In datasets development process, leccDNA sequences are extracted from the eccDNA atlas and CD-HIT is utilized to remove redundant leccDNA sequences. Subsequently, USHUFFLE is applied to generate negative samples. In the second step, leccDNA sequences are converted into statistical vectors through baseline and proposed sequence encoding pipelines. In the classification and evaluation process, the performance of the proposed predictor is compared with the baseline predictor across all datasets.

### Summary of the LeccDNA identification predictive pipeline

Figure 8 demonstrates different modules of the proposed iLEC-DNA predictor. It can be seen that after the datasets development process, DNA sequences are transformed into statistical vectors. The transformation of DNA sequences into statistical vectors is an essential task because AI predictors can only process numerical data and cannot operate directly on DNA sequences. While converting DNA sequences into statistical vectors the prime objective is to incorporate sequence order, semantic, nucleotide distribution, and positional features into the statistical vectors. First, the potential of 13 sequence encoding methods  along with 11 different ML classifiers is explored to identify the most consistent sequence encoder and ML classifier from 143 total combinations. In the subsequent step, 858 ensembles of encoders (78 encoder combinations $\times$ 11 ML classifiers) are created to reap the benefits of discriminative and unique information from multiple encoders. Our analysis shows that leccDNA sequences can be converted to discriminative statistical vectors by reaping the combined benefits of two different types of sequence encoding methods namely, CKSNAP and PseEIIP . Finally, the concatenated representations are passed to SVM classifier which shows superior performance as compared to any other combination. A comprehensive description of both sequence encoding methods and their concatenation is provided following subsections.

### Benchmark datasets

In the pursuit of creating effective and reliable machine learning (ML) predictors for any biological sequence analysis tasks, the selection of appropriate data is a crucial task^38^. Inappropriate data can lead to the development of a biased and unreliable predictor that results in misleading insights and flawed decision-making.

EccDNA sequences are available across various databases such as eccDNA Atlas^39^, TeCD^40^, EccBase^41^, EccDB^42^, and EccDNADB^43^. Each database includes extrachromosomal DNA sequences of different species and cells. Among all databases, eccDNA Atlas^39^ offers a vast and comprehensive collection of eccDNA sequences derived from diverse organisms and experimental techniques. This extensive coverage ensures a broader representation of eccDNA diversity, enabling researchers to access a more complete picture of eccDNA characteristics across different species and experimental conditions.

To prepare leccDNA identification data, first necessary details such as specie, tissue, cell, isolate, genome version, and genomic coordinates, related to leccDNA sequences are acquired from eccDNA atlas database. Specifically, the genomic coordinates encompass information such as chromosome number, start and end positions of the leccDNA sequences. In addition, the genome versions/assemblies are downloaded from Refseq (https://www.ncbi.nlm.nih.gov/refseq/)^44^, namely araport11, S288C, and hrc38. In the subsequent step, the genomic coordinates and assemblies are utilized to retrieve relevant leccDNA sequences for diverse types of species. A summary of statistics related to obtained eccDNA sequences of 3 different species i.e., *Saccharomyces cerevisiae* (SC), *Arabidopsis thaliana* (AT), and *Homo sapiens* (HM) is presented in Table 2.

Table 2 provides statistics of 12 different benchmark leccDNA datasets. Among 12 benchmark leccDNA datasets, 10 datasets belong to different cell lines of HM namely, muscle (MS), plasma (PS), sperm (SP), FaDU, FaDU-DDP (FAD), placenta (PC), urine (UR), C4-2B (CB), ovcar-8 (OV), and ovary-primary (OP). Due to the low number of samples in terms of tissues/isolates of species AT and YS, only two datasets are formulated from them.

After the retrieval of leccDNA sequences, the leccDNA sequences may contain redundant or highly similar sequences. However, these similarities can introduce a bias when dividing the data into training and testing sets, leading to an overestimation of the model performance and the establishment of impractical benchmarks. To create reliable and comprehensive benchmark datasets, following previous studies^45–47^ we apply CD-HIT (sequence similarity >60$\%$ ) to positive samples, where redundant or highly similar sequences are clustered together, resulting in a representative subset that encompasses the essential sequence variations. This process helps to prevent the over-representation of certain sequences, which could introduce biases during the training process of the ML classifier.

**Table 2:** Statistics for leccDNA and non-leccDNA sequences across 12 different benchmark datasets.

		AT	C4-2B	FADu	FADu-DDP	Muscle	Ovary-Primary	OVCAR8	Placenta	Plasma	Urine	Yeast	Sperm
Train	Positive	201	598	1739	1472	9143	289	317	477	10232	736	716	9659
	Negative	201	598	1739	1472	9143	289	317	477	10232	736	716	9659
	Total	402	1196	3478	2944	18286	578	634	954	20464	1472	1432	19318
Test	Positive	50	149	434	367	2285	72	79	119	2557	183	179	2414
	Negative	50	149	434	367	2285	72	79	119	2557	183	179	2414
	Total	100	298	868	734	4570	144	158	238	5114	366	358	4828

There are multiple ways to generate negative data samples for a DNA sequence classification task i.e., selection of sequences from genomic background^48,49^, and nucleotide shuffling^49,50^. For instance, sequences are randomly sampled from different positions of a genome to get a diverse pool of negative samples that are non-overlapping to the positive samples. In addition, sometimes negative samples are clustered with positive samples using psi-cd-hit to remove closely related positive and negative samples. In spite of its usage, this method has various disadvantages i.e., compositional bias, where the distribution of nucleotides in negative samples might differ completely as compared to positive samples which may lead to biased training of the ML models. In comparison, nucleotide shuffling tackles such problems by preserving various k-mers counts. Ushuffle is designed to preserve the statistical properties and local sequence features of the input sequences while removing specific sequence motifs and patterns. Following the existing work^49^, fasta$\_$ushuffle (k=2)(https://github.com/agordon/fasta_ushuffle) is utilized to shuffle nucleotides in positive samples to obtain suitable negative samples.

**Figure 9:**
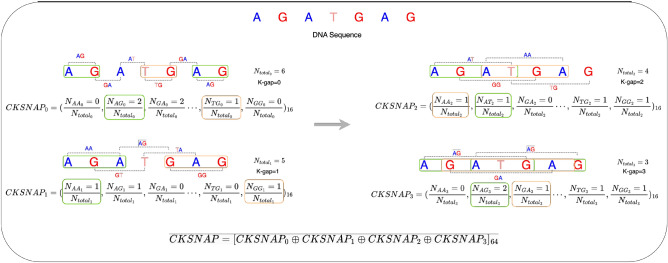
Working paradigm of CKSNAP sequence encoding method on a hypothetical DNA sequence i.e., AGATGAG with k-gap = 3.

#### Complementary K-spaced nucleic acid pairs (CKSNAP)

CKSNAP encoder was proposed by Zhang et al.^51^ and has been widely used used in diverse types of DNA sequence classification predictors including, enhancer prediction^52^, DNA replication origin identification^53^, DNA modification prediction^54^ and promoter prediction^55^. The motivation behind the development of this encoder was to capture nucleotide occurrence distribution patterns with different gap values. CKSNAP^56^ generates gap based bimers such as for a hypothetical sequence GCTA, with gap value 1, gap-based bimers are be G-T and C-A. Similarly, for gap value 2, it first generates 1 gap-based bimers and then 2 gap-based bimers. Furthermore, for each gap value, it computes occurrence frequencies of bimers and normalizes them with total number of gap-kmers. Mathematically, CKNSAP can be written as;

1 $$\begin{aligned} CKSNAP = \left( \frac{N_{AA}}{N_{total}}, \frac{N_{AG}}{N_{total}}, \frac{N_{AT}}{N_{total}}, \ldots , \frac{N_{GC}}{N_{total}}, \frac{N_{GT}}{N_{total}} \right) _{16}, \end{aligned}$$

where, N_AA_ represents the total occurrences of bimer AA in the DNA sequence and N_total_ denotes the total number of gap bimers. A detailed working paradigm of CKSNAP is shown in Fig. 9.

### Electron-ion interaction pseudopotential of trinucleotides (PseEIIP)

Nair et al.,^57^ proposed electron ionic potential of 4 different nucleotides i.e., A, G, C, T (A: 0.1260, C: 0.1340, G: 0.0806, T:0.1335). These values represent the potential energy of the electrons and ions present in the atoms of the nucleotide. PseEIIP incorporates electron ionic potentials and nucleotide frequency of trinucleotides and converts a DNA sequence into a statistical representation.

To generate statistical representation through PseEIIP, first 3-mers dictionary is computed from a DNA sequence,

2 $$\begin{aligned} 3-mers = [N_{AAA}, N_{ATA}, N_{ATC}, \ldots , N_{TTT}]_{64} \end{aligned}$$

In the subsequent step, these values are normalized based on the total number of 3-mers in the DNA sequence,

3 $$\begin{aligned} f_{3-mers} = [N_{AAA}/T_k, N_{ATA}/T_k, N_{ATC}/T_k, \ldots , N_{TTT}/T_k]_{64} \end{aligned}$$

where $T_k$ denotes the total number of 3-mers in a DNA sequence. In addition, electron ionic potential values of 3-mers are summed and multiplied with corresponding normalized frequencies i.e.,

4 $$\begin{aligned} V = [E_{AAA}. f_{AAA}, E_{ATA}. f_{ATA}, E_{ATC}. f_{ATC}, \ldots , E_{TTT}. f_{TTT} ]_{64} \end{aligned}$$

whereas, $E_{AAA}$ represents the sum of ionic potentials for A, and $E_{AAA}$ denotes the sum of ionic potentials for A, T, and A.

#### Feature fusion

Feature fusion involves the integration of diverse types of sequence information into a single vector which can improve the discriminative potential of statistical vectors and the efficacy of an AI predictor. Diverse types of feature fusion methods have been utilized to improve the performance of various sequence analysis tasks such as DNA hypersensitive site prediction^58^, DNA modification prediction^59^, promoter prediction^60^, and DNA binding proteins identification^61^.

In pursuit of harnessing the combined benefits of the two distinct sequence encoding methods, an early fusion strategy based on vector concatenation is adopted in the proposed iLEC-DNA predictor. Let $\overrightarrow {X}$ and $\overrightarrow {Y}$ be represented as statistical vectors of dimensions P and Q for a given sequence S:

5 $$\begin{aligned} \overrightarrow {X} = [x_1, x_2, x_3, \ldots , x_p]\, \& \, \overrightarrow {Y} = [y_1, y_2, y_3, \ldots , y_q] \end{aligned}$$

Subsequently, the fused vector can be expressed as:

6 $$\begin{aligned} \overrightarrow {F} = \overrightarrow {X} \oplus \overrightarrow {Y} = [x_1, x_2, x_3, \ldots , x_p, y_1, y_2, y_3, \ldots , y_q] \end{aligned}$$

where, $\overrightarrow {F}$ represents p$+$q dimensional fused vector.

### Baseline predictors

This section summarizes 11 remaining encoders namely, nucleic acid composition (NAC)^62^, enhanced nucleic acid composition (ENAC)^63^, accumulated nucleotide frequency (ANF)^64^, dinucleotide composition (DNC)^65^, trinucleotide composition (TNC)^66^, nucleotide chemical property (NCP)^67^, binary^68^, electron ionic interaction potential (EIIP)^57^, series correlation pseudo dinucleotide composition (SCPseDNC),^69^, pseudo dinucleotide composition (PSEDNC)^70,71^, and pseudo k-tupler composition (PSEKNC)^72^.

Nucleic acid composition (NAC)^62^ computes the normalized frequency of each nucleotide across the DNA sequence. The normalization is done through the total length of the DNA sequence. Similarly, dinucleotide composition (DNC)^65^ and trinucleotide composition (TNC)^66^, use the pairs of nucleotides (k = 2, or k = 3) to compute normalized occurrence frequencies rather than taking into account individual nucleotides. Enhanced nucleic acid composition (ENAC)^63^ transforms raw sequences into statistical vectors by counting the number of different k-mers at a fixed sliding window. First, a dictionary of unique k-mers is created and then for each unique each k-mer, within each window its count is computed. This step is repeated by sliding over the DNA sequences with a step size of $W_S$. In the end, all the count dictionaries are concatenated together to form a discriminative statistical vector.

Accumulated nucleotide frequency (ANF)^64^ encodes nucleotide frequency information in the statistical vectors. First, it computes the position-wise counts of nucleotides and then normalizes it with the position of nucleotides. Then, it represents each nucleotide with a 4-dimensional vector at each position, where the first three values indicate the presence or absence of a specific nucleotide, and the last value is the normalized positional density of that specific nucleotide. In the binary^68^ sequence encoding method, each nucleotide is represented by a vector of size 4. These vectors include ones and zeros with each one representing the presence of a specific nucleotide.

The nucleotides of the DNA have different chemical structures and chemical properties. Physicochemical properties-based sequence encoding methods make use of such information to capture discriminative information from the raw DNA sequences. Nucleotide chemical property (NCP)^67^, converts DNA sequences into statistical vectors based on the ring structure, functional group, and hydrogen group where each nucleotide is represented by a 3-dimensional vector. Electron-Ion Interaction Potential (EIIP)^57^ makes use of numerical values based on the average interaction potential between nucleotides constituent atoms, and electrons. It converts DNA sequences into statistical vectors by substituting each nucleotide with the predefined ionic potential value. Electron-ion interaction pseudopotentials of trinucleotide (PseEIIP)^69^ utilizes electronic ionic potential values of trinucleotides and their normalized occurrence frequency. For a trinucleotide, first the ionic potential is computed by summing up the individual pseudo-ionic potential values of three nucleotides which is multiplied by the normalized occurrence frequency of that specific trinucleotide.

Pseudo dinucleotide composition (PseDNC)^70,71^ makes use of six distinct DNA properties i.e., twist, roll, rise, tilt, shift, and slide, along with the frequencies of the nucleotide pairs. First, normalized occurrence frequencies of nucleotide pairs are computed which encode the contiguous local sequence-order information of the DNA sequence. To include the global sequence-order information, a set of correlation functions are computed among the neighboring nucleotides. These functions are computed by taking the mean over the difference among the nucleotide pairs property values. The output of pseDNC is a (16+$\lambda$)-D vector, where the first 16 values represent the normalized frequencies of nucleotide pairs and the rest are higher-order correlation functions. Pseudo k-tupler Composition (PseKNC)^72^ works on a similar principle but the difference lies in K-tuple composition used in PseKNC. Rather than dealing only with dinucleotides or trinucleotides, PseKNC makes use of K  = (1 $\ldots$, L), to compute statistical vectors that contain higher and lower order features.

### Evaluation measures

Following evaluation criteria of existing DNA sequence classification predictors^23–25,27^, we evaluate proposed and baseline predictors using five different evaluation measures namely, accuracy (ACC), sensitivity (SN), specificity (SP), Mathews correlation coefficeint (MCC), and area under the receiver operating curve (AUC-ROC).

Accuracy^26^ refers to the proportion of correct predictions with respect to the total predictions. Specificity or true negative rate (TNR)^23^ is the model’s ability to correctly predict the negative class samples. It is determined by dividing the number of correct negative predictions by the total number of true negatives. Sensitivity (or recall)^26^ measures the ability of the model to predict positive class samples by taking the ratio of correct positive predictions to the predictions on positive samples. MCC^73^ calculates the correlation between the model predictions and the true class, by taking into consideration true positives, true negatives, false positives, and false negatives. AUC-ROC^74^ computes the degree of separability of the model based on the true positive rate (TPR) and true negative rate (TNR) at various thresholds.

7 $$\begin{aligned} f(x)={\left\{ \begin{array}{ll} \begin{aligned}\text {Accuracy (ACC)}= (T^++T^-)/(T^++T^-+F^++F^-) \end{aligned} \\ \begin{aligned}\text {Specitivity (SP)} = T^-/(T^-+F^+) \end{aligned} \\ \begin{aligned}\text {Sensitivity (SN)} = T^+/(T^++F^-) \end{aligned} \\ \begin{aligned} \text {False Positive Rate (FPR)} = F^+/(T^-+F^+) \end{aligned}\\ \begin{aligned} \text {MCC} = T^+ \times T^- - F^+ \times F^-/Z \end{aligned}\\ \begin{aligned} \text {Z} =\sqrt{(T^++F^-)(T^++F^+)(T^-+F^+)(T^-+F^-)} \end{aligned}\\ \end{array}\right. } \end{aligned}$$

In the mathematical expression above, $T^+$ and $T^-$ denote the true predictions related to positive and negative classes, whereas $F^+$ and $F^-$ are the incorrect predictions related to the positive and negative classes respectively.

**Table 3:** Parameters values for 11 different ML classifiers used for LeccDNA identification.

Classifier	Parameters
LR	penalty=’l2’, C=1.0, max_iter=100, solver=’lbfgs’
KNN	n_neighbors=5
DT	criterion=’gini’, splitter=’best’, max_depth=None
NB	var_smoothingfloat=1e-9
Bagging	base_estimator=None, n_estimators=10, max_samples=1.0, max_features=1.0
RF	n_estimators=100, criterion=’gini’, max_depth=None, min_samples_split=2, min_samples_leaf=1, max_features=’auto’
AB	base_estimator=None, n_estimators=50, learning_rate=1.0
GB	loss=’deviance’, learning_rate=0.1, n_estimators=100, subsample=1.0, criterion=’friedman_mse’, min_samples_split=2, min_samples_leaf=1, max_depth=3
LDA	solver=’svd’, shrinkage=None
SVM	C=1.0, kernel=’rbf’, degree=3, gamma=’scale’, probability=False
ET	n_estimators=100, criterion=’gini’, max_depth=None, min_samples_split=2, min_samples_leaf=1, max_features=’auto’

### Experimental setup

To prepare benchmark datasets, we utilize two different APIs namely, Biopython^75^ and USHUFFLE^50^. The proposed and baseline predictive pipelines are developed on top of two libraries namely, iLearnPlus^76^ (https://ilearnplus.erc.monash.edu/) and Scikit-Learn^77^ v1.3.2^77^ (https://scikit-learn.org/stable/ Following the evaluation criteria of existing DNA sequence classification predictors^23–25,27^, we perform experimentation in two different settings namely, 5-fold cross-validation and independent test set. All visualizations are generated using matplotlib v3.8.0^78^ (https://matplotlib.org/). The parameter values for 11 different ML classifiers are provided in Table 3.

### Supplementary Information

Supplementary Information.

## Data Availability

The datasets generated and analysed during the current study are available in the following Github repository Extrachrosmosomal-DNA-Prediction, [https://github.com/FAhtisham/Extrachrosmosomal-DNA-Prediction].
