# Supplementary material for: Long extrachromosomal circular DNA identification by fusing sequence-derived features of physicochemical properties and nucleotide distribution patterns
Source: Sci Rep. 2024 Apr 24;14:9466. doi: 10.1038/s41598-024-57457-5 (PMC11043385; doi:10.1038/s41598-024-57457-5)
Supplement: Supplementary file 1 — Supplementary Information. [file 41598_2024_57457_MOESM1_ESM.pdf]

Supplementary Information iLEC-DNA

1. Steps to identify information rich regions

Information rich regions are defined based on the following steps:

1. Let K = 2 be a predetermined value, that represents nucleotide bimers.

2. Create a set of k-mers denoted as S = {AA, AT, AG, ..., GG} containing a total of 16 unique elements.

3. Divide the DNA sequence into 10 equal-length segments: [(0, 500), (500, 1000), (1000, 1500), ..., (4500, 5000)].

   -Denote the segments as {s1, s2, ..., s10}.

4. Create a matrix M of size (10, 16) where Mij represents the count of the j-th k-mer in the i-th segment.

5. Iterate through each segment si of the DNA sequence:

   - For each k-mer kj in the set S, calculate the frequency fij in si and update Mij.

6. Repeat the process for negative sequences, resulting in a negative matrix N.

7. Normalize both the positive matrix M and the negative matrix N by dividing each element by the maximum DNA sequence length L = 5000:

   Mij = Mij / L

   Nij = Nij / L

8. Calculate the difference matrix D as the element-wise subtraction of the positive matrix M from the negative matrix N:

   Dij = Nij - Mij

9. Visualize the difference matrix using heatmaps.

2-mer Distribution Over Remaining Datasets

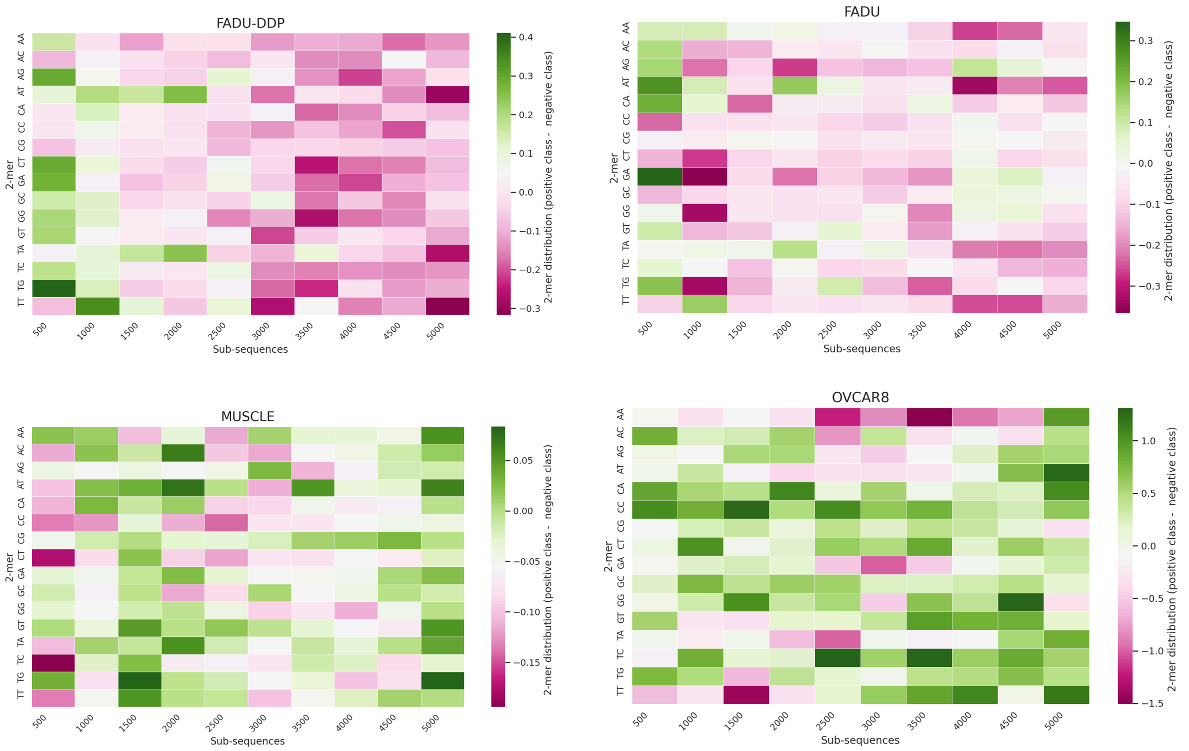

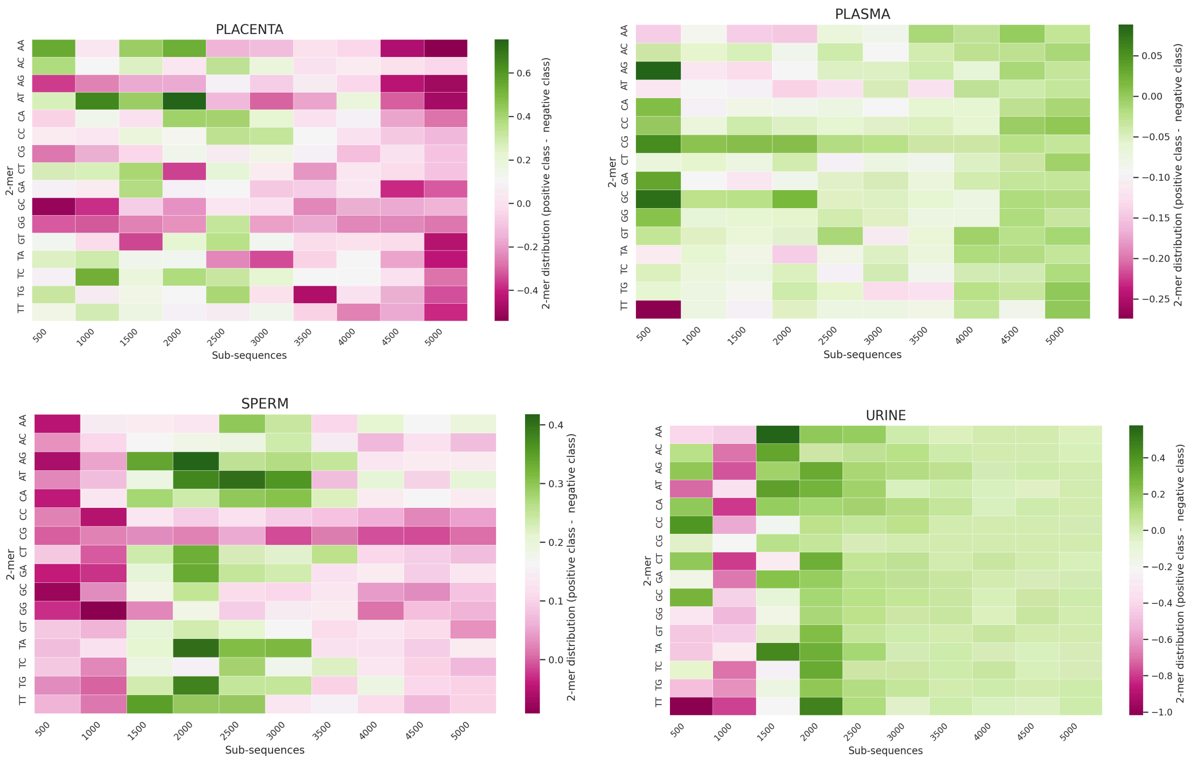

Table S1 Performance Values of More than 800 Ens.

Type	Accuracy	Sensitivity	Specificity	Recall	F1	MCC	AUC-ROC	Avg-Precision
CKSNAP-NAC-SVM	80.31366666666666	78.19722222222222	82.43311111111112	78.19722222222222	79.89111111111112	60.69588888888895	80.31511111111111	75.13733333333333
CKSNAP-psednc-SVM	80.02466666666666	78.20688888888888	81.84488888888889	78.20688888888888	79.611	60.09999999999994	80.02577777777778	74.72855555555556
CKSNAP-pseknc-SVM	79.85622222222223	78.36122222222222	81.35333333333334	78.36122222222222	79.52644444444445	59.74822222222225	79.85733333333333	74.524
CKSNAP-TNC-SVM	79.78588888888889	77.39066666666666	82.18388888888889	77.39066666666666	79.28366666666666	59.65066666666666	79.78722222222223	74.63788888888888
CKSNAP-DNC-SVM	79.33766666666666	78.00844444444445	80.66811111111112	78.00844444444445	79.00511111111112	58.708111111111116	79.33833333333334	73.89777777777778

CKSNAP-scpsednc-LDA	75.64922222222224	74.45633333333333	76.84333333333333	74.45633333333333	75.38055555555555	51.33577777777778	75.64977777777777	70.0408888888889
CKSNAP-NAC-LDA	75.39411111111112	75.48477777777777	75.30355555555555	75.48477777777777	75.54533333333333	50.86377777777778	75.39433333333334	70.059
CKSNAP-pseknc-LDA	75.29866666666666	74.42766666666667	76.17122222222223	74.42766666666667	75.10733333333333	50.64533333333333	75.29944444444445	69.87577777777778
CKSNAP-DNC-LDA	75.25666666666666	74.65511111111111	75.85944444444445	74.65511111111111	75.17433333333332	50.56666666666667	75.2571111111111	69.90977777777778
CKSNAP-psednc-LDA	74.85211111111111	74.16822222222223	75.53644444444444	74.16822222222223	74.71677777777778	49.7458888888889	74.85233333333332	69.52166666666666
scpsednc-pseknc-LDA	74.39755555555556	73.78622222222222	75.00733333333334	73.78622222222222	74.25255555555556	48.84711111111111	74.3968888888889	68.82844444444444
psednc-pseknc-LDA	73.87388888888889	73.77833333333334	73.96844444444444	73.77833333333334	73.96244444444444	47.82566666666666	73.87344444444444	68.47300000000001
CKSNAP-scpsednc-GB	73.48188888888889	73.01377777777778	73.94822222222223	73.01377777777778	73.43655555555556	47.04044444444445	73.48077777777777	67.89833333333334
scpsednc-pseknc-SVM	73.26788888888888	70.35411111111111	76.18433333333333	70.35411111111111	72.47177777777777	46.63233333333335	73.26922222222223	68.01533333333333
psednc-pseknc-SVM	71.83177777777777	68.6231111111111	75.04255555555557	68.6231111111111	70.83999999999999	43.77122222222222	71.83266666666667	66.59122222222223
CKSNAP-DNC-GB	71.76211111111111	70.12700000000001	73.39744444444445	70.12700000000001	71.33244444444443	43.56455555555556	71.76233333333333	66.35222222222222
psednc-NAC-LDA	71.69855555555556	69.37377777777778	74.023	69.37377777777778	71.08022222222222	43.49811111111115	71.69833333333332	66.27377777777777
CKSNAP-scpsednc-AB	71.60655555555554	71.56844444444444	71.64322222222222	71.56844444444444	71.65466666666667	43.24755555555556	71.60588888888888	66.04833333333333
CKSNAP-pseknc-GB	71.33622222222222	69.49677777777778	73.17877777777778	69.49677777777778	70.86322222222222	42.732	71.33777777777777	66.1418888888889

CKSNAP-NAC-GB	71.19833333333332	69.15633333333334	73.24111111111111	69.15633333333334	70.6241111111111	42.4473333333333	71.19888888888889	65.84555555555556
scpsednc-PseEIIP-SVM	71.08988888888888	68.0281111111111	74.15288888888888	68.0281111111111	70.16866666666667	42.2933333333333	71.09055555555557	66.08966666666667
CKSNAP-psednc-GB	71.00544444444444	70.73444444444445	71.27377777777777	70.73444444444445	70.89977777777777	42.043	71.00411111111111	65.66044444444444
psednc-scpsednc-LDA	70.89666666666668	70.39977777777777	71.393	70.39977777777777	70.80966666666667	41.8365555555556	70.89633333333333	65.336
CKSNAP-NCP-GB	70.84166666666667	69.83788888888888	71.84922222222222	69.83788888888888	70.58633333333334	41.76066666666665	70.84355555555555	65.51877777777777
CKSNAP-TNC-GB	70.69211111111112	69.12122222222223	72.26355555555556	69.12122222222223	70.25033333333334	41.4128888888889	70.69244444444445	65.31166666666667
CKSNAP-scpsednc-RF	70.54422222222223	70.048	71.041	70.048	70.41799999999999	41.1956666666667	70.54444444444444	65.47488888888888
CKSNAP-PseEIIP-GB	70.54222222222222	68.76077777777778	72.32433333333333	68.76077777777778	70.0111111111112	41.116	70.54266666666666	65.21611111111112
scpsednc-NAC-LDA	70.53522222222222	71.66155555555555	69.4058888888889	71.66155555555555	70.92333333333333	41.146	70.53388888888888	64.87566666666666
CKSNAP-PseEIIP-AB	70.40133333333333	69.32033333333334	71.48322222222222	69.32033333333334	70.13933333333334	40.8714444444445	70.40166666666667	65.13044444444444
CKSNAP-TNC-AB	70.40133333333333	69.32033333333334	71.48322222222222	69.32033333333334	70.13933333333334	40.8714444444445	70.40166666666667	65.13044444444444
CKSNAP-DNC-AB	69.93677777777778	68.68111111111111	71.19311111111112	68.68111111111111	69.641	39.9575555555556	69.93711111111111	64.823
CKSNAP-NAC-AB	69.93677777777778	68.68111111111111	71.19311111111112	68.68111111111111	69.641	39.9575555555556	69.93711111111111	64.823
psednc-DNC-LDA	69.90466666666667	68.18233333333335	71.6258888888889	68.18233333333335	69.39455555555554	39.883	69.90388888888889	64.59188888888889

ENAC-CKSNAP-GB	69.81244444444445	67.50311111111111	72.12444444444445	67.50311111111111	69.11066666666666	39.7018888888889	69.81366666666666	64.66844444444445
CKSNAP-psekn c-AB	69.81066666666668	68.35166666666666	71.26933333333334	68.35166666666666	69.37922222222223	39.6708888888889	69.81055555555555	64.63166666666666
DNC-scpsednc-SVM	69.69144444444444	67.74944444444445	71.63266666666667	67.74944444444445	69.197	39.4797777777778	69.691	64.732
scpsednc-NAC-SVM	69.66088888888889	65.49433333333333	73.82877777777779	65.49433333333333	68.4131111111111	39.6033333333333	69.66155555555555	65.113
binary-CKSNAP-GB	69.58522222222223	67.85477777777777	71.315	67.85477777777777	69.06511111111111	39.226	69.58488888888888	64.59377777777779
DNC-scpsednc-LDA	69.45788888888889	69.20577777777777	69.70844444444444	69.20577777777777	69.43133333333334	38.9545555555556	69.45711111111112	64.00622222222222
CKSNAP-psednc-AB	69.26266666666668	67.36955555555556	71.15355555555556	67.36955555555556	68.59811111111111	38.6046666666667	69.26144444444444	64.096
psednc-PseEIIP-LDA	69.08844444444445	66.49799999999999	71.67966666666666	66.49799999999999	68.31022222222222	38.2863333333333	69.08877777777778	64.019
psednc-TNC-LDA	69.08844444444445	66.49799999999999	71.67966666666666	66.49799999999999	68.31022222222222	38.2863333333333	69.08877777777778	64.019
EIIP-CKSNAP-GB	68.78744444444445	67.68144444444445	69.89277777777778	67.68144444444445	68.60255555555555	37.6975555555556	68.78722222222223	63.914777777777786
psednc-scpsednc-GB	68.72	69.10577777777777	68.33	69.10577777777777	68.87522222222222	37.50133333333335	68.7178888888889	63.45277777777778
scpsednc-psek nc-AB	68.63322222222223	68.48811111111111	68.77766666666668	68.48811111111111	68.632	37.2953333333333	68.63288888888889	63.67444444444445
scpsednc-NAC-GB	68.57400000000001	68.78255555555556	68.36322222222222	68.78255555555556	68.71655555555556	37.328111111111106	68.57288888888888	63.199999999999996
scpsednc-PseEIIP-LDA	68.52933333333334	67.86055555555555	69.19455555555555	67.86055555555555	68.29333333333334	37.140888888888895	68.52755555555557	63.083888888888886

scpsednc-TNC-LDA	68.52933333333334	67.86055555555555	69.19455555555555	67.86055555555555	68.29333333333334	37.14088888888895	68.52755555555557	63.083888888888886
CKSNAP-scpsednc-ET	68.44122222222222	66.54233333333333	70.342	66.54233333333333	67.95622222222222	36.98855555555556	68.442	63.87811111111111
CKSNAP-ANF-GB	68.42099999999999	66.81311111111111	70.02711111111111	66.81311111111111	67.997	36.91388888888889	68.42044444444444	63.519111111111116
psednc-PseEIIP-SVM	68.34322222222221	65.82866666666666	70.85888888888888	65.82866666666666	67.35111111111111	36.84711111111111	68.3438888888889	63.287333333333336
CKSNAP-NCP-AB	68.13466666666666	66.70566666666667	69.56633333333333	66.70566666666667	67.71811111111111	36.36066666666667	68.13588888888889	63.12833333333333
pseknc-NAC-LDA	67.70822222222222	65.29433333333333	70.12511111111111	65.29433333333333	66.92733333333334	35.53788888888889	67.70955555555555	62.93977777777778
CKSNAP-NAC-RF	67.3771111111111	65.77077777777778	68.98277777777778	65.77077777777778	66.78866666666666	34.85655555555556	67.37677777777778	62.70544444444445
EIIP-scpsednc-GB	67.28555555555556	67.40655555555556	67.15988888888889	67.40655555555556	67.44577777777778	34.67088888888889	67.28333333333333	62.105888888888884
EIIP-CKSNAP-AB	67.28244444444445	66.40866666666666	68.15533333333333	66.40866666666666	67.03944444444444	34.64066666666667	67.28188888888889	62.805
DNC-pseknc-LDA	67.18088888888889	66.50966666666666	67.85444444444444	66.50966666666666	67.06555555555556	34.476	67.18222222222222	62.53888888888889
scpsednc-NAC-AB	67.11166666666666	68.89844444444445	65.32255555555555	68.89844444444445	67.80577777777778	34.29088888888889	67.11044444444445	61.98144444444444
psednc-scpsednc-AB	67.07766666666666	68.72666666666666	65.42811111111111	68.72666666666666	67.674	34.20588888888889	67.07733333333334	61.912777777777784
scpsednc-TNC-SVM	67.02655555555556	64.3891111111111	69.66277777777778	64.3891111111111	66.17733333333334	34.16211111111111	67.02600000000001	62.55455555555555
CKSNAP-pseknc-RF	67.0168888888889	65.40355555555556	68.63277777777778	65.40355555555556	66.44188888888888	34.16166666666666	67.01822222222222	62.66044444444444
psednc-pseknc-GB	66.98511111111111	68.21766666666667	65.7508888888889	68.21766666666667	67.50477777777778	34.01733333333333	66.98433333333334	62.24788888888889

binary-scpsednc-GB	66.92233333333334	66.89777777777778	66.94455555555555	66.89777777777778	66.98488888888889	33.9242222222222	66.9211111111111	61.73477777777777
scpsednc-PseEIIP-GB	66.83244444444443	66.63344444444445	67.02955555555555	66.63344444444445	66.91333333333334	33.7925555555555	66.83155555555555	61.81955555555555
scpsednc-pseknc-RF	66.78533333333333	66.10355555555556	67.46711111111111	66.10355555555556	66.666	33.671	66.78544444444444	62.03822222222222
scpsednc-NCP-GB	66.7811111111111	67.65444444444444	65.90477777777778	67.65444444444444	67.27777777777777	33.64799999999996	66.77966666666667	61.87744444444445
DNC-scpsednc-GB	66.73355555555555	67.075	66.39111111111112	67.075	66.98911111111111	33.57477777777776	66.7331111111111	61.71977777777777
scpsednc-TNC-GB	66.63733333333333	66.72677777777778	66.54544444444444	66.72677777777778	66.82755555555555	33.4061111111111116	66.63622222222222	61.66655555555556
DNC-scpsednc-AB	66.55466666666666	67.14377777777777	65.96311111111112	67.14377777777777	66.84622222222222	33.16066666666664	66.55355555555555	61.531555555555556
CKSNAP-psekn c-LR	66.48633333333333	69.60666666666667	63.36577777777778	69.60666666666667	67.44022222222222	33.395	66.48622222222222	62.30633333333333
scpsednc-NAC-RF	66.33866666666667	66.66455555555555	66.01233333333333	66.66455555555555	66.46833333333333	32.79911111111111	66.33822222222221	61.42344444444445
CKSNAP-PseEIIP-LR	66.27466666666666	69.45833333333333	63.09055555555556	69.45833333333333	67.22644444444444	32.9728888888889	66.27455555555555	62.04122222222222
CKSNAP-psednc-LR	66.20833333333333	69.26966666666667	63.147222222222226	69.26966666666667	67.15533333333333	32.64755555555556	66.20844444444444	61.982666666666674
CKSNAP-TNC-LR	66.1531111111111	69.42777777777778	62.87833333333333	69.42777777777778	67.16811111111112	32.53200000000004	66.15299999999999	61.89544444444444
CKSNAP-DNC-RF	66.09522222222222	65.39155555555556	66.79933333333332	65.39155555555556	65.91455555555555	32.24277777777775	66.09533333333333	61.50544444444444
CKSNAP-psednc-RF	66.05344444444444	65.64977777777777	66.45466666666667	65.64977777777777	65.98988888888888	32.189	66.05233333333334	61.89511111111111
scpsednc-A	65.97133333333333	66.68666666666667	65.25355555555556	66.68666666666667	66.27566666666667	31.98366666666668	65.97	61.05588888888888

NF-GB								
binary-CKSNAP-AB	65.95266666666667	63.785888888888884	68.12166666666667	63.785888888888884	65.15566666666666	31.9507777777778	65.9538888888889	61.331
CKSNAP-scpsednc-LR	65.91244444444445	68.68877777777779	63.13677777777778	68.68877777777779	66.81555555555556	32.0033333333334	65.91266666666667	61.779222222222224
CKSNAP-NAC-LR	65.83233333333334	69.35288888888888	62.31122222222223	69.35288888888888	66.93	31.8965555555555	65.83211111111112	61.656
ENAC-CKSNAP-AB	65.79177777777778	62.82544444444444	68.75911111111111	62.82544444444444	64.71333333333334	31.710333333333338	65.79233333333333	61.48433333333334
CKSNAP-scpsednc-Bagging	65.78777777777778	57.708111111111116	73.87177777777778	57.708111111111116	62.64322222222222	32.0036666666667	65.7898888888889	61.45722222222222
CKSNAP-DNC-LR	65.76422222222223	68.97577777777778	62.55277777777778	68.97577777777778	66.83155555555555	31.583666666666666	65.76433333333334	61.63377777777777
CKSNAP-psednc-ET	65.75366666666667	62.249111111111105	69.25566666666667	62.249111111111105	64.51066666666667	31.633	65.75233333333333	61.46044444444445
ENAC-scpsednc-GB	65.73933333333333	64.28722222222223	67.19066666666667	64.28722222222223	65.32344444444445	31.566	65.73877777777778	60.81688888888888
scpsednc-psekn c-ET	65.61422222222222	64.21155555555555	67.01933333333334	64.21155555555555	65.24766666666667	31.3007777777778	65.61533333333334	61.47522222222223
CKSNAP-pseknc-ET	65.60544444444446	64.29622222222223	66.91411111111111	64.29622222222223	65.16000000000001	31.28011111111111	65.60499999999999	61.577333333333335
scpsednc-NAC-Bagging	65.42377777777777	59.800000000000004	71.04855555555555	59.800000000000004	63.17766666666666	31.06388888888886	65.42444444444443	60.80777777777777
psednc-pseknc-AB	65.34322222222222	66.34333333333333	64.34188888888889	66.34333333333333	65.73344444444444	30.715333333333334	65.34266666666666	60.650777777777776
CKSNAP-NAC-Bagging	65.16288888888889	56.75311111111111	73.58477777777779	56.75311111111111	61.768	30.79322222222223	65.16911111111112	60.912333333333336

scpsednc-PseEIIP-AB	65.15199999999999	65.93400000000001	64.368	65.93400000000001	65.54277777777777	30.33244444444448	65.15122222222222	60.427666666666674
scpsednc-TNC-AB	65.15199999999999	65.93400000000001	64.368	65.93400000000001	65.54277777777777	30.33244444444448	65.15122222222222	60.427666666666674
scpsednc-NAC-ET	64.94244444444445	64.77255555555556	65.11044444444445	64.77255555555556	65.00855555555556	29.9884444444444	64.94133333333333	60.72911111111111
TNC-pseknc-LDA	64.93955555555556	63.08877777777778	66.79377777777778	63.08877777777778	64.28633333333333	29.94288888888888	64.94133333333333	60.623999999999995
pseknc-PseEIIP-LDA	64.93955555555556	63.08877777777778	66.79377777777778	63.08877777777778	64.28633333333333	29.94288888888888	64.94133333333333	60.623999999999995
CKSNAP-NAC-ET	64.93833333333333	62.85788888888889	67.01822222222222	62.85788888888889	64.13244444444445	29.98511111111111	64.938	60.598666666666674
scpsednc-pseknc-Bagging	64.78655555555555	57.43922222222222	72.13633333333333	57.43922222222222	61.90855555555556	29.925111111111114	64.78777777777778	60.51611111111111
CKSNAP-ANF-AB	64.77733333333333	63.50322222222222	66.05288888888889	63.50322222222222	64.38011111111112	29.644111111111116	64.77799999999999	60.626666666666665
psednc-scpsednc-Bagging	64.74011111111112	59.27700000000001	70.20766666666667	59.27700000000001	62.69622222222222	29.75066666666667	64.74233333333333	60.30144444444444
psednc-scpsednc-ET	64.64311111111111	64.22755555555555	65.05644444444445	64.22755555555555	64.68755555555555	29.41066666666664	64.6418888888889	60.44833333333333
CKSNAP-DNC-Bagging	64.56133333333334	55.437777777777775	73.68811111111111	55.437777777777775	60.869	29.64277777777778	64.56288888888889	60.315777777777775
DNC-scpsednc-Bagging	64.49211111111111	58.01377777777778	70.97433333333333	58.01377777777778	61.995000000000005	29.2474444444444	64.49377777777778	60.08444444444444
CKSNAP-pseknc-Bagging	64.11588888888889	56.11011111111111	72.12888888888888	56.11011111111111	60.81144444444445	28.58966666666667	64.11944444444445	60.11255555555556
DNC-scpsednc-RF	64.05022222222222	64.55955555555555	63.541111111111114	64.55955555555555	64.25677777777778	28.15977777777776	64.05022222222222	59.56699999999999

CKSNAP-DNC-ET	63.64544444444444	61.35355555555556	65.93911111111112	61.35355555555556	62.66788888888888	27.352666666666668	63.64633333333333	59.720777777777776
scpsednc-ANF-AB	63.38699999999999	61.99533333333333	64.77455555555555	61.99533333333333	63.02411111111111	26.91288888888889	63.385000000000005	59.24544444444444
psednc-DNC-SVM	63.260777777777776	62.585111111111104	63.93622222222223	62.585111111111104	63.14522222222222	26.57388888888889	63.260777777777776	59.498555555555555
ENAC-psednc-GB	63.10933333333334	62.48255555555555	63.736555555555555	62.48255555555555	62.873333333333335	26.27277777777776	63.10955555555555	58.730777777777774
psednc-DNC-AB	63.028333333333336	64.61522222222223	61.437666666666665	64.61522222222223	63.69433333333333	26.088222222222225	63.026777777777774	58.763222222222225
CKSNAP-psednc-Bagging	62.854444444444454	57.16	68.55344444444444	57.16	60.45533333333333	25.87733333333333	62.85677777777778	58.80788888888888
scpsednc-NCP-AB	62.74155555555555	63.477666666666664	62.00266666666667	63.477666666666664	63.10788888888889	25.50733333333335	62.74011111111112	58.49555555555556
EIIP-scpsednc-AB	62.70633333333333	62.31644444444444	63.09466666666666	62.31644444444444	62.653777777777776	25.494222222222223	62.705555555555556	58.892222222222216
psednc-DNC-GB	62.64577777777778	64.77122222222222	60.51688888888889	64.77122222222222	63.43877777777777	25.348111111111113	62.644000000000005	58.404444444444444
CKSNAP-PseEIIP-RF	62.63077777777778	60.69244444444444	64.56766666666667	60.69244444444444	61.909666666666666	25.37166666666666	62.629999999999995	59.01188888888888
psednc-NAC-GB	62.57533333333333	65.8101111111111	59.336	65.8101111111111	63.85388888888888	25.22766666666664	62.57300000000001	58.34444444444445
psednc-NAC-AB	62.477000000000004	63.52011111111111	61.432444444444435	63.52011111111111	62.87144444444444	24.987111111111112	62.47622222222222	58.351888888888894
psednc-NCP-GB	62.309111111111115	64.00466666666667	60.61322222222222	64.00466666666667	63.010777777777776	24.639555555555557	62.30866666666667	58.3228888888889
ENAC-psekn c-GB	62.24633333333333	59.510666666666665	64.98633333333333	59.510666666666665	61.102444444444444	24.555888888888887	62.248555555555555	58.40266666666667
EIIP-psednc-GB	62.19566666666666	62.49433333333333	61.89677777777778	62.49433333333333	62.31488888888888	24.44777777777777	62.19555555555556	58.12533333333334
binary-psednc-GB	62.13166666666666	62.37466666666666	61.88788888888889	62.37466666666666	62.32866666666666	24.326555555555554	62.13133333333334	58.291111111111114

psednc-ANF-GB	62.10988888888889	61.31766666666667	62.90355555555556	61.31766666666667	61.760555555555555	24.252111111111113	62.110777777777784	58.242444444444445
binary-scpsednc-AB	62.05733333333333	61.45166666666666	62.66444444444445	61.45166666666666	61.848000000000006	24.14811111111111	62.05799999999999	58.07111111111111
CKSNAP-PseEIIP-Bagging	62.05188888888889	53.56100000000001	70.54733333333333	53.56100000000001	58.44866666666667	24.48055555555554	62.05422222222223	58.44022222222222
CKSNAP-TNC-RF	61.82877777777779	61.007444444444445	62.64788888888889	61.007444444444445	61.53555555555556	23.7087777777778	61.827555555555556	58.45877777777778
DNC-scpsednc-ET	61.670666666666676	61.17444444444445	62.16633333333333	61.17444444444445	61.72200000000001	23.413888888888888	61.67033333333333	58.50255555555556
psednc-pseknc-Bagging	61.659111111111116	55.91755555555555	67.40366666666667	55.91755555555555	59.24211111111111	23.516111111111112	61.66066666666667	58.196
scpsednc-pseknc-DT	61.60666666666667	61.58222222222222	61.62833333333333	61.58222222222222	61.61944444444445	23.230888888888888	61.605111111111114	57.58888888888888
scpsednc-NCP-Bagging	61.53611111111112	54.25822222222222	68.81788888888889	54.25822222222222	58.358333333333334	23.28733333333336	61.538	57.748222222222225
pseknc-NCP-GB	61.287222222222226	58.00444444444444	64.57333333333332	58.00444444444444	59.76477777777778	22.636444444444447	61.28888888888889	57.787222222222226
psednc-PseEIIP-GB	61.28033333333334	62.67533333333333	59.88288888888889	62.67533333333333	61.85788888888889	22.58777777777777	61.278999999999996	57.587777777777774
psekn c-ANF-GB	61.20411111111111	58.69544444444445	63.71422222222222	58.69544444444445	60.141	22.44022222222222	61.204888888888895	57.894777777777776
psednc-TNC-GB	61.18344444444444	62.331888888888884	60.03211111111111	62.331888888888884	61.65877777777777	22.391111111111112	61.182	57.51788888888888
EIIP-psek nc-GB	61.12033333333333	59.63422222222222	62.607777777777784	59.63422222222222	60.48844444444444	22.2986666666667	61.120999999999995	57.61066666666667
psednc-TNC-SVM	61.10966666666666	58.97344444444444	63.24411111111111	58.97344444444444	60.281	22.30733333333332	61.10877777777778	58.10466666666667
ENAC-scpsednc-Bagging	61.09144444444444	52.45655555555555	69.7328888888889	52.45655555555555	57.26888888888888	22.49322222222222	61.09466666666666	57.401333333333326

EIIP-CKSNAP-Bagging	61.008111111111106	51.016444444444446	71.00755555555556	51.016444444444446	56.537444444444446	22.418666666666667	61.01211111111112	57.50222222222222
scpsednc-ANF-Bagging	61.00055555555556	52.04988888888889	69.95844444444444	52.04988888888889	56.984	22.356444444444445	61.004111111111115	57.35322222222222
binary-scpsednc-Bagging	60.98055555555556	53.657555555555554	68.30566666666667	53.657555555555554	57.876777777777775	22.230666666666664	60.98155555555556	57.428
psednc-scpsednc-DT	60.97155555555556	60.99411111111111	60.95022222222222	60.99411111111111	60.953	21.962333333333333	60.97233333333333	57.06155555555555
DNC-pseknc-GB	60.80377777777778	59.43555555555557	62.17477777777778	59.43555555555557	60.19533333333334	21.645	60.80533333333334	57.52455555555556
psednc-pseknc-RF	60.63688888888888	60.78911111111111	60.48366666666667	60.78911111111111	60.654222222222224	21.288555555555558	60.63655555555556	57.55733333333333
psednc-PseEIIP-AB	60.61611111111111	62.439888888888895	58.78944444444445	62.439888888888895	61.31933333333333	21.29	60.61466666666667	56.87866666666667
psednc-TNC-AB	60.61611111111111	62.439888888888895	58.78944444444445	62.439888888888895	61.31933333333333	21.29	60.61466666666667	56.87866666666667
scpsednc-PseEIIP-Bagging	60.58033333333333	53.28333333333333	67.88144444444444	53.28333333333333	57.273111111111106	21.362333333333332	60.58233333333333	57.135555555555555
CKSNAP-NCP-Bagging	60.45099999999999	51.503	69.40433333333334	51.503	56.379666666666665	21.2106666666667	60.45355555555555	57.22611111111111
EIIP-scpsednc-Bagging	60.42622222222222	52.955666666666666	67.90055555555556	52.955666666666666	57.087666666666664	21.09911111111111	60.428222222222225	56.775999999999996
CKSNAP-psednc-DT	60.40677777777777	59.38000000000001	61.436666666666675	59.38000000000001	59.861555555555555	20.845777777777776	60.40822222222222	56.641888888888886
EIIP-psednc-AB	60.337666666666664	60.92377777777777	59.74888888888889	60.92377777777777	60.66711111111111	20.698222222222224	60.336333333333336	56.82522222222222
scpsednc-pseknc-NB	60.33233333333333	47.62211111111111	73.04755555555556	47.62211111111111	54.26266666666666	21.3417777777778	60.335111111111104	57.337333333333326

pseknc-NAC-GB	60.26555555555555	58.28088888888889	62.25622222222223	58.28088888888889	59.35466666666667	20.595	60.26844444444444	57.29322222222223
psednc-NAC-SVM	60.20455555555556	53.53655555555556	66.87644444444445	53.53655555555556	56.47188888888889	20.90811111111111	60.20655555555556	57.04855555555556
psednc-pseknc-DT	60.154333333333334	60.412777777777784	59.895999999999994	60.412777777777784	60.32277777777777	20.3367777777778	60.154222222222224	56.663333333333334
CKSNAP-TNC-Bagging	60.098111111111116	50.379555555555555	69.82233333333333	50.379555555555555	55.693333333333335	20.595222222222223	60.10111111111111	56.937333333333335
scpsednc-NAC-DT	59.971222222222224	59.91111111111112	60.03322222222222	59.91111111111112	59.925000000000004	19.9638888888889	59.97211111111111	56.19522222222222
CKSNAP-scpsednc-DT	59.95266666666666	60.82555555555555	59.07844444444444	60.82555555555555	60.281	19.945000000000004	59.95177777777778	56.425
scpsednc-TNC-Bagging	59.94366666666667	54.650777777777776	65.23922222222222	54.650777777777776	57.633111111111106	20.0322222222222	59.94477777777778	56.50922222222223
pseknc-PseEIIP-SVM	59.93144444444445	54.754111111111115	65.115	54.754111111111115	57.766333333333336	20.093777777777778	59.93455555555555	57.29833333333334
CKSNAP-NAC-DT	59.84433333333334	60.279333333333334	59.41166666666667	60.279333333333334	59.94633333333333	19.73311111111111	59.845555555555556	56.297555555555554
psednc-NCP-AB	59.83588888888889	60.12522222222222	59.54644444444445	60.12522222222222	59.93044444444445	19.697333333333333	59.836	56.096
psednc-DNC-DT	59.82677777777778	60.83244444444444	58.82311111111111	60.83244444444444	60.24555555555556	19.6722222222222	59.82788888888889	56.28755555555556
EIIP-psednc-Bagging	59.715888888888884	50.17733333333334	69.258	50.17733333333334	55.31955555555555	19.830444444444442	59.71777777777778	56.48455555555555
psednc-scpsednc-NB	59.70655555555556	48.92355555555556	70.49622222222223	48.92355555555556	54.88777777777778	19.9897777777778	59.70988888888889	56.61322222222222
binary-pseknc-GB	59.704888888888895	58.18311111111112	61.23044444444444	58.18311111111112	58.94800000000001	19.476666666666667	59.706888888888884	56.72088888888889
DNC-scpsednc-DT	59.601222222222226	59.215888888888884	59.984777777777786	59.215888888888884	59.379999999999995	19.2352222222222	59.60033333333334	56.07355555555555

DNC-pseknc-AB	59.55377777777778	58.18844444444445	60.92166666666666	58.18844444444445	58.89344444444444	19.1452222222222	59.55477777777779	56.669000000000004
EIIP-CKSNAP-RF	59.54211111111111	55.79088888888889	63.294111111111114	55.79088888888889	57.88033333333333	19.159888888888887	59.54244444444445	56.391222222222225
binary-psednc-AB	59.50544444444444	59.42788888888889	59.581999999999994	59.42788888888889	59.425222222222224	19.0788888888889	59.504888888888885	56.17477777777778
ENAC-psecknc-Bagging	59.42922222222223	48.324222222222225	70.54555555555555	48.324222222222225	54.278666666666666	19.442777777777778	59.434999999999995	56.44411111111111
scpsednc-NAC-NB	59.40411111111112	48.92955555555555	69.88355555555556	48.92955555555555	54.63722222222222	19.2751111111111112	59.406444444444446	56.23733333333333
CKSNAP-DNC-DT	59.38433333333334	59.18777777777777	59.58522222222223	59.18777777777777	59.18377777777778	18.836333333333336	59.38644444444445	56.001000000000005
psednc-NAC-Bagging	59.32888888888889	54.09444444444445	64.5648888888889	54.09444444444445	57.13777777777778	18.806111111111111	59.32966666666667	56.29022222222222
pseknc-NCP-Bagging	59.32344444444444	48.27366666666667	70.38366666666667	48.27366666666667	53.91122222222222	18.997111111111111	59.32866666666666	56.47533333333334
pseknc-NAC-AB	59.29155555555556	58.529555555555554	60.055	58.529555555555554	58.724999999999994	18.6468888888889	59.29233333333333	56.67122222222222
CKSNAP-pseknc-DT	59.281333333333336	57.96911111111111	60.596666666666664	57.96911111111111	58.85433333333333	18.6202222222222	59.28277777777779	56.172555555555554
EIIP-scpsednc-RF	59.206888888888884	55.33166666666667	63.083222222222226	55.33166666666667	57.626555555555555	18.504888888888885	59.20744444444444	56.42066666666667
psednc-NCP-Bagging	59.1358888888889	50.137	68.13855555555556	50.137	55.047444444444444	18.599888888888888	59.13766666666667	55.85666666666666
DNC-scpsednc-NB	58.97855555555556	48.56422222222222	69.398	48.56422222222222	54.23011111111111	18.416	58.98111111111112	55.965888888888884
EIIP-scpsednc-DT	58.63477777777777	59.891666666666666	57.37766666666667	59.891666666666666	59.18	17.293777777777777	58.63477777777777	55.54188888888889
DNC-pseknc-Bagging	58.60433333333333	50.973222222222226	66.24666666666667	50.973222222222226	54.747	17.342666666666666	58.61	56.22233333333334

psednc-DNC-Bagging	58.58233333333333	53.208222222222226	63.958999999999996	53.208222222222226	56.08788888888889	17.264666666666667	58.58355555555555	55.731
scpsednc-PseEIIP-RF	58.54288888888888	59.596222222222224	57.486000000000004	59.596222222222224	59.14311111111111	17.1322222222222	58.541111111111114	56.14866666666667
binary-CKSNAP-DT	58.41511111111111	58.55822222222222	58.27333333333334	58.55822222222222	58.248555555555555	16.929111111111112	58.41577777777778	55.28577777777778
ENAC-CKSNAP-Bagging	58.28477777777778	46.916333333333334	69.6621111111111	46.916333333333334	52.66744444444444	16.88111111111111	58.28922222222223	55.745444444444445
pseknc-ANF-AB	58.270999999999994	56.01122222222222	60.53222222222222	56.01122222222222	57.219222222222214	16.578777777777777	58.271666666666675	55.715
scpsednc-TNC-DT	58.22	56.922555555555554	59.517222222222216	56.922555555555554	57.750888888888895	16.554111111111112	58.219888888888896	55.37822222222222
CKSNAP-PseEIIP-ET	58.21333333333333	57.86955555555556	58.556000000000004	57.86955555555556	58.24366666666666	16.422222222222224	58.212777777777774	56.34988888888889
pseknc-NCP-AB	58.148222222222216	55.53955555555555	60.75911111111111	55.53955555555555	57.00944444444445	16.32011111111111	58.149333333333324	55.46988888888889
binary-CKSNAP-Bagging	58.094888888888896	48.68355555555556	67.51455555555555	48.68355555555556	53.586444444444446	16.606444444444445	58.099111111111114	55.873000000000005
CKSNAP-ANF-Bagging	58.05055555555556	46.90455555555556	69.20822222222222	46.90455555555556	52.62455555555555	16.5788888888889	58.05644444444445	55.227444444444444
ENAC-psednc-Bagging	58.00977777777777	48.714666666666666	67.306	48.714666666666666	53.15555555555555	16.288	58.01044444444445	55.23844444444444
EIIP-psednc-DT	57.97822222222222	57.49688888888889	58.45777777777778	57.49688888888889	57.845888888888894	16.014666666666667	57.977444444444444	54.90222222222222
ENAC-pseknc-AB	57.95422222222222	56.083888888888886	59.827222222222225	56.083888888888886	57.22144444444445	15.948666666666668	57.955555555555556	55.401444444444444
pseknc-PseEIIP-GB	57.94033333333333	56.44766666666666	59.43655555555555	56.44766666666666	57.086555555555556	15.914666666666667	57.94200000000001	55.891888888888886

scpsednc-TNC-RF	57.92699999999999	57.42711111111112	58.42533333333333	57.42711111111112	57.71333333333333	15.866888888888887	57.92633333333333	55.650666666666666
psednc-NAC-RF	57.83377777777778	60.43933333333334	55.224555555555554	60.43933333333334	59.02844444444444	15.705	57.831888888888884	55.37477777777778
scpsednc-NCP-DT	57.83111111111111	57.06955555555555	58.59522222222222	57.06955555555555	57.62244444444444	15.720666666666666	57.83222222222222	55.04577777777777
ENAC-psednc-AB	57.81166666666666	57.46766666666666	58.154333333333334	57.46766666666666	57.681555555555555	15.637	57.811	54.80277777777778
ENAC-scpsednc-RF	57.79544444444444	54.108111111111114	61.483222222222224	54.108111111111114	56.150000000000006	15.69822222222222	57.79577777777778	54.94766666666666
pseknc-NAC-Bagging	57.784000000000006	51.03311111111111	64.54377777777778	51.03311111111111	54.568444444444445	15.71388888888889	57.788444444444444	55.57922222222222
binary-psednc-Bagging	57.7561111111111	48.43577777777777	67.0791111111111	48.43577777777777	53.29944444444445	15.820333333333334	57.75722222222222	54.99988888888889
binary-scpsednc-DT	57.73388888888889	57.79322222222223	57.67355555555556	57.79322222222223	57.720555555555556	15.566111111111111	57.733333333333334	54.64533333333333
binary-pseknc-Bagging	57.70055555555555	44.79377777777778	70.61755555555555	44.79377777777778	51.26611111111111	15.973666666666666	57.70544444444445	55.65577777777778
CKSNAP-ANF-RF	57.65344444444444	54.66866666666667	60.639111111111106	54.66866666666667	56.42377777777778	15.363444444444447	57.65366666666667	55.36666666666667
TNC-pseknc-GB	57.57844444444444	55.82911111111111	59.331	55.82911111111111	56.65222222222222	15.17777777777777	57.58011111111111	55.599444444444444
scpsednc-PseEIIP-DT	57.397555555555556	56.809555555555555	57.98877777777778	56.809555555555555	57.154555555555554	14.825111111111111	57.39911111111111	54.70666666666667
ENAC-psednc-DT	57.31077777777778	59.86477777777778	54.758	59.86477777777778	58.37533333333334	14.652333333333335	57.31144444444445	54.45622222222222
psednc-pseknc-ET	57.30388888888889	56.95011111111111	57.65677777777778	56.95011111111111	57.236666666666665	14.6158888888888889	57.303555555555555	55.85077777777778
CKSNAP-NCP-DT	57.25788888888889	57.730000000000004	56.78577777777778	57.730000000000004	57.45288888888889	14.53111111111111	57.25788888888889	54.71977777777778

psednc-NAC-NB	57.24555555555556	45.67633333333333	68.8201111111111	45.67633333333333	51.68855555555555	15.128777777777778	57.248333333333335	54.81588888888889
binary-scpsednc-RF	57.22466666666667	55.98066666666667	58.46811111111111	55.98066666666667	56.734	14.483	57.224333333333334	54.50588888888889
CKSNAP-PseEIIP-DT	57.203222222222216	55.422222222222224	58.98511111111111	55.422222222222224	56.40877777777777	14.429555555555554	57.20366666666666	54.62611111111111
psednc-ANF-Bagging	57.17077777777778	46.799	67.54855555555555	46.799	52.17677777777778	14.676111111111112	57.17377777777777	54.70588888888889
CKSNAP-scpsednc-NB	57.02711111111111	44.75966666666667	69.30188888888888	44.75966666666667	50.96077777777778	14.686555555555556	57.03088888888889	54.629666666666665
TNC-pseknc-AB	56.95722222222222	54.26777777777778	59.648222222222216	54.26777777777778	55.63377777777778	13.919222222222222	56.958	55.272
pseknc-PseEIIP-AB	56.95722222222222	54.26777777777778	59.648222222222216	54.26777777777778	55.63377777777778	13.919222222222222	56.958	55.272
ENAC-CKSNAP-DT	56.94233333333333	56.28111111111111	57.599999999999994	56.28111111111111	56.559333333333335	13.969666666666667	56.940666666666665	54.28933333333333
EIIP-pseknc-Bagging	56.73688888888889	46.129888888888885	67.34988888888888	46.129888888888885	51.35322222222222	13.759666666666668	56.739888888888885	54.67444444444445
binary-CKSNAP-RF	56.68822222222222	53.23522222222223	60.144999999999996	53.23522222222223	55.08022222222222	13.425111111111111	56.690111111111115	54.34811111111111
ENAC-scpsednc-DT	56.647333333333336	54.216	59.078222222222216	54.216	55.61077777777778	13.36111111111111	56.64722222222222	54.126333333333335
pseknc-NAC-SVM	56.64244444444444	45.772	67.52155555555555	45.772	50.801	13.726888888888888	56.64677777777778	55.17322222222222
pseknc-NAC-DT	56.620888888888885	58.026333333333326	55.21644444444444	58.026333333333326	57.24377777777777	13.281444444444444	56.62122222222222	54.39311111111111
pseknc-ANF-Bagging	56.61055555555556	46.27433333333333	66.95611111111111	46.27433333333333	51.501555555555555	13.499444444444444	56.61533333333333	54.48122222222222
CKSNAP-N	56.58922222222222	53.449777777777776	59.72666666666666	53.449777777777776	55.230111111111114	13.245444444444445	56.58822222222222	54.205333333333336

CP-RF								
pseknc-NCP-DT	56.495555555555555	54.62488888888889	58.36855555555556	54.62488888888889	55.644777777777776	13.021999999999998	56.49688888888889	53.97377777777778
CKSNAP-pseknc-NB	56.398555555555554	42.211888888888886	70.59422222222221	42.211888888888886	49.047555555555554	13.459	56.403222222222226	54.44088888888889
pseknc-NAC-NB	56.39544444444445	40.14488888888889	72.659	40.14488888888889	47.54533333333333	13.404555555555554	56.402	54.71377777777778
pseknc-PseEIIP-Bagging	56.35988888888889	47.144666666666666	65.58433333333333	47.144666666666666	51.717999999999996	12.918222222222221	56.364444444444445	54.92466666666667
CKSNAP-ANF-LDA	56.32588888888889	54.11066666666667	58.54366666666667	54.11066666666667	55.29255555555556	12.671222222222221	56.32711111111111	54.14588888888889
EIIP-CKSNAP-LDA	56.27022222222222	56.016444444444446	56.52333333333333	56.016444444444446	56.07622222222222	12.563111111111112	56.27	53.88911111111111
binary-pseknc-AB	56.266666666666666	56.190777777777775	56.34211111111111	56.190777777777775	56.153666666666666	12.548	56.266444444444446	54.388333333333335
scpsednc-ANF-DT	56.26422222222222	53.56122222222222	58.974222222222224	53.56122222222222	54.977333333333334	12.572222222222223	56.26777777777777	53.800222222222224
scpsednc-PseEIIP-NB	56.209666666666664	45.03944444444445	67.38333333333334	45.03944444444445	50.72588888888889	12.984111111111112	56.211444444444446	54.19533333333333
scpsednc-TNC-NB	56.209666666666664	45.03944444444445	67.38333333333334	45.03944444444445	50.72588888888889	12.984111111111112	56.211444444444446	54.19533333333333
DNC-pseknc-DT	56.169555555555554	56.446222222222225	55.89344444444444	56.446222222222225	56.245777777777775	12.354111111111111	56.169999999999995	54.026444444444444
ENAC-pseknc-DT	56.11066666666667	52.827444444444446	59.39944444444445	52.827444444444446	54.63655555555555	12.281444444444444	56.11322222222222	53.943333333333335
ENAC-psednc-RF	56.053666666666665	49.97088888888889	62.13944444444444	49.97088888888889	53.20655555555556	12.231666666666667	56.05522222222223	53.75322222222222
binary-psednc-DT	56.050777777777775	55.55833333333333	56.54622222222222	55.55833333333333	55.742222222222225	12.120777777777779	56.052	53.68977777777778
psednc-PseEIIP-B	56.01877777777778	51.022	61.013888888888886	51.022	53.595333333333336	12.139333333333333	56.018	54.147444444444446

agging								
CKSNAP-psednc-NB	55.974222222222224	42.041111111111114	69.91633333333333	42.041111111111114	48.83144444444444	12.643888888888888	55.97866666666667	53.92111111111112
scpsednc-NCP-RF	55.870777777777775	53.10733333333334	58.633	53.10733333333334	54.71044444444445	11.827444444444446	55.870111111111115	53.68233333333333
psednc-NCP-DT	55.864555555555555	55.85155555555556	55.88077777777778	55.85155555555556	55.876222222222225	11.730555555555556	55.86611111111111	53.763555555555556
psednc-DNC-RF	55.781222222222226	57.83033333333333	53.72866666666667	57.83033333333333	56.63066666666666	11.608666666666666	55.779555555555554	54.092999999999996
CKSNAP-ANF-DT	55.736444444444444	55.093444444444444	56.385	55.093444444444444	55.405	11.497555555555556	55.73911111111111	53.5111111111111
psednc-DNC-NB	55.69855555555555	44.154666666666664	67.24766666666667	44.154666666666664	49.824777777777776	11.874555555555554	55.70111111111111	53.75111111111111
DNC-pseknc-NB	55.67122222222222	39.400333333333336	71.95266666666666	39.400333333333336	46.73911111111111	11.963888888888889	55.67644444444444	54.138555555555556
ENAC-CKSNAP-RF	55.657222222222224	50.891444444444446	60.42888888888889	50.891444444444446	53.40555555555555	11.394222222222222	55.66	53.68088888888889
psednc-TNC-Bagging	55.56111111111111	49.78666666666666	61.34066666666667	49.78666666666666	52.62588888888889	11.227222222222222	55.56366666666666	53.76233333333333
psednc-TNC-DT	55.51233333333333	57.90299999999999	53.11844444444444	57.90299999999999	56.68388888888889	11.047333333333334	55.510777777777776	53.54533333333333
EIIP-pseknc-DT	55.46277777777778	52.873444444444445	58.050777777777775	52.873444444444445	54.199111111111115	10.935444444444446	55.46233333333333	53.37444444444444
pseknc-NAC-RF	55.35044444444444	55.16777777777778	55.53644444444444	55.16777777777778	55.20944444444444	10.722555555555555	55.352333333333334	54.373111111111115
scpsednc-ANF-ET	55.21311111111111	51.62811111111111	58.80044444444444	51.62811111111111	53.763555555555556	10.5768888888889	55.214444444444446	53.86655555555555
scpsednc-pseknc-LR	55.17744444444444	72.63322222222222	37.721777777777774	72.63322222222222	60.68377777777778	12.163888888888888	55.17744444444444	53.001222222222225
psednc-ANF-DT	54.93866666666666	55.126000000000005	54.75366666666667	55.126000000000005	54.943555555555555	9.907444444444444	54.93988888888889	52.88488888888889

psednc-ANF-RF	54.914888888888896	51.11222222222222	58.717444444444446	51.11222222222222	53.12888888888889	9.916444444444444	54.91477777777778	53.19411111111111
psednc-PseEIIP-DT	54.86077777777778	57.68555555555555	52.032222222222224	57.68555555555555	56.17	9.715666666666667	54.858777777777775	52.91544444444445
CKSNAP-NAC-NB	54.76566666666666	40.56188888888889	68.97800000000001	40.56188888888889	47.26244444444444	10.179555555555556	54.77	53.169333333333334
psednc-scpsednc-LR	54.699222222222225	72.65511111111111	36.74411111111111	72.65511111111111	60.47544444444444	10.916444444444444	54.69966666666667	52.705000000000005
ENAC-ANF-GB	54.65288888888889	50.33266666666667	58.974	50.33266666666667	52.54511111111111	9.425666666666666	54.653333333333336	52.93111111111111
pseknc-ANF-RF	54.565888888888885	51.24666666666667	57.885666666666665	51.24666666666667	52.95922222222222	9.303666666666667	54.566	53.218222222222224
TNC-pseknc-DT	54.55277777777778	54.07577777777778	55.02477777777778	54.07577777777778	54.31977777777777	9.124	54.55033333333333	52.93677777777778
TNC-pseknc-Bagging	54.541666666666664	45.80188888888889	63.289222222222214	45.80188888888889	49.95088888888889	9.140777777777778	54.54555555555555	53.76788888888889
CKSNAP-pseknc-KNN	54.51177777777778	24.026444444444444	85.0218888888889	24.026444444444444	34.26533333333333	11.110777777777777	54.52388888888889	53.18955555555556
CKSNAP-psednc-KNN	54.46088888888889	24.221999999999998	84.72444444444444	24.221999999999998	34.425777777777775	10.942222222222222	54.473	53.15133333333333
CKSNAP-PseEIIP-KNN	54.336666666666666	23.957	84.74055555555555	23.957	34.11266666666667	10.66	54.34866666666667	53.06922222222222
EIIP-psednc-RF	54.29355555555556	52.00088888888889	56.58566666666667	52.00088888888889	53.16588888888889	8.623444444444445	54.29333333333333	52.45144444444444
CKSNAP-DNC-NB	54.20411111111111	40.129222222222225	68.28688888888888	40.129222222222225	46.67488888888889	8.978666666666665	54.20811111111111	52.76833333333334
ENAC-CKSNAP-ET	54.19255555555556	46.883111111111106	61.507444444444445	46.883111111111106	50.50911111111111	8.573888888888888	54.19533333333333	52.51188888888888
ENAC-pseknc-RF	54.19233333333333	49.45166666666667	58.936	49.45166666666667	51.919666666666664	8.457444444444445	54.19377777777778	52.83166666666667

DNC-psekn c-SVM	54.139666666 66667	50.516111111 11111	57.769000000 000005	50.516111111 11111	52.451111111 11111	8.3271111111111 12	54.142444444 44444	53.989777777 77778
scpse dnc-P seEIIP -LR	54.110666666 66667	72.379111111 11111	35.842999999 999996	72.379111111 11111	59.996333333 33333	9.743	54.111	52.367333333 333335
ENAC -TNC-SVM	54.025444444 444446	52.993888888 88889	55.059666666 666665	52.993888888 88889	53.703666666 66667	8.128	54.027	52.759777777 77778
ENAC -psed nc-SV M	53.999777777 77778	52.968333333 333334	55.034111111 111116	52.968333333 333334	53.677777777 77778	8.07677777777 7776	54.001333333 333335	52.742111111 11111
ENAC -CKS NAP-SVM	53.988111111 11112	52.884444444 44444	55.094666666 66666	52.884444444 44444	53.631333333 33334	8.0541111111111 12	53.989666666 66667	52.733222222 222224
ENAC -psek nc-SV M	53.984666666 66666	52.968333333 333334	55.003777777 77778	52.968333333 333334	53.669888888 88889	8.04655555555 5555	53.986222222 22222	52.733888888 88889
ENAC -DNC-SVM	53.975333333 33334	52.919333333 333334	55.034111111 111116	52.919333333 333334	53.641111111 11111	8.02866666666 6668	53.976888888 888894	52.724666666 666664
ENAC -NAC-SVM	53.969444444 44445	52.968333333 333334	54.973555555 555556	52.968333333 333334	53.662	8.01633333333 3332	53.971000000 000004	52.725777777 77778
ENAC -PseE IIP-SV M	53.969444444 44445	52.968333333 333334	54.973555555 555556	52.968333333 333334	53.662	8.01633333333 3332	53.971000000 000004	52.725777777 77778
ENAC -scpse dnc-S VM	53.936888888 88889	52.893777777 77778	54.982888888 88889	52.893777777 77778	53.607555555 55556	7.95177777777 7778	53.938444444 44445	52.698111111 11111
psekn c-Pse EIIP-D T	53.895666666 66666	53.991111111 11111	53.797222222 222224	53.991111111 11111	53.970777777 777776	7.81122222222 2223	53.894	52.580666666 666666
ENAC -TNC-GB	53.774555555 55556	51.214666666 666666	56.336222222 222226	51.214666666 666666	52.504222222 222225	7.5871111111111 12	53.775444444 444446	52.154
ENAC -NAC-GB	53.699444444 444445	50.151444444 444444	57.250111111 11111	50.151444444 444444	51.966888888 88889	7.44188888888 8889	53.700888888 88889	52.115555555 55556
EIIP-E NAC-SVM	53.623777777 777775	52.635999999 999996	54.614222222 222224	52.635999999 999996	53.300222222 222224	7.3051111111111 11	53.625111111 11111	52.469333333 33333
scpse dnc-T NC-L R	53.596777777 777774	71.429222222 22222	35.763444444 444445	71.429222222 22222	59.354888888 88889	8.8881111111111 1	53.596333333 333334	52.062555555 555555

binary-pseknc-RF	53.547333333333334	46.937	60.16377777777778	46.937	50.06522222222222	7.115555555555556	53.550222222222224	52.35366666666667
EIIP-pseknc-RF	53.37766666666667	49.289	57.46811111111111	49.289	51.26277777777778	6.758222222222222	53.37844444444445	52.44811111111111
CKSNAP-NAC-KNN	53.366888888888894	22.18188888888889	84.577	22.18188888888889	31.837888888888887	8.306	53.379222222222225	52.25388888888889
TNC-pseknc-SVM	53.30833333333333	48.618222222222215	58.00333333333333	48.618222222222215	50.964333333333336	6.6400000000000015	53.311	53.68633333333334
CKSNAP-scpsednc-KNN	53.24477777777778	22.67677777777778	83.83655555555555	22.67677777777778	32.37588888888889	7.882000000000001	53.25677777777778	52.26811111111111
CKSNAP-PseEIIP-NB	53.24044444444444	39.456888888888884	67.02911111111112	39.456888888888884	45.68222222222222	6.966555555555555	53.243	52.22188888888889
CKSNAP-TNC-NB	53.24044444444444	39.456888888888884	67.02911111111112	39.456888888888884	45.68222222222222	6.966555555555555	53.243	52.22188888888889
CKSNAP-ANF-ET	53.18577777777777	49.54644444444445	56.827333333333335	49.54644444444445	51.538222222222224	6.508555555555556	53.18711111111111	52.45988888888889
psednc-ANF-ET	53.06688888888888	48.88966666666667	57.24444444444445	48.88966666666667	51.04022222222222	6.209222222222222	53.06711111111111	51.96666666666667
ENAC-scpsednc-ET	53.06633333333333	47.346222222222224	58.79022222222222	47.346222222222224	50.199555555555555	6.198666666666666	53.06811111111111	51.745
scpsednc-NAC-LR	53.05866666666667	69.45244444444444	36.660888888888884	69.45244444444444	58.5281111111111	7.221444444444444	53.056777777777775	51.748999999999995
CKSNAP-TNC-KNN	53.02055555555555	22.375777777777778	83.69011111111111	22.375777777777778	31.911222222222225	7.303000000000001	53.033	52.202333333333335
ENAC-ANF-SVM	53.004999999999995	51.48533333333333	54.528	51.48533333333333	52.42477777777778	6.085	53.00655555555555	52.156444444444446
ENAC-NCP-GB	52.93588888888889	49.236999999999995	56.638000000000005	49.236999999999995	51.11288888888889	5.935666666666666	52.937444444444445	51.68711111111111
EIIP-scpsednc-LDA	52.92533333333333	54.81944444444444	51.026555555555554	54.81944444444444	53.86622222222222	5.846555555555557	52.922888888888885	52.01811111111111

ENAC-PseEIIP-GB	52.92455555555556	50.22311111111111	55.62744444444444	50.22311111111111	51.583666666666666	5.885666666666666	52.92533333333333	51.67944444444444
DNC-scpsednc-LR	52.830333333333336	70.021	35.63755555555556	70.021	58.48111111111112	6.83255555555555	52.82933333333333	51.647999999999996
EIIP-CKSNAP-ET	52.76766666666666	48.44711111111111	57.092	48.44711111111111	50.57533333333333	5.543333333333333	52.76933333333333	51.93388888888889
TNC-pseknc-NB	52.703111111111106	39.123111111111115	66.28455555555554	39.123111111111115	44.87544444444445	5.709222222222222	52.70388888888888	52.42388888888889
pseknc-PseEIIP-NB	52.703111111111106	39.123111111111115	66.28455555555554	39.123111111111115	44.87544444444445	5.709222222222222	52.70388888888888	52.42388888888889
psekn c-NCP-RF	52.63044444444444	48.83566666666666	56.42911111111111	48.83566666666666	50.775666666666666	5.293666666666667	52.632333333333335	51.82566666666666
scpsednc-NCP-LDA	52.617888888888885	51.705333333333336	53.531777777777776	51.705333333333336	52.18411111111111	5.249111111111111	52.61877777777778	51.55811111111112
ENAC-DNC-GB	52.607777777777784	48.806555555555555	56.41177777777778	48.806555555555555	50.664	5.244777777777778	52.609111111111105	51.47566666666667
scpsednc-PseEIIP-ET	52.519444444444446	52.10655555555556	52.93322222222222	52.10655555555556	52.57344444444445	5.045666666666665	52.519777777777776	52.94777777777777
scpsednc-TNC-ET	52.504333333333335	51.91711111111111	53.09022222222222	51.91711111111111	52.30877777777778	5.010444444444444	52.50366666666667	52.83733333333333
NCP-ANF-Bagging	52.486000000000004	38.74133333333334	66.23511111111111	38.74133333333334	44.85122222222222	5.19144444444445	52.48822222222222	51.41155555555556
CKSNAP-DNC-KNN	52.468111111111114	22.243000000000002	82.71711111111111	22.243000000000002	31.586444444444446	5.893888888888888	52.48	51.82233333333333
binary-CKSNAP-ET	52.17644444444445	49.10877777777778	55.24677777777778	49.10877777777778	50.64055555555555	4.375111111111111	52.17766666666667	51.373
binary-scpsednc-ET	52.11055555555556	47.55555555555556	56.66388888888889	47.55555555555556	49.730777777777774	4.240888888888889	52.10966666666666	51.30444444444444
psednc-PseEIIP-NB	52.07822222222222	42.113	62.041888888888884	42.113	46.40188888888889	4.472333333333334	52.077555555555556	51.56366666666666

psednc-TNC-NB	52.07822222222222	42.113	62.041888888888884	42.113	46.40188888888889	4.472333333333334	52.077555555555556	51.56366666666666
binary-NAC-DT	52.038222222222224	53.40788888888889	50.66855555555556	53.40788888888889	52.577666666666666	4.122555555555555	52.038111111111114	51.18011111111111
ENAC-DNC-Bagging	51.97666666666667	37.01533333333333	66.95044444444444	37.01533333333333	43.489333333333335	4.247222222222223	51.983111111111114	51.230333333333334
CKSNAP-NCP-LDA	51.96088888888889	52.01511111111111	51.909	52.01511111111111	51.949222222222225	3.921777777777778	51.96211111111111	51.175
EIIP-psednc-LDA	51.94866666666667	53.092444444444446	50.80522222222223	53.092444444444446	52.45222222222222	3.90277777777777	51.94888888888889	51.16588888888889
EIIP-pseknc-LDA	51.800444444444445	51.85655555555556	51.74444444444444	51.85655555555556	51.69177777777778	3.623333333333334	51.800444444444445	51.35422222222222
binary-psednc-ET	51.77388888888889	50.04911111111111	53.49922222222222	50.04911111111111	50.788777777777774	3.5556666666666663	51.774	51.16155555555556
ENAC-pseknc-ET	51.71655555555556	43.71566666666667	59.72344444444444	43.71566666666667	47.47211111111111	3.496111111111111	51.71955555555556	51.12244444444444
EIIP-scpsednc-ET	51.67744444444444	46.58811111111111	56.769111111111116	46.58811111111111	49.11	3.410777777777778	51.678444444444445	51.105222222222224
EIIP-ENAC-GB	51.65022222222222	49.15166666666667	54.150888888888886	49.15166666666667	50.339	3.306111111111106	51.65122222222222	51.01877777777778
ENAC-ANF-RF	51.65011111111111	45.65711111111111	57.64833333333334	45.65711111111111	48.578	3.389	51.65277777777778	51.098
EIIP-ANF-GB	51.623555555555555	47.436	55.818333333333335	47.436	49.510777777777776	3.3064444444444443	51.62733333333333	51.00511111111111
binary-ANF-DT	51.616888888888894	49.80444444444444	53.43266666666667	49.80444444444444	50.65855555555556	3.252111111111111	51.61866666666666	51.03788888888889
binary-DNC-DT	51.61533333333333	54.14033333333333	49.092777777777776	54.14033333333333	52.63444444444444	3.280777777777778	51.61644444444445	50.955666666666666
NCP-ANF-RF	51.59144444444444	44.410333333333334	58.77722222222222	44.410333333333334	47.72966666666667	3.2929999999999997	51.593777777777774	51.07044444444445
ENAC-NCP-Bagging	51.589555555555556	39.705000000000005	63.480777777777774	39.705000000000005	45.02455555555556	3.279777777777778	51.59288888888889	50.92344444444445

NAC-ANF-GB	51.56022222222222	47.61577777777778	55.507777777777775	47.61577777777778	49.53866666666667	3.155333333333333	51.56166666666667	50.971666666666664
DNC-pseknc-RF	51.54177777777778	51.51844444444444	51.56411111111111	51.51844444444444	51.516333333333336	3.1089999999999995	51.541444444444444	52.54188888888889
scpsednc-ANF-LDA	51.53622222222222	50.89666666666667	52.178333333333335	50.89666666666667	51.18211111111111	3.0803333333333334	51.53766666666667	51.58422222222222
binary-NAC-Bagging	51.5	40.583555555555556	62.425000000000004	40.583555555555556	45.54055555555556	3.088	51.504222222222225	50.934888888888885
ENAC-NAC-ET	51.47022222222222	45.71811111111111	57.227444444444444	45.71811111111111	48.39255555555556	2.995	51.47266666666667	50.87133333333333
ENAC-NCP-AB	51.462666666666664	51.44677777777778	51.478	51.44677777777778	51.36288888888889	2.9205555555555556	51.46244444444444	50.83377777777778
psednc-PseEIIP-LR	51.461555555555556	76.90644444444445	26.015333333333334	76.90644444444445	59.98155555555556	3.2093333333333334	51.46066666666667	50.80455555555555
ENAC-CKSNAP-LDA	51.461222222222226	50.58166666666667	52.342999999999996	50.58166666666667	50.973	2.9218888888888888	51.46233333333333	51.03633333333333
EIIP-ENAC-AB	51.45877777777778	49.81788888888889	53.09988888888889	49.81788888888889	50.57444444444444	2.9346666666666668	51.459	50.79377777777778
NAC-ANF-RF	51.43677777777778	47.400999999999996	55.474444444444444	47.400999999999996	49.34477777777778	2.9884444444444442	51.437777777777775	50.90155555555556
NCP-ANF-GB	51.385666666666665	47.17144444444445	55.60422222222222	47.17144444444445	48.96433333333333	2.7775555555555553	51.38777777777778	50.818999999999996
psefnc-ANF-ET	51.38422222222222	45.86033333333333	56.90944444444445	45.86033333333333	48.528888888888886	2.913444444444445	51.38488888888889	51.50611111111111
EIIP-ENAC-ET	51.36077777777778	43.57622222222222	59.150000000000006	43.57622222222222	47.18344444444445	2.756111111111116	51.363	50.83155555555555
binary-pseknc-ET	51.352333333333334	47.81155555555556	54.89688888888889	47.81155555555556	49.46955555555556	2.694666666666667	51.35433333333333	50.86477777777778
EIIP-CKSNAP-SVM	51.25555555555556	52.60444444444445	49.903555555555556	52.60444444444445	51.96788888888889	2.4972222222222222	51.25388888888889	51.03688888888889
psednc-pseknc-LR	51.24911111111111	76.43555555555555	26.060111111111112	76.43555555555555	59.74688888888889	2.678888888888889	51.24766666666667	50.711555555555556

DNC-ANF-AB	51.24044444444444	46.12422222222222	56.35988888888889	46.12422222222222	48.34788888888889	2.4813333333333333	51.242	50.93733333333333
ENAC-binary-Bagging	51.238555555555556	37.89366666666667	64.59266666666666	37.89366666666667	43.690333333333335	2.6102222222222222	51.243	50.70366666666667
DNC-ANF-DT	51.18322222222222	49.164	53.202555555555556	49.164	50.103	2.3716666666666666	51.18333333333333	50.687555555555555
ENAC-binary-GB	51.159444444444446	48.28322222222222	54.03677777777778	48.28322222222222	49.70455555555556	2.3357777777777775	51.16	50.659666666666666
ENAC-binary-AB	51.11366666666667	50.11088888888889	52.11411111111111	50.11088888888889	50.60122222222222	2.227111111111113	51.11244444444444	50.75511111111111
PseEIIP-ANF-RF	51.11011111111111	47.423	54.800111111111114	47.423	49.22222222222222	2.3036666666666665	51.11188888888889	50.882777777777775
ENAC-PseEIIP-Bagging	51.06066666666666	38.56488888888889	63.56699999999999	38.56488888888889	43.87877777777778	2.2386666666666667	51.066111111111105	50.65888888888889
ENAC-NCP-RF	51.053777777777775	44.56211111111111	57.55211111111112	44.56211111111111	47.59433333333333	2.1422222222222222	51.056999999999995	50.67433333333334
binary-scpsednc-LDA	51.05088888888889	51.22777777777778	50.87722222222222	51.22777777777778	51.144	2.1153333333333335	51.05266666666667	50.69311111111111
ENAC-psednc-ET	51.03111111111111	44.16888888888889	57.89788888888889	44.16888888888889	47.405111111111104	2.0828888888888892	51.03344444444444	50.693000000000005
ENAC-TNC-RF	51.020111111111106	43.61911111111111	58.42466666666667	43.61911111111111	46.995666666666665	2.0854444444444447	51.022	50.66177777777778
binary-CKSNAP-LDA	51.016555555555556	51.093333333333334	50.942	51.093333333333334	51.016222222222225	2.0487777777777776	51.01766666666666	50.78211111111111
EIIP-NCP-KNN	51.007777777777775	28.190777777777775	73.84144444444445	28.190777777777775	36.36377777777778	2.139	51.016	50.69177777777778
EIIP-ANF-DT	51.004444444444445	49.879	52.13455555555556	49.879	50.409111111111116	2.0208888888888889	51.00655555555555	50.605555555555554
ENAC-DNC-RF	50.925555555555555	45.70944444444444	56.144777777777776	45.70944444444444	48.16422222222222	1.8924444444444444	50.927	50.525888888888886
TNC-NCP-KNN	50.90222222222222	28.24388888888889	73.576	28.24388888888889	36.37444444444444	1.899	50.91	50.687555555555555

DNC-ANF-Bagging	50.88944444444444	38.02155555555556	63.763	38.02155555555556	43.61355555555556	1.92777777777776	50.89211111111111	50.61577777777777
binary-ANF-AB	50.88077777777778	48.995222222222225	52.76933333333333	48.995222222222225	49.94755555555556	1.76599999999996	50.882222222222225	50.79644444444445
CKSNAP-NCP-KNN	50.86355555555556	28.05966666666667	73.68277777777777	28.05966666666667	36.19544444444445	1.8047777777778	50.87144444444445	50.67411111111111
DNC-ANF-ET	50.86311111111111	46.19866666666667	55.52722222222222	46.19866666666667	48.513444444444445	1.83744444444447	50.86277777777778	50.912
pseknc-NCP-KNN	50.849999999999994	28.017777777777777	73.69755555555555	28.017777777777777	36.15822222222222	1.78022222222224	50.857777777777784	50.664
NCP-NAC-DT	50.84688888888889	49.59155555555556	52.10966666666666	49.59155555555556	50.178111111111114	1.73433333333335	50.85066666666667	50.56777777777778
PseEIIP-NCP-KNN	50.800444444444445	28.24388888888889	73.37233333333333	28.24388888888889	36.331888888888884	1.67733333333333	50.80811111111112	50.644666666666666
psednc-ANF-LDA	50.79266666666667	50.141666666666666	51.44488888888889	50.141666666666666	50.358444444444444	1.5866666666667	50.79322222222223	50.70444444444444
EIIP-ANF-Bagging	50.74611111111111	37.260444444444445	64.24066666666666	37.260444444444445	43.08533333333334	1.58977777777777	50.75077777777778	50.48855555555556
NCP-NAC-KNN	50.745777777777775	28.03411111111111	73.47266666666667	28.03411111111111	36.12466666666667	1.5446666666667	50.75366666666667	50.620555555555555
pseknc-NAC-ET	50.74022222222222	48.60922222222222	52.87488888888889	48.60922222222222	49.593777777777774	1.45788888888888	50.74211111111111	52.25011111111111
binary-psednc-RF	50.73444444444445	45.04855555555556	56.42933333333333	45.04855555555556	47.65777777777778	1.48055555555556	50.739000000000004	50.586888888888886
psednc-NCP-KNN	50.708666666666666	27.919666666666664	73.51288888888888	27.919666666666664	36.00633333333334	1.45500000000003	50.71644444444444	50.60644444444444
binary-ANF-RF	50.659666666666666	45.342333333333336	55.97833333333333	45.342333333333336	47.83422222222222	1.32077777777774	50.660222222222224	50.760111111111115
scpsednc-NCP-KNN	50.65477777777778	28.001333333333335	73.32366666666667	28.001333333333335	36.05244444444444	1.33555555555558	50.66255555555556	50.589
ENAC-PseEIIP-RF	50.64555555555555	47.44522222222222	53.85166666666667	47.44522222222222	48.943333333333335	1.28888888888888	50.648555555555554	50.40633333333333

ENAC-ANF-DT	50.638888888888886	49.24311111111111	52.032666666666664	49.24311111111111	49.849999999999994	1.2666666666666666	50.63777777777778	50.45044444444444
ENAC-PseEIIP-ET	50.635333333333335	44.60333333333333	56.67088888888889	44.60333333333333	47.433	1.2836666666666667	50.63711111111111	50.55322222222222
ENAC-PseEIIP-AB	50.629555555555555	50.33122222222222	50.92633333333333	50.33122222222222	50.42755555555556	1.2568888888888887	50.62866666666667	50.378
ENAC-TNC-AB	50.629555555555555	50.33122222222222	50.92633333333333	50.33122222222222	50.42755555555556	1.2568888888888887	50.62866666666667	50.378
DNC-NCP-KNN	50.62444444444444	27.996444444444442	73.26777777777778	27.996444444444442	36.03377777777777	1.2643333333333338	50.632222222222225	50.574
psednc-NCP-RF	50.61266666666667	47.61022222222223	53.617333333333335	47.61022222222223	49.20044444444444	1.3164444444444443	50.614000000000004	50.67422222222222
DNC-ANF-RF	50.61066666666667	45.48744444444444	55.733222222222224	45.48744444444444	47.983222222222224	1.3008888888888888	50.61033333333333	50.562777777777775
EIIP-ANF-RF	50.59888888888889	44.76866666666667	56.431000000000004	44.76866666666667	47.596444444444444	1.3876666666666668	50.599777777777774	50.68311111111112
binary-ANF-ET	50.550222222222224	48.343666666666664	52.762	48.343666666666664	49.37377777777778	1.1143333333333334	50.55277777777778	50.39911111111111
binary-ANF-Bagging	50.53677777777778	37.410333333333334	63.67144444444445	37.410333333333334	42.977555555555554	1.2184444444444444	50.54066666666667	50.43
pseknc-PseEIIP-LR	50.52111111111111	78.32222222222222	22.715333333333334	78.32222222222222	59.901888888888884	1.1927777777777777	50.51855555555556	50.30244444444444
CKSNAP-NCP-ET	50.515888888888895	45.150333333333336	55.88644444444444	45.150333333333336	47.67533333333333	1.047666666666667	50.51822222222222	50.33488888888889
NAC-ANF-AB	50.50333333333333	47.656555555555556	53.355	47.656555555555556	48.96211111111111	1.002444444444444	50.50566666666666	50.580999999999996
binary-DNC-AB	50.50244444444444	50.281555555555556	50.72188888888889	50.281555555555556	50.27077777777778	1.0018888888888886	50.50177777777778	50.547333333333334
binary-DNC-Bagging	50.47577777777778	37.83722222222222	63.12188888888889	37.83722222222222	43.251888888888885	0.9974444444444442	50.47944444444445	50.370666666666665
ENAC-scpsednc-LDA	50.422222222222224	51.751666666666665	49.093333333333334	51.751666666666665	51.09711111111111	0.8494444444444444	50.42266666666667	50.461777777777776

NAC-ANF-DT	50.40011111111111	47.452555555555556	53.351888888888894	47.452555555555556	48.83522222222222	0.8035555555555557	50.40222222222222	50.33522222222222
ENAC-DNC-ET	50.37733333333333	43.17566666666667	57.583111111111116	43.17566666666667	46.41888888888889	0.7405555555555556	50.379222222222225	50.36355555555556
scpsednc-NCP-ET	50.33288888888889	47.69277777777778	52.973555555555556	47.69277777777778	48.93677777777778	0.6562222222222222	50.33311111111111	50.28788888888889
ENAC-binary-ET	50.327777777777776	46.757777777777775	53.903	46.757777777777775	48.467555555555556	0.6629999999999998	50.330333333333336	50.275555555555556
binary-ANF-GB	50.32355555555555	45.08344444444444	55.566	45.08344444444444	47.51877777777778	0.648111111111111	50.324777777777776	50.35788888888889
binary-NAC-GB	50.291777777777774	48.66744444444444	51.91855555555556	48.66744444444444	49.51166666666667	0.5951111111111113	50.293	50.32111111111111
EIIP-binary-DT	50.24888888888889	50.39377777777778	50.10188888888889	50.39377777777778	50.31622222222222	0.4976666666666667	50.24788888888889	50.166666666666664
ENAC-ANF-AB	50.24722222222223	48.19855555555556	52.29833333333333	48.19855555555556	49.13466666666667	0.5083333333333333	50.248444444444445	50.181444444444445
ENAC-TNC-ET	50.23911111111111	44.01533333333334	56.468222222222224	44.01533333333334	46.84444444444445	0.5193333333333333	50.241888888888894	50.23222222222223
TNC-ANF-DT	50.22544444444444	47.34166666666667	53.11188888888889	47.34166666666667	48.65122222222222	0.4473333333333335	50.22688888888889	50.257555555555555
ENAC-NCP-DT	50.19533333333334	52.29222222222222	48.10077777777778	52.29222222222222	51.10955555555555	0.4125555555555555	50.196555555555555	50.18933333333334
binary-ANF-LDA	50.17044444444444	50.607	49.73522222222223	50.607	50.38433333333333	0.344222222222222204	50.17122222222222	50.14966666666666
EIIP-DNC-DT	50.138333333333335	48.43788888888889	51.84011111111111	48.43788888888889	49.25577777777778	0.298888888888887	50.138888888888886	50.18033333333333
CKSNAP-ANF-NB	50.12822222222222	41.745333333333335	58.510111111111115	41.745333333333335	43.650666666666666	0.3056666666666667	50.12755555555555	50.19311111111111
ENAC-NAC-RF	50.126333333333335	45.032666666666664	55.22511111111111	45.032666666666664	47.446333333333335	0.3121111111111111	50.12888888888889	50.22622222222222
EIIP-NAC-Bagging	50.12444444444444	36.583	63.67044444444444	36.583	42.20811111111111	0.288888888888864	50.12677777777778	50.474555555555554
binary-PseEIIP-DT	50.114888888888885	49.443777777777775	50.78788888888889	49.443777777777775	49.71	0.235333333333333348	50.11588888888889	50.150000000000006

DNC-ANF-GB	50.10933333333333	47.10633333333333	53.117333333333335	47.10633333333333	48.392222222222216	0.22144444444444467	50.11177777777778	50.25466666666667
psednc-NCP-LDA	50.10277777777778	50.714111111111116	49.49155555555555	50.714111111111116	50.32511111111111	0.20500000000000001	50.103	50.12566666666666
binary-DNC-ET	50.10055555555555	46.263999999999996	53.93888888888889	46.263999999999996	47.98444444444445	0.18044444444444444	50.101444444444446	50.157222222222224
NCP-ANF-KNN	50.00533333333333	26.15377777777778	73.87533333333334	26.15377777777778	34.30144444444444	0.03311111111111015	50.01433333333333	50.18033333333333
psekn c-NAC-LR	50.00177777777778	70.76966666666667	29.22855555555556	70.76966666666667	57.29066666666667	0.18222222222222217	49.998999999999995	50.074888888888886
EIIP-DNC-AB	49.98133333333333	49.87266666666667	50.086888888888886	49.87266666666667	49.87711111111111	-0.039333333333333345	49.979888888888894	50.056555555555555
EIIP-ANF-LDA	49.96522222222222	50.37577777777778	49.553333333333335	50.37577777777778	50.21333333333334	-0.057666666666666894	49.964444444444446	50.166333333333334
DNC-psekn c-LR	49.93922222222222	73.71233333333333	26.16188888888889	73.71233333333333	58.19488888888889	-0.05511111111111086	49.937	50.031555555555556
psednc-TNC-LR	49.92755555555556	75.2328888888889	24.61922222222222	75.2328888888889	58.66100000000001	-0.13922222222222225	49.92611111111111	50.0
NCP-ANF-LDA	49.90711111111111	50.28388888888889	49.53133333333333	50.28388888888889	50.08288888888889	-0.17966666666666675	49.90777777777778	50.05433333333333
ENAC-TNC-Bagging	49.894777777777776	37.24422222222222	62.55955555555555	37.24422222222222	42.401555555555554	-0.1886666666666668	49.901777777777774	50.041777777777774
NCP-ANF-ET	49.87411111111111	43.70522222222222	56.047555555555554	43.70522222222222	46.57866666666666	-0.22944444444444434	49.876555555555555	50.07977777777778
EIIP-NCP-GB	49.87288888888889	49.10711111111111	50.63811111111111	49.10711111111111	49.425222222222224	-0.22277777777777766	49.87266666666667	50.022999999999996
EIIP-NAC-DT	49.85033333333334	50.459	49.24344444444445	50.459	50.117888888888885	-299	49.85122222222222	49.97244444444445
EIIP-binary-ET	49.84722222222222	48.162333333333336	51.534888888888894	48.162333333333336	48.92944444444444	-0.30844444444444445	49.84866666666667	50.00911111111111
binary-DNC-GB	49.80122222222222	47.63477777777778	51.969	47.63477777777778	48.66	-0.40322222222222215	49.80188888888889	50.00788888888889
binary-NCP-DT	49.79677777777778	51.24688888888889	48.34788888888889	51.24688888888889	50.462555555555554	-0.40177777777777773	49.797444444444444	49.96888888888889

EIIP-ANF-AB	49.789888888888896	48.254777777777775	51.32277777777777	48.254777777777775	48.98122222222222	-0.42511111111111133	49.788555555555554	49.992777777777775
TNC-ANF-Bagging	49.781444444444446	37.10666666666666	62.46455555555556	37.10666666666666	42.41555555555556	-0.37533333333333335	49.78577777777778	50.14011111111111
NAC-ANF-Bagging	49.77611111111111	37.089	62.47233333333333	37.089	42.43011111111111	-0.4749999999999999	49.78066666666667	50.01166666666667
ENAC-NAC-DT	49.77	51.06744444444444	48.47533333333334	51.06744444444444	50.34111111111111	-0.468888888888888897	49.77122222222223	49.96511111111111
ENAC-binary-KNN	49.74911111111111	21.05177777777778	78.47111111111111	21.05177777777778	29.405888888888892	-0.5385555555555555	49.76155555555555	50.06988888888889
binary-NCP-Bagging	49.74533333333333	36.132444444444445	63.36711111111111	36.132444444444445	41.71666666666667	-0.5462222222222223	49.74966666666667	50.056000000000004
binary-TNC-Bagging	49.74144444444445	37.71633333333333	61.77477777777777	37.71633333333333	42.36288888888888	-0.6923333333333331	49.745333333333335	50.077555555555556
ENAC-NAC-Bagging	49.736444444444444	37.78088888888889	61.702	37.78088888888889	42.81044444444444	-0.4342222222222222	49.74144444444445	50.07533333333333
scpsednc-ANF-NB	49.72022222222222	42.13933333333333	57.29877777777777	42.13933333333333	43.66766666666667	-0.4874444444444444	49.718777777777774	50.013666666666666
TNC-ANF-RF	49.70455555555556	43.722	55.69177777777778	43.722	46.562444444444445	-0.5115555555555555	49.706888888888884	50.095
NAC-ANF-ET	49.70377777777778	44.929111111111105	54.47888888888889	44.929111111111105	47.173	-0.5398888888888892	49.704	50.08855555555556
binary-NCP-GB	49.67722222222223	50.15166666666667	49.199777777777776	50.15166666666667	49.86	-0.6562222222222222	49.675555555555555	49.921888888888894
PseEIIP-ANF-DT	49.66866666666667	47.03677777777778	52.303666666666665	47.03677777777778	48.24711111111111	-0.6604444444444443	49.67055555555556	50.01433333333333
binary-NAC-RF	49.641888888888886	46.346666666666664	52.93833333333333	46.346666666666664	47.94	-707	49.64244444444445	49.91077777777778
ENAC-CKSNAP-NB	49.61955555555556	40.73188888888889	58.498222222222225	40.73188888888889	41.510777777777776	-0.8798888888888887	49.614999999999995	49.931999999999995
EIIP-ENAC-	49.61811111111111	38.989444444444445	60.25266666666667	38.989444444444445	43.62088888888889	-0.7692222222222223	49.621111111111105	49.858333333333334

Bagging								
psednc-NAC-LR	49.58222222222222	71.08677777777778	28.072666666666667	71.08677777777778	57.186777777777785	-0.8783333333333334	49.57944444444445	49.830333333333336
binary-TNC-DT	49.56955555555555	49.42355555555556	49.718333333333334	49.42355555555556	49.477666666666664	-0.8534444444444444	49.570888888888895	49.901444444444444
EIIP-NCP-AB	49.55711111111111	51.04633333333334	48.065666666666665	51.04633333333334	50.15988888888889	-0.9023333333333332	49.55611111111111	49.83488888888889
psednc-DNC-LR	49.55611111111111	73.48277777777778	25.625444444444444	73.48277777777778	57.82044444444445	-1.2427777777777775	49.554111111111105	49.81488888888889
DNC-NCP-RF	49.54544444444444	46.69722222222222	52.39688888888889	46.69722222222222	48.01255555555556	-0.9103333333333333	49.54711111111111	49.885999999999996
EIIP-NAC-RF	49.50688888888889	46.20644444444444	52.806666666666665	46.20644444444444	47.732777777777784	-0.9883333333333333	49.50644444444444	49.88422222222223
binary-NAC-KNN	49.48588888888889	21.254	77.74966666666666	21.254	28.931333333333335	-1.0385555555555555	49.501999999999995	49.99666666666667
EIIP-binary-KNN	49.48422222222222	21.325000000000003	77.67455555555556	21.325000000000003	29.074888888888886	-1.117111111111111	49.49977777777778	50.08855555555556
binary-psednc-KNN	49.468444444444444	21.37066666666667	77.59788888888889	21.37066666666667	29.074888888888886	-1.0065555555555554	49.48422222222222	50.01188888888888
NCP-NAC-GB	49.44766666666666	50.039	48.855111111111114	50.039	49.63022222222222	-1.1168888888888889	49.44711111111111	49.80233333333334
binary-NAC-LDA	49.44188888888888	51.26844444444444	47.61622222222222	51.26844444444444	50.33588888888889	-1.1308888888888888	49.44244444444444	49.80811111111112
binary-NCP-LDA	49.43855555555555	50.955666666666666	47.922	50.955666666666666	50.15977777777778	-1.1313333333333333	49.43888888888889	49.83422222222222
binary-ANF-NB	49.40744444444445	55.52811111111111	43.27477777777778	55.52811111111111	48.858666666666664	-1.0628888888888889	49.40133333333333	49.86366666666666
ENAC-ANF-NB	49.38666666666667	39.958	58.806000000000004	39.958	40.98777777777778	-1.3338888888888889	49.38188888888889	49.82933333333333
NCP-NAC-Bagging	49.38488888888889	34.858666666666664	63.925111111111114	34.858666666666664	40.66688888888889	-1.3285555555555557	49.391888888888886	49.766222222222225
binary-PseEIIP-KNN	49.38055555555556	20.871111111111112	77.92222222222222	20.871111111111112	28.446	-1.4054444444444445	49.39677777777778	49.992333333333335

binary-NAC-ET	49.379888888888885	44.12722222222222	54.635777777777776	44.12722222222222	46.605222222222224	-1.2286666666666666	49.38155555555555	50.00833333333333
binary-CKSNAP-NB	49.37555555555556	56.47588888888889	42.262888888888895	56.47588888888889	49.20877777777778	-1.2238888888888889	49.36933333333334	49.778333333333336
NCP-ANF-AB	49.36833333333333	47.791000000000004	50.946	47.791000000000004	48.49344444444444	-1.2725555555555554	49.36855555555556	49.71911111111111
TNC-pseknc-LR	49.36611111111111	74.81844444444444	23.909444444444446	74.81844444444444	58.14122222222222	0.218555555555561	49.364000000000004	49.73877777777778
binary-DNC-KNN	49.33133333333333	20.805	77.88955555555555	20.805	28.370333333333335	-1.5176666666666667	49.34744444444445	49.97844444444444
EIIP-ANF-ET	49.31688888888888	42.650777777777776	55.98911111111111	42.650777777777776	45.71433333333333	-1.3166666666666667	49.31988888888889	49.80166666666666
binary-scpsednc-KNN	49.30622222222222	20.867	77.77744444444446	20.867	28.432333333333336	-1.5356666666666667	49.32211111111111	49.986111111111114
EIIP-ENAC-DT	49.30266666666667	50.08211111111111	48.522333333333336	50.08211111111111	49.59444444444445	-1.4072222222222222	49.30233333333334	49.784555555555556
ENAC-scpsednc-NB	49.29611111111111	40.05455555555555	58.52788888888889	40.05455555555555	40.89788888888889	-1.5566666666666666	49.29122222222222	49.768
binary-pseknc-LDA	49.288111111111114	50.12566666666666	48.452333333333335	50.12566666666666	49.691	-1.4260000000000002	49.289	49.74455555555556
binary-pseknc-KNN	49.28077777777778	20.982	77.61166666666666	20.982	28.562888888888885	-1.5656666666666668	49.29666666666667	49.97266666666667
PseEIIP-ANF-ET	49.26522222222222	44.34722222222222	54.18444444444444	44.34722222222222	46.81055555555556	-1.2813333333333334	49.26566666666667	50.10333333333333
DNC-NCP-DT	49.260333333333335	50.40266666666667	48.12	50.40266666666667	49.73211111111111	-1.4720000000000002	49.26122222222222	49.717666666666666
binary-CKSNAP-KNN	49.26022222222222	20.89333333333333	77.65855555555555	20.89333333333333	28.489555555555555	-1.5911111111111111	49.275999999999996	49.967444444444446
binary-NCP-AB	49.23122222222222	49.647555555555556	48.81344444444445	49.647555555555556	49.42422222222223	-1.546	49.230444444444444	49.67033333333333
EIIP-binary-LDA	49.211888888888886	50.465333333333334	47.96133333333333	50.465333333333334	49.844	-1.5788888888888889	49.21333333333334	49.65411111111111

EIIP-NCP-Bagging	49.190888888888885	38.751555555555555	59.63777777777778	38.751555555555555	43.224555555555554	-1.6705555555555556	49.19488888888889	49.68188888888889
psednc-ANF-NB	49.18333333333333	41.21455555555556	57.14844444444444	41.21455555555556	42.74933333333333	-1.5997777777777773	49.181444444444445	49.785555555555554
binary-NCP-ET	49.17911111111111	44.69088888888889	53.67344444444444	44.69088888888889	46.742111111111114	-1.6477777777777778	49.18222222222222	49.721444444444444
binary-NCP-KNN	49.17277777777778	24.580444444444442	73.78500000000001	24.580444444444442	32.53622222222222	-1.825888888888889	49.18255555555555	49.768
ENAC-psek nc-NB	49.166777777777774	40.230222222222224	58.093444444444444	40.230222222222224	40.974444444444444	-1.8446666666666667	49.16188888888889	49.702777777777776
ENAC-psednc-LDA	49.15266666666667	49.87611111111111	48.43055555555556	49.87611111111111	49.46011111111111	-1.6962222222222225	49.153444444444446	49.702666666666666
EIIP-ENAC-NB	49.150999999999996	39.95111111111111	58.33877777777778	39.95111111111111	40.77433333333333	-1.9274444444444445	49.144888888888886	49.70222222222222
EIIP-NCP-DT	49.147111111111116	50.31311111111111	47.97877777777778	50.31311111111111	49.54211111111111	-1.7214444444444448	49.146	49.69811111111111
ENAC-psednc-NB	49.14433333333333	40.10744444444444	58.17155555555556	40.10744444444444	40.846000000000004	-1.8845555555555553	49.139333333333326	49.684333333333335
ENAC-NAC-NB	49.13911111111111	40.18911111111111	58.07922222222222	40.18911111111111	40.86611111111111	-1.9161111111111113	49.13411111111111	49.66844444444444
DNC-PseEIIP-LR	49.090444444444444	73.69344444444444	24.48311111111111	73.69344444444444	57.614111111111114	-0.5393333333333332	49.08822222222222	49.599999999999994
binary-NAC-AB	49.06755555555556	48.031222222222226	50.10322222222222	48.031222222222226	48.422444444444444	-1.8725555555555558	49.06733333333333	49.91455555555555
PseEIIP-ANF-GB	49.05888888888889	45.409333333333336	52.71244444444444	45.409333333333336	47.090444444444444	-1.8856666666666664	49.061	49.69888888888889
EIIP-psek nc-ET	49.05066666666667	46.59288888888889	51.513000000000005	46.59288888888889	47.74433333333334	-1.9008888888888889	49.05288888888889	49.82522222222222
NCP-NAC-LDA	49.02055555555555	49.85444444444445	48.18655555555555	49.85444444444445	49.398555555555554	-1.963888888888889	49.02066666666666	49.629777777777775
TNC-ANF-GB	49.01622222222222	45.095	52.94211111111111	45.095	46.90344444444444	-1.9686666666666666	49.01855555555555	49.70144444444444
psekn c-ANF-NB	49.013888888888886	41.36233333333333	56.66266666666667	41.36233333333333	42.79911111111111	-1.9305555555555551	49.01222222222222	49.76433333333333

ENAC-DNC-NB	48.99688888888889	39.96688888888889	58.017222222222216	39.96688888888889	40.68933333333334	-2.1987777777777778	48.991888888888894	49.611444444444444
ENAC-NCP-NB	48.992333333333335	44.69455555555555	53.276777777777774	44.69455555555555	43.940666666666665	-2.3253333333333335	48.98577777777777	49.59511111111111
ENAC-PseEIIP-NB	48.992222222222225	39.890888888888895	58.083888888888886	39.890888888888895	40.68944444444444	-2.1741111111111113	48.98733333333333	49.599
ENAC-TNC-NB	48.992222222222225	39.890888888888895	58.083888888888886	39.890888888888895	40.68944444444444	-2.1741111111111113	48.98733333333333	49.599
PseEIIP-NAC-LR	48.949444444444445	70.65788888888889	27.235777777777777	70.65788888888889	56.69555555555556	-2.4395555555555557	48.946666666666665	49.534111111111116
NCP-NAC-AB	48.94877777777778	48.62755555555556	49.273666666666664	48.62755555555556	48.730444444444444	-2.1055555555555556	48.95044444444444	49.559555555555555
ENAC-DNC-DT	48.946333333333335	50.55555555555556	47.33933333333333	50.55555555555556	49.70233333333333	-2.1095555555555556	48.94733333333333	49.61655555555555
ENAC-binary-NB	48.93977777777778	55.56855555555555	42.29822222222222	55.56855555555555	48.562777777777775	-2.0838888888888887	48.93344444444445	49.554
EIIP-ANF-NB	48.925222222222224	44.22533333333334	53.61466666666667	44.22533333333334	44.27688888888889	-2.2424444444444447	48.91988888888889	49.64911111111111
ENAC-NCP-KNN	48.92433333333334	19.350555555555555	78.52066666666667	19.350555555555555	27.426000000000002	-2.6244444444444444	48.93555555555556	49.86177777777778
binary-ANF-KNN	48.906555555555556	21.256999999999998	76.58433333333333	21.256999999999998	29.017000000000003	-2.533	48.92066666666667	49.823
ENAC-psekn c-LDA	48.86322222222222	50.08711111111111	47.64044444444445	50.08711111111111	49.492333333333335	-2.277111111111111	48.86377777777778	49.77
EIIP-NAC-AB	48.855222222222224	48.416777777777774	49.29511111111111	48.416777777777774	48.61811111111111	-2.3068888888888889	48.855888888888884	49.523888888888884
binary-scpsednc-NB	48.848444444444446	56.333999999999996	41.35	56.333999999999996	48.91766666666667	-2.3231111111111113	48.842	49.525444444444446
ENAC-binary-RF	48.763333333333335	44.88644444444444	52.647	44.88644444444444	46.62566666666666	-2.4882222222222222	48.766666666666666	49.50044444444445
DNC-NAC-LR	48.73744444444444	67.66333333333334	29.806333333333335	67.66333333333334	55.779	-3.1357777777777778	48.73488888888889	49.422444444444444
TNC-NAC-LR	48.73244444444444	69.15755555555556	28.30211111111111	69.15755555555556	56.176	-3.1757777777777778	48.72966666666667	49.42666666666667

ENAC-NCP-LDA	48.71888888888889	48.879333333333335	48.559888888888885	48.879333333333335	48.79411111111111	-2.5686666666666667	48.71955555555556	49.46133333333333
ENAC-TNC-DT	48.70366666666667	49.29877777777778	48.11077777777778	49.29877777777778	49.022444444444446	-2.5952222222222222	48.70466666666667	49.46577777777778
DNC-ANF-NB	48.681555555555555	40.92133333333334	56.437777777777775	40.92133333333334	42.304111111111105	-2.6393333333333333	48.67955555555555	49.552
NAC-ANF-NB	48.67444444444445	40.92133333333334	56.42355555555556	40.92133333333334	42.29711111111111	-2.6424444444444444	48.672444444444444	49.54611111111111
ENAC-NCP-ET	48.65533333333334	41.647777777777776	55.665	41.647777777777776	44.754333333333335	-2.7087777777777777	48.65633333333333	49.480222222222224
CKSNAP-ANF-SVM	48.63066666666666	46.396	50.86911111111111	46.396	47.510777777777776	-2.7226666666666666	48.632666666666665	49.553
EIIP-scpsednc-NB	48.54955555555556	47.38	49.70733333333333	47.38	44.663111111111114	-3.1268888888888889	48.54333333333333	49.37777777777777
EIIP-DNC-Bagging	48.52611111111111	35.85622222222222	61.20533333333333	35.85622222222222	40.95488888888889	-3.05	48.53066666666667	49.33055555555555
NCP-ANF-NB	48.525444444444446	48.99933333333334	48.03888888888889	48.99933333333334	45.98777777777778	-3.2433333333333336	48.51888888888889	49.422
CKSNAP-NCP-NB	48.50677777777778	48.79088888888889	48.20977777777778	48.79088888888889	45.741	-3.2840000000000003	48.50022222222222	49.39011111111111
EIIP-binary-NB	48.501444444444445	54.00077777777778	42.989555555555555	54.00077777777778	47.190444444444445	-2.978111111111111	48.495222222222225	49.35122222222222
DNC-NCP-LDA	48.49888888888889	49.91211111111111	47.08533333333334	49.91211111111111	49.12822222222222	-3.0047777777777778	48.498555555555555	49.37122222222222
PseEIIP-NAC-SVM	48.47633333333333	25.327	71.63866666666667	25.327	32.132333333333335	-3.1167777777777778	48.48266666666667	49.54922222222222
PseEIIP-NCP-DT	48.46988888888889	49.434666666666665	47.50611111111111	49.434666666666665	48.964888888888886	-3.0633333333333335	48.47044444444444	49.301
EIIP-NAC-GB	48.45466666666667	49.22777777777778	47.681000000000004	49.22777777777778	48.845444444444446	-3.0957777777777777	48.45433333333333	49.330999999999996
TNC-ANF-ET	48.44433333333333	43.52044444444444	53.36922222222222	43.52044444444444	45.80144444444444	-3.0202222222222224	48.44477777777778	49.519111111111116

EIIP-pseknc-NB	48.43877777777778	46.89777777777778	49.967555555555556	46.89777777777778	44.24955555555555	-3.3937777777778	48.43244444444444	49.33822222222222
scpsednc-NCP-NB	48.42711111111111	48.31177777777778	48.52944444444445	48.31177777777778	45.31066666666666	-3.4923333333333	48.42055555555556	49.31888888888889
NCP-NAC-RF	48.42333333333333	44.19111111111111	52.65877777777777	44.19111111111111	46.16455555555555	-3.1484444444444445	48.42477777777778	49.32588888888889
binary-psednc-LDA	48.42077777777777	49.491	47.35133333333333	49.491	48.94188888888888	-3.1627777777778	48.42122222222222	49.32177777777778
PseEIIP-NCP-LDA	48.41711111111111	48.56677777777778	48.267222222222216	48.56677777777778	48.388333333333335	-3.1742222222222223	48.41688888888889	49.294333333333334
TNC-NCP-LDA	48.41711111111111	48.56677777777778	48.267222222222216	48.56677777777778	48.388333333333335	-3.1742222222222223	48.41688888888889	49.294333333333334
binary-NCP-NB	48.409888888888894	54.901333333333326	41.905777777777786	54.901333333333326	47.92322222222222	-3.1735555555555557	48.403444444444446	49.303666666666665
PseEIIP-ANF-NB	48.40844444444444	40.370666666666665	56.44288888888889	40.370666666666665	41.854888888888894	-3.2075555555555555	48.406666666666666	49.449888888888886
TNC-ANF-NB	48.40844444444444	40.370666666666665	56.44288888888889	40.370666666666665	41.854888888888894	-3.2075555555555555	48.406666666666666	49.449888888888886
binary-psednc-NB	48.40211111111111	56.153999999999996	40.63766666666667	56.153999999999996	48.60966666666666	-3.2166666666667	48.39566666666667	49.31433333333334
EIIP-NCP-RF	48.400444444444446	44.24955555555555	52.55166666666666	44.24955555555555	46.123444444444445	-3.2188888888888894	48.400666666666666	49.309666666666665
DNC-NCP-GB	48.37966666666667	48.98411111111111	47.776555555555554	48.98411111111111	48.657222222222224	-3.2366666666667	48.38011111111111	49.321555555555555
binary-psekn c-NB	48.36522222222223	55.95466666666667	40.763222222222225	55.95466666666667	48.50177777777778	-3.3237777777778	48.358777777777775	49.306777777777775
DNC-NCP-AB	48.358444444444444	47.332	49.387	47.332	47.70677777777777	-3.3063333333333333	48.35944444444445	49.31522222222222
DNC-TNC-LR	48.343666666666664	71.4601111111111	25.22277777777778	71.4601111111111	56.56722222222223	-4.0306666666666	48.34155555555556	49.24633333333333
TNC-PseEIIP-LR	48.33211111111112	73.82788888888889	22.83233333333333	73.82788888888889	57.149888888888896	-2.9328888888889	48.330000000000005	49.254222222222225
TNC-NCP-DT	48.30644444444445	50.42088888888889	46.19444444444444	50.42088888888889	49.34177777777778	-3.3945555555555558	48.30766666666667	49.22933333333334

binary-TNC-LR	48.29333333333333	47.92944444444444	48.65566666666667	47.92944444444444	47.95622222222222	-3.42988888888888	48.29255555555555	49.245111111111115
binary-ANF-LR	48.285333333333334	47.54822222222222	49.02044444444444	47.54822222222222	47.70066666666666	-3.456888888888889	48.284444444444446	49.268
binary-CKSNAP-LR	48.26522222222222	47.94033333333333	48.58888888888889	47.94033333333333	47.94811111111111	-3.486	48.26444444444444	49.232777777777784
binary-DNC-LR	48.26233333333333	47.867333333333335	48.65566666666667	47.867333333333335	47.90977777777778	-3.491888888888889	48.26155555555555	49.23122222222222
binary-NAC-LR	48.26233333333333	47.92944444444444	48.593555555555554	47.92944444444444	47.94144444444444	-3.4921111111111114	48.26155555555555	49.23111111111111
binary-psednc-LR	48.26233333333333	47.92944444444444	48.593555555555554	47.92944444444444	47.94144444444444	-3.4921111111111114	48.26155555555555	49.23111111111111
binary-pseknc-LR	48.26233333333333	47.867333333333335	48.65566666666667	47.867333333333335	47.90977777777778	-3.491888888888889	48.26155555555555	49.23122222222222
binary-scpsednc-LR	48.26233333333333	47.867333333333335	48.65566666666667	47.867333333333335	47.90977777777778	-3.491888888888889	48.26155555555555	49.23122222222222
EIIP-NAC-NB	48.239888888888885	46.36044444444444	50.106888888888896	46.36044444444444	43.69388888888889	-3.873444444444445	48.233555555555554	49.246111111111105
binary-PseEIIP-LR	48.23122222222222	47.867333333333335	48.593555555555554	47.867333333333335	47.895111111111106	-3.554111111111111	48.230444444444444	49.217444444444446
binary-PseEIIP-NB	48.230444444444444	55.45077777777778	40.99744444444445	55.45077777777778	48.135111111111115	-3.5631111111111116	48.224000000000004	49.23111111111111
binary-TNC-NB	48.230444444444444	55.45077777777778	40.99744444444445	55.45077777777778	48.135111111111115	-3.5631111111111116	48.224000000000004	49.23111111111111
EIIP-PseEIIP-DT	48.22977777777778	47.69555555555556	48.766777777777776	47.69555555555556	47.92711111111111	-3.542666666666667	48.23111111111111	49.22611111111111
EIIP-CKSNAP-NB	48.21222222222222	47.30466666666667	49.107777777777784	47.30466666666667	44.60388888888889	-3.885222222222222	48.20611111111111	49.291555555555554
EIIP-CKSNAP-LR	48.211555555555556	48.187	48.236666666666665	48.187	48.09077777777778	-3.60933333333333337	48.211666666666666	49.647555555555556
psednc-NCP-NB	48.16766666666667	47.63066666666666	48.69166666666667	47.63066666666666	44.785333333333334	-4.04733333333333334	48.16111111111111	49.19255555555556
binary-DNC-NB	48.160111111111114	56.013333333333335	40.294222222222224	56.013333333333335	48.41711111111111	-3.7011111111111115	48.153666666666666	49.198

EIIP-binary-Bagging	48.153555555555556	36.37244444444444	59.94455555555555	36.37244444444444	41.23566666666667	-3.69088888888893	48.15822222222222	49.285888888888884
binary-NAC-NB	48.13055555555556	56.095	40.153555555555556	56.095	48.42655555555555	-3.76355555555555	48.12411111111111	49.174
binary-PseEIIP-LDA	48.11555555555556	49.76122222222222	46.46911111111111	49.76122222222222	48.92766666666667	-3.783333333333333	48.11522222222223	49.13066666666666
binary-TNC-LDA	48.11555555555556	49.76122222222222	46.46911111111111	49.76122222222222	48.92766666666667	-3.783333333333333	48.11522222222223	49.13066666666666
NAC-ANF-LDA	48.09988888888889	50.01455555555555	46.18655555555555	50.01455555555555	48.97622222222222	-3.807888888888889	48.10066666666667	49.28311111111111
EIIP-psednc-NB	48.089444444444446	46.20033333333333	49.96622222222223	46.20033333333333	43.519555555555556	-4.195888888888889	48.08311111111111	49.18955555555556
ENAC-binary-LDA	48.080444444444446	48.25177777777778	47.91066666666667	48.25177777777778	48.16733333333334	-3.846888888888889	48.08111111111111	49.18111111111111
EIIP-ENAC-KNN	48.074111111111115	13.688555555555556	82.4858888888889	13.688555555555556	20.566222222222223	-5.293555555555556	48.08733333333333	49.31633333333333
NCP-NAC-NB	48.05555555555556	47.60633333333333	48.491777777777784	47.60633333333333	44.64533333333333	-4.280666666666666	48.04900000000001	49.147555555555556
CKSNAP-ANF-LR	48.05322222222222	43.647333333333336	52.46677777777778	43.647333333333336	45.79988888888889	-3.835	48.05711111111111	49.452111111111115
ENAC-DNC-KNN	48.046888888888894	13.385222222222222	82.73466666666667	13.385222222222222	20.207	-5.328777777777777	48.06011111111111	49.40744444444445
ENAC-scpsednc-KNN	48.046888888888894	13.385222222222222	82.73466666666667	13.385222222222222	20.207	-5.328777777777777	48.06011111111111	49.40744444444445
ENAC-NAC-KNN	48.031666666666666	13.385222222222222	82.70433333333334	13.385222222222222	20.200888888888887	-5.365333333333333	48.04488888888889	49.400555555555556
ENAC-psednc-KNN	48.031666666666666	13.385222222222222	82.70433333333334	13.385222222222222	20.200888888888887	-5.365333333333333	48.04488888888889	49.400555555555556
ENAC-psekn c-KNN	48.031666666666666	13.385222222222222	82.70433333333334	13.385222222222222	20.200888888888887	-5.365333333333333	48.04488888888889	49.400555555555556
EIIP-DNC-NB	48.02744444444445	46.07622222222222	49.96622222222223	46.07622222222222	43.43611111111111	-4.321999999999999	48.02111111111111	49.15855555555555

binary-TNC-RF	48.026222222222222	45.116888888888894	50.934777777777778	45.116888888888894	46.507	-3.945444444444445	48.025888888888886	49.205999999999996
ENAC-PseEIIP-KNN	48.018777777777778	13.385222222222222	82.678777777777778	13.385222222222222	20.195555555555554	-5.395555555555555	48.032000000000004	49.394666666666666
binary-DNC-LDA	48.013222222222225	49.89277777777777	46.13411111111111	49.89277777777777	48.92788888888889	-3.981222222222227	48.013666666666666	49.10055555555555
EIIP-PseEIIP-AB	48.00566666666666	47.41877777777778	48.59066666666667	47.41877777777778	47.696333333333335	-3.994555555555554	48.004666666666665	49.132
EIIP-TNC-AB	48.00566666666666	47.41877777777778	48.59066666666667	47.41877777777778	47.696333333333335	-3.994555555555554	48.004666666666665	49.132
binary-TNC-ET	47.99177777777778	43.46933333333333	52.52066666666666	43.46933333333333	45.455666666666666	-4.044222222222215	47.995111111111115	49.181666666666665
binary-NCP-LR	47.98555555555556	47.82322222222222	48.14588888888889	47.82322222222222	47.74955555555555	-4.039333333333333	47.98455555555555	49.159444444444446
PseEIIP-NCP-NB	47.98222222222223	47.22977777777778	48.72177777777778	47.22977777777778	44.38955555555555	-4.449111111111112	47.97566666666667	49.11655555555555
TNC-NCP-NB	47.98222222222223	47.22977777777778	48.72177777777778	47.22977777777778	44.38955555555555	-4.449111111111112	47.97566666666667	49.11655555555555
EIIP-binary-AB	47.977333333333334	45.217	50.733555555555554	45.217	46.42333333333333	-4.073111111111111	47.97522222222222	49.155
EIIP-psednc-ET	47.96277777777778	45.12533333333333	50.80033333333333	45.12533333333333	46.458333333333336	-4.077555555555556	47.96288888888889	49.129
psekn c-NCP-NB	47.959666666666664	47.26166666666667	48.644555555555556	47.26166666666667	44.367555555555555	-4.545111111111111	47.953111111111106	49.12444444444444
NCP-NAC-ET	47.92988888888889	44.05444444444444	51.80811111111112	44.05444444444444	45.686	-4.183	47.931333333333335	49.054111111111105
ENAC-CKSNAP-KNN	47.910444444444444	13.324555555555555	82.52288888888889	13.324555555555555	20.101333333333333	-5.681222222222222	47.92366666666666	49.38177777777778
EIIP-binary-RF	47.910222222222224	42.669444444444444	53.14944444444444	42.669444444444444	44.79655555555556	-4.237777777777778	47.90933333333333	49.15122222222222
binary-TNC-GB	47.90277777777778	46.258111111111106	49.54866666666666	46.258111111111106	46.99977777777778	-4.201888888888889	47.903444444444446	49.09155555555556
DNC-NCP-NB	47.87411111111111	47.38411111111111	48.351111111111116	47.38411111111111	44.45977777777778	-4.658444444444444	47.867555555555555	49.07055555555556

EIIP-NCP-LDA	47.86655555555555	48.397111111111116	47.33666666666667	48.397111111111116	48.129888888888885	-4.270222222222222	47.866888888888894	49.010555555555555
ENAC-ANF-LR	47.83633333333333	47.82300000000001	47.852222222222224	47.82300000000001	47.78944444444445	-4.333222222222222	47.83744444444445	49.14955555555556
DNC-NAC-NB	47.83311111111111	37.751555555555555	57.915	37.751555555555555	41.504555555555555	-4.434888888888889	47.833333333333336	49.45155555555556
EIIP-PseEIIP-LDA	47.79788888888889	48.572	47.02488888888889	48.572	48.12511111111111	-4.40933333333335	47.79866666666666	49.166000000000004
EIIP-TNC-LDA	47.79788888888889	48.572	47.02488888888889	48.572	48.12511111111111	-4.40933333333335	47.79866666666666	49.166000000000004
binary-PseEIIP-Bagging	47.77911111111111	34.50277777777777	61.06666666666667	34.50277777777777	39.75022222222222	-4.601666666666667	47.784666666666666	49.090111111111106
PseEIIP-NCP-AB	47.775666666666666	47.41588888888889	48.14033333333333	47.41588888888889	47.51711111111111	-4.457111111111111	47.77811111111111	48.99755555555556
TNC-NCP-AB	47.775666666666666	47.41588888888889	48.14033333333333	47.41588888888889	47.51711111111111	-4.457111111111111	47.77811111111111	48.99755555555556
PseEIIP-NCP-RF	47.74122222222222	45.657555555555554	49.827666666666666	45.657555555555554	46.549	-4.523777777777777	47.742444444444445	49.336
binary-PseEIIP-GB	47.739333333333335	46.416666666666664	49.06066666666666	46.416666666666664	46.977333333333334	-4.534777777777778	47.73866666666667	49.034666666666666
psednc-NCP-ET	47.732777777777784	46.08011111111111	49.382777777777775	46.08011111111111	46.83166666666667	-4.534777777777778	47.73133333333333	49.09188888888889
DNC-ANF-KNN	47.70922222222222	11.947333333333333	83.49933333333334	11.947333333333333	18.036555555555555	-6.642	47.72333333333333	49.169000000000004
binary-ANF-SVM	47.702777777777776	46.38155555555555	49.02655555555556	46.38155555555555	46.746111111111105	-4.657222222222222	47.70388888888888	48.973555555555556
DNC-NCP-ET	47.67388888888889	44.16888888888889	51.18344444444445	44.16888888888889	45.75933333333333	-4.658555555555555	47.676	48.97588888888889
scpsednc-ANF-KNN	47.662444444444446	11.854000000000001	83.49933333333334	11.854000000000001	17.912777777777777	-6.770666666666667	47.67655555555555	49.14866666666667
binary-DNC-RF	47.638444444444445	43.388999999999996	51.891444444444446	43.388999999999996	45.336222222222226	-4.710777777777775	47.64022222222222	49.001666666666665

EIIP-binary-SVM	47.620222222222225	46.15211111111111	49.090444444444444	46.15211111111111	46.56622222222222	-4.815888888888889	47.62122222222222	48.91877777777778
EIIP-ANF-KNN	47.61211111111111	11.838000000000001	83.41533333333332	11.838000000000001	17.81288888888889	-6.833888888888889	47.626666666666665	49.15133333333333
binary-CKSNAP-SVM	47.60644444444445	46.17777777777778	49.037444444444446	46.17777777777778	46.57333333333333	-4.844111111111111	47.60744444444444	48.912777777777784
binary-DNC-SVM	47.60644444444445	46.17777777777778	49.037444444444446	46.17777777777778	46.57333333333333	-4.844111111111111	47.60744444444444	48.912777777777784
binary-NAC-SVM	47.60644444444445	46.17777777777778	49.037444444444446	46.17777777777778	46.57333333333333	-4.844111111111111	47.60744444444444	48.912777777777784
binary-PseEIIP-SVM	47.60644444444445	46.17777777777778	49.037444444444446	46.17777777777778	46.57333333333333	-4.844111111111111	47.60744444444444	48.912777777777784
binary-TNC-SVM	47.60644444444445	46.17777777777778	49.037444444444446	46.17777777777778	46.57333333333333	-4.844111111111111	47.60744444444444	48.912777777777784
binary-psednc-SVM	47.60644444444445	46.17777777777778	49.037444444444446	46.17777777777778	46.57333333333333	-4.844111111111111	47.60744444444444	48.912777777777784
binary-pseknc-SVM	47.60644444444445	46.17777777777778	49.037444444444446	46.17777777777778	46.57333333333333	-4.844111111111111	47.60744444444444	48.912777777777784
binary-scpsednc-SVM	47.60644444444445	46.17777777777778	49.037444444444446	46.17777777777778	46.57333333333333	-4.844111111111111	47.60744444444444	48.912777777777784
PseEIIP-ANF-KNN	47.59466666666666	11.814222222222222	83.40344444444445	11.814222222222222	17.840555555555554	-6.958222222222222	47.608777777777775	49.129444444444445
psednc-ANF-KNN	47.59466666666666	11.814222222222222	83.40344444444445	11.814222222222222	17.840555555555554	-6.958222222222222	47.608777777777775	49.129444444444445
pseknc-ANF-KNN	47.59466666666666	11.814222222222222	83.40344444444445	11.814222222222222	17.840555555555554	-6.958222222222222	47.608777777777775	49.129444444444445
NAC-ANF-KNN	47.57655555555555	11.818888888888889	83.36233333333332	11.818888888888889	17.842111111111112	-6.993222222222222	47.590555555555554	49.122
TNC-NCP-LR	47.56333333333333	46.84177777777778	48.284666666666666	46.84177777777778	46.96888888888889	-4.898555555555556	47.563	48.940444444444445
psednc-NCP-LR	47.56333333333333	46.84177777777778	48.284666666666666	46.84177777777778	46.96888888888889	-4.898555555555556	47.563	48.940444444444445

PseEIIP-NCP-LR	47.547666666666665	46.903888888888886	48.19133333333333	46.903888888888886	46.99166666666667	-4.930000000000001	47.547333333333334	48.93088888888889
CKSNAP-ANF-KNN	47.54277777777778	11.770222222222223	83.34344444444444	11.770222222222223	17.75433333333333	-6.976444444444445	47.556555555555555	49.193
DNC-NCP-LR	47.53888888888889	46.767222222222216	48.31033333333333	46.767222222222216	46.91833333333333	-4.947555555555556	47.53866666666667	48.93022222222223
TNC-ANF-KNN	47.535222222222224	11.725555555555555	83.37322222222222	11.725555555555555	17.720555555555556	-7.122555555555556	47.54933333333334	49.105
ENAC-NAC-LDA	47.52744444444445	48.57988888888889	46.476111111111116	48.57988888888889	48.08455555555556	-4.957111111111111	47.52777777777778	49.071555555555555
DNC-NCP-Bagging	47.522333333333336	33.54255555555555	61.516444444444446	33.54255555555555	38.98288888888889	-5.101333333333333	47.529444444444444	49.068444444444445
DNC-ANF-LR	47.51222222222222	43.19566666666667	51.83533333333333	43.19566666666667	45.297	-4.923333333333333	47.51555555555555	49.18
PseEIIP-ANF-LR	47.510999999999996	43.132777777777775	51.89588888888889	43.132777777777775	45.266222222222225	-4.924555555555555	47.51433333333333	49.18255555555556
scpsednc-NCP-LR	47.507777777777775	46.70511111111111	48.31033333333333	46.70511111111111	46.87166666666667	-5.009555555555555	47.507555555555555	48.916000000000004
scpsednc-ANF-LR	47.49933333333334	43.14433333333333	51.86088888888889	43.14433333333333	45.26544444444445	-4.9481111111111105	47.50277777777777	49.175111111111114
psednc-DNC-ET	47.48844444444444	47.428	47.54711111111111	47.428	47.60033333333334	-5.036444444444445	47.48755555555556	50.251888888888885
EIIP-PseEIIP-NB	47.480777777777774	45.44388888888889	49.50555555555556	45.44388888888889	42.85377777777778	-5.513666666666665	47.474555555555554	48.96
EIIP-TNC-NB	47.480777777777774	45.44388888888889	49.50555555555556	45.44388888888889	42.85377777777778	-5.513666666666665	47.474555555555554	48.96
CKSNAP-NCP-LR	47.480222222222224	46.59366666666667	48.36611111111111	46.59366666666667	46.77455555555556	-5.073111111111111	47.479888888888894	48.904333333333334
TNC-ANF-LR	47.467777777777776	43.102333333333334	51.84	43.102333333333334	45.22566666666667	-5.012222222222222	47.471111111111114	49.16133333333333
psednc-ANF-LR	47.467777777777776	43.07211111111111	51.870222222222225	43.07211111111111	45.20844444444444	-5.0121111111111105	47.471111111111114	49.16133333333333

pseknc-ANF-LR	47.467777777777776	43.07211111111111	51.870222222222225	43.07211111111111	45.20844444444444	-5.0121111111111105	47.471111111111114	49.16133333333333
NCP-NAC-LR	47.461777777777776	46.767222222222216	48.156	46.767222222222216	46.882444444444445	-5.106222222222223	47.461555555555556	48.89522222222222
pseknc-NCP-LR	47.45511111111111	46.779666666666664	48.13033333333333	46.779666666666664	46.88633333333333	-5.119222222222223	47.45477777777778	48.891222222222225
EIIP-DNC-LDA	47.44766666666666	49.233222222222224	45.66266666666667	49.233222222222224	48.29877777777778	-5.123888888888889	47.44788888888889	48.916666666666664
EIIP-NCP-LR	47.431000000000004	46.861000000000004	48.00066666666666	46.861000000000004	46.913333333333334	-5.168111111111112	47.43077777777778	48.879222222222225
EIIP-ANF-LR	47.41911111111111	43.21677777777778	51.628	43.21677777777778	45.263555555555556	-5.103	47.422222222222224	49.16488888888889
TNC-NCP-Bagging	47.38444444444444	34.449222222222225	60.328222222222216	34.449222222222225	39.51788888888889	-5.435666666666666	47.388666666666666	48.90477777777778
ENAC-DNC-LDA	47.36611111111111	49.001444444444445	45.733222222222224	49.001444444444445	48.217777777777776	-5.279222222222222	47.367222222222225	48.94555555555556
TNC-NCP-ET	47.364888888888885	42.672444444444444	52.06166666666667	42.672444444444444	44.70933333333333	-5.311333333333334	47.367	48.93633333333334
PseEIIP-NCP-Bagging	47.33111111111111	34.903222222222226	59.769111111111116	34.903222222222226	39.843555555555554	-5.494222222222223	47.336222222222226	48.86955555555556
EIIP-DNC-RF	47.257444444444445	45.218777777777774	49.29044444444444	45.218777777777774	45.94788888888889	-5.534222222222223	47.254666666666665	48.80488888888889
NAC-ANF-LR	47.239666666666665	42.730444444444444	51.75566666666666	42.730444444444444	44.88877777777778	-5.475444444444444	47.24311111111111	49.04322222222223
ENAC-binary-SVM	47.22888888888889	47.166777777777774	47.29322222222223	47.166777777777774	47.045	-5.583888888888889	47.229888888888894	48.75688888888889
ENAC-binary-LR	47.160555555555554	46.43033333333333	47.89055555555555	46.43033333333333	46.64211111111111	-5.696888888888889	47.160333333333334	48.736444444444444
EIIP-PseEIIP-Bagging	47.144777777777776	35.83088888888889	58.464999999999996	35.83088888888889	40.37811111111111	-5.843111111111111	47.147999999999996	48.76833333333334
ENAC-CKSNAP-LR	47.087555555555554	47.40855555555556	46.76822222222222	47.40855555555556	47.242	-5.828	47.08833333333334	48.794000000000004

TNC-PseEIIP-NB	47.07877777777778	38.51555555555555	55.633	38.51555555555555	40.87122222222222	-6.050888888888888	47.074222222222225	48.94488888888889
PseEIIP-NAC-NB	47.019222222222226	38.132111111111115	55.897777777777776	38.132111111111115	40.724111111111114	-6.133222222222222	47.015	48.96311111111111
TNC-NAC-NB	47.019222222222226	38.132111111111115	55.897777777777776	38.132111111111115	40.724111111111114	-6.133222222222222	47.015	48.96311111111111
EIIP-NCP-ET	46.992333333333335	44.51611111111111	49.471444444444444	44.51611111111111	45.63911111111111	-6.024777777777778	46.99377777777778	48.70655555555555
ENAC-PseEIIP-LR	46.92044444444444	47.38533333333333	46.457	47.38533333333333	47.149	-6.163	46.92122222222222	48.72288888888889
ENAC-TNC-LR	46.92044444444444	47.38533333333333	46.457	47.38533333333333	47.149	-6.163	46.92122222222222	48.72288888888889
ENAC-psednc-LR	46.92044444444444	47.38533333333333	46.457	47.38533333333333	47.149	-6.163	46.92122222222222	48.72288888888889
ENAC-pseknc-LR	46.92044444444444	47.38533333333333	46.457	47.38533333333333	47.149	-6.163	46.92122222222222	48.72288888888889
ENAC-scpsednc-LR	46.92044444444444	47.35977777777778	46.48255555555556	47.35977777777778	47.13522222222222	-6.163111111111111	46.92122222222222	48.723
EIIP-ENAC-LDA	46.915333333333336	48.36566666666667	45.467444444444446	48.36566666666667	47.68477777777778	-6.177222222222222	46.916444444444444	48.68688888888889
binary-NCP-SVM	46.91444444444445	45.94	47.88966666666667	45.94	46.06777777777778	-6.272666666666667	46.91488888888889	48.61411111111111
ENAC-DNC-LR	46.90533333333334	47.35511111111111	46.457	47.35511111111111	47.12588888888889	-6.193222222222222	46.906111111111116	48.717
ENAC-NAC-LR	46.90533333333334	47.35511111111111	46.457	47.35511111111111	47.12588888888889	-6.193222222222222	46.906111111111116	48.717
ENAC-ANF-LDA	46.82866666666666	46.901333333333326	46.754555555555555	46.901333333333326	46.867777777777775	-6.354555555555556	46.828111111111106	48.68211111111111
psednc-ANF-SVM	46.81044444444444	44.619	49.00333333333333	44.619	45.61077777777778	-6.385111111111112	46.81122222222222	48.67566666666667
pseknc-ANF-SVM	46.80688888888889	44.51888888888888	49.09688888888889	44.51888888888888	45.55066666666667	-6.391111111111111	46.80788888888889	48.669555555555554
scpsednc-ANF-SVM	46.79877777777778	44.46522222222222	49.13411111111111	44.46522222222222	45.515	-6.409222222222223	46.79977777777778	48.678777777777775

NCP-ANF-SVM	46.79633333333334	45.574222222222225	48.02022222222222	45.574222222222225	45.87188888888889	-6.4932222222222222	46.797222222222224	48.58444444444444
EIIP-TNC-Bagging	46.708666666666666	34.276444444444444	59.149333333333324	34.276444444444444	39.133111111111106	-6.760111111111112	46.71288888888889	48.59411111111111
PseEIIP-ANF-SVM	46.70066666666666	44.23822222222222	49.16577777777778	44.23822222222222	45.33211111111112	-6.6083333333333333	46.702	48.60322222222222
EIIP-NAC-LDA	46.65311111111111	47.717666666666666	45.589333333333336	47.717666666666666	47.17633333333333	-6.7042222222222225	46.65344444444444	48.596333333333334
TNC-ANF-SVM	46.65122222222222	44.29777777777778	49.00666666666667	44.29777777777778	45.346111111111114	-6.7046666666666666	46.65244444444445	48.608333333333334
EIIP-NCP-SVM	46.63422222222223	45.679111111111105	47.59111111111111	45.679111111111105	45.86577777777777	-6.8068888888888885	46.635000000000005	48.492444444444445
DNC-ANF-SVM	46.63022222222222	44.462	48.80033333333333	44.462	45.42922222222222	-6.7482222222222222	46.63122222222222	48.60411111111111
DNC-NCP-SVM	46.583333333333336	45.38166666666667	47.78688888888889	45.38166666666667	45.68644444444445	-6.9043333333333333	46.58422222222222	48.48211111111111
NCP-NAC-SVM	46.583333333333336	45.38166666666667	47.78688888888889	45.38166666666667	45.68644444444445	-6.9043333333333333	46.58422222222222	48.48211111111111
PseEIIP-NCP-SVM	46.583333333333336	45.38166666666667	47.78688888888889	45.38166666666667	45.68644444444445	-6.9043333333333333	46.58422222222222	48.48211111111111
TNC-NCP-SVM	46.583333333333336	45.38166666666667	47.78688888888889	45.38166666666667	45.68644444444445	-6.9043333333333333	46.58422222222222	48.48211111111111
psednc-NCP-SVM	46.583333333333336	45.38166666666667	47.78688888888889	45.38166666666667	45.68644444444445	-6.9043333333333333	46.58422222222222	48.48211111111111
pseknc-NCP-SVM	46.583333333333336	45.38166666666667	47.78688888888889	45.38166666666667	45.68644444444445	-6.9043333333333333	46.58422222222222	48.48211111111111
scpsednc-NCP-SVM	46.583333333333336	45.38166666666667	47.78688888888889	45.38166666666667	45.68644444444445	-6.9043333333333333	46.58422222222222	48.48211111111111
ENAC-NCP-LR	46.54988888888889	46.13766666666667	46.96166666666667	46.13766666666667	46.18022222222223	-6.9368888888888889	46.54955555555556	48.464
NAC-ANF-SVM	46.489444444444445	44.321333333333335	48.659666666666666	44.321333333333335	45.28322222222222	-7.035111111111111	46.49044444444444	48.542

psednc-TNC-RF	46.440111111111115	46.54488888888889	46.33288888888889	46.54488888888889	46.51611111111111	-7.136222222222222	46.439	49.33888888888889
EIIP-ANF-SVM	46.367555555555555	44.19477777777777	48.54333333333333	44.19477777777777	45.165333333333336	-7.285111111111111	46.36911111111111	48.468777777777774
ENAC-NCP-SVM	46.31522222222222	46.245	46.387444444444445	46.245	46.140444444444445	-7.430000000000001	46.31622222222222	48.36833333333333
ENAC-PseEIIP-LDA	46.13355555555555	46.955999999999996	45.31066666666666	46.955999999999996	46.53666666666666	-7.738555555555554	46.13333333333333	48.40522222222222
ENAC-TNC-LDA	46.13355555555555	46.955999999999996	45.31066666666666	46.955999999999996	46.53666666666666	-7.738555555555554	46.13333333333333	48.40522222222222
DNC-pseknc-ET	46.10177777777778	44.464111111111116	47.74011111111111	44.464111111111116	45.28866666666667	-7.793777777777777	46.102222222222224	50.556333333333335
psednc-PseEIIP-RF	46.099000000000004	48.19122222222222	44.0	48.19122222222222	47.10655555555556	-7.872222222222222	46.09577777777778	49.653888888888886
TNC-NCP-GB	46.08255555555556	45.97711111111111	46.18922222222222	45.97711111111111	45.98566666666667	-7.853777777777777	46.08311111111111	48.25644444444444
binary-PseEIIP-ET	46.06177777777778	40.49311111111111	51.635777777777776	40.49311111111111	42.888666666666666	-7.915	46.06444444444444	48.324555555555555
PseEIIP-NCP-GB	46.02088888888889	45.98866666666667	46.053666666666665	45.98866666666667	45.96711111111111	-7.978777777777777	46.02133333333333	48.236444444444444
EIIP-PseEIIP-ET	45.69722222222222	41.79088888888889	49.60888888888889	41.79088888888889	43.46955555555556	-8.637333333333334	45.699888888888886	48.117666666666665
binary-PseEIIP-RF	45.64677777777777	42.852888888888884	48.44122222222222	42.852888888888884	44.04511111111111	-8.734555555555554	45.647333333333336	48.077111111111115
pseknc-PseEIIP-RF	45.56922222222223	43.50555555555556	47.63288888888889	43.50555555555556	44.411	-8.896666666666667	45.56944444444444	50.37800000000001
EIIP-PseEIIP-GB	45.51377777777778	45.56055555555556	45.46622222222223	45.56055555555556	45.52022222222222	-8.998777777777779	45.513333333333335	48.07211111111111
EIIP-TNC-ET	45.16622222222222	43.431777777777775	46.901777777777774	43.431777777777775	44.17855555555556	-9.680666666666667	45.16688888888889	47.934
TNC-NCP-RF	45.10455555555556	39.641888888888886	50.57222222222222	39.641888888888886	41.910444444444444	-9.856	45.10722222222223	47.961

psednc-scpsednc-KNN	45.02744444444445	15.818777777777777	74.25788888888889	15.818777777777777	22.30377777777778	-12.222777777777777	45.038222222222224	49.01744444444444
EIIP-TNC-GB	44.98255555555556	44.98744444444444	44.977333333333334	44.98744444444444	44.946	-10.061666666666666	44.98222222222222	47.856
scpsednc-pseknc-KNN	44.943555555555555	15.121999999999998	74.78711111111112	15.121999999999998	21.479444444444443	-12.549222222222221	44.95455555555556	48.87433333333334
scpsednc-PseEIIP-KNN	44.205000000000005	15.544222222222222	72.88777777777779	15.544222222222222	21.65533333333333	-14.288222222222222	44.215888888888884	48.589
DNC-NAC-SVM	44.07377777777778	34.38166666666667	53.77388888888889	34.38166666666667	37.977222222222224	-11.815	44.077666666666666	47.79311111111111
EIIP-scpsednc-LR	43.971222222222224	44.13	43.814	44.13	43.93088888888889	-12.100888888888889	43.972	47.53366666666667
DNC-NAC-LDA	43.891555555555556	47.01611111111111	40.763666666666666	47.01611111111111	45.568555555555555	-12.261444444444443	43.88988888888889	47.46655555555556
EIIP-pseknc-LR	43.891555555555556	44.21244444444444	43.57311111111111	44.21244444444444	43.941111111111105	-12.265555555555556	43.89266666666666	47.49644444444445
EIIP-NAC-LR	43.87611111111111	44.30755555555555	43.44711111111111	44.30755555555555	43.98744444444444	-12.297777777777778	43.87722222222222	47.47966666666667
TNC-pseknc-RF	43.85088888888889	42.486666666666665	45.21622222222223	42.486666666666665	42.97577777777778	-12.336	43.85155555555556	49.285333333333334
EIIP-PseEIIP-LR	43.84455555555556	44.150333333333336	43.541333333333334	44.150333333333336	43.88766666666666	-12.3588888888889	43.845666666666666	47.47833333333333
EIIP-psednc-LR	43.83177777777777	44.129444444444445	43.53655555555556	44.129444444444445	43.868222222222215	-12.3858888888889	43.83288888888889	47.47311111111111
EIIP-TNC-LR	43.70522222222222	44.093333333333334	43.31922222222222	44.093333333333334	43.79988888888889	-12.639111111111113	43.70633333333333	47.42211111111111
EIIP-DNC-LR	43.69522222222222	44.36288888888889	43.028555555555556	44.36288888888889	43.938	-12.660666666666666	43.69577777777778	47.41144444444444
EIIP-psednc-KNN	43.648111111111106	21.96211111111111	65.35644444444443	21.96211111111111	26.165555555555557	-15.086777777777778	43.65933333333333	47.93544444444444
EIIP-pseknc-KNN	43.460111111111104	21.868777777777776	65.07366666666667	21.868777777777776	26.03611111111111	-15.472555555555557	43.47133333333333	47.891444444444446

EIIP-PseEIIP-KNN	43.38066666666666	21.89	64.89366666666666	21.89	26.076444444444444	-15.575333333333333	43.391777777777776	47.87677777777778
EIIP-PseEIIP-RF	43.06944444444444	38.49022222222222	47.65288888888889	38.49022222222222	40.278777777777776	-13.941111111111111	43.071555555555555	47.184888888888885
EIIP-TNC-RF	43.05122222222222	39.818666666666665	46.28633333333333	39.818666666666665	41.12722222222222	-13.944444444444445	43.05255555555556	47.147111111111116
scpsednc-NAC-KNN	42.846666666666664	13.033	72.68144444444445	13.033	18.492	-17.835111111111111	42.85711111111111	48.45911111111111
EIIP-TNC-KNN	42.7	21.178444444444445	64.24166666666666	21.178444444444445	25.374777777777776	-17.067444444444444	42.71	47.67011111111111
TNC-NAC-SVM	42.39433333333333	31.59522222222222	53.199777777777776	31.59522222222222	35.37411111111111	-15.216888888888889	42.397444444444446	47.376555555555555
EIIP-scpsednc-SVM	42.26588888888889	45.31033333333333	39.21955555555556	45.31033333333333	43.93944444444444	-15.549888888888889	42.265	46.79522222222222
scpsednc-TNC-KNN	42.15155555555556	12.602333333333332	71.72122222222222	12.602333333333332	17.838555555555555	-19.418333333333333	42.16177777777778	48.458999999999996
DNC-scpsednc-KNN	42.14611111111111	12.405999999999999	71.90933333333334	12.405999999999999	17.543444444444443	-19.652888888888889	42.157555555555554	48.40233333333333
EIIP-DNC-KNN	41.99688888888889	20.217555555555556	63.797666666666665	20.217555555555556	24.174999999999997	-18.795888888888889	42.007666666666665	47.571333333333335
EIIP-scpsednc-KNN	41.92488888888889	19.68433333333333	64.18766666666666	19.68433333333333	23.38322222222222	-19.261888888888889	41.93588888888889	47.70044444444444
psednc-pseknc-KNN	41.88655555555555	13.087555555555555	70.70766666666667	13.087555555555555	18.59888888888889	-19.158777777777778	41.897666666666666	48.467999999999996
EIIP-psednc-SVM	41.721111111111114	44.70166666666667	38.73888888888889	44.70166666666667	43.37611111111111	-16.642555555555557	41.720333333333336	46.57722222222222
EIIP-pseknc-SVM	41.665222222222226	44.579	38.75	44.579	43.287444444444446	-16.753333333333334	41.66433333333333	46.56355555555555
EIIP-NAC-KNN	41.584666666666664	20.489666666666665	62.70088888888889	20.489666666666665	24.110666666666663	-19.689333333333334	41.59522222222222	47.519111111111116
EIIP-PseEIIP-SVM	41.55155555555555	44.54288888888889	38.559	44.54288888888889	43.21611111111111	-16.982555555555553	41.550777777777775	46.52166666666667

EIIP-TNC-SVM	41.54222222222222	44.71622222222223	38.36522222222223	44.71622222222223	43.31944444444444	-17.00644444444444	41.54088888888889	46.492555555555555
PseEIIP-NAC-LDA	41.20444444444445	42.32211111111111	40.08844444444444	42.32211111111111	41.827666666666666	-17.62333333333335	41.20511111111111	46.55222222222223
TNC-NAC-LDA	41.20444444444445	42.32211111111111	40.08844444444444	42.32211111111111	41.827666666666666	-17.62333333333335	41.20511111111111	46.55222222222223
EIIP-NAC-SVM	41.07688888888889	43.986666666666665	38.16455555555555	43.986666666666665	42.71644444444444	-17.92977777777776	41.07566666666666	46.348
EIIP-DNC-SVM	40.87766666666667	44.04611111111112	37.707	44.04611111111112	42.67055555555556	-18.33433333333333	40.876666666666665	46.276444444444444
DNC-PseEIIP-SVM	40.86322222222222	32.11522222222222	49.617444444444445	32.11522222222222	35.20066666666666	-18.45933333333333	40.86611111111111	46.95611111111111
TNC-PseEIIP-LDA	40.74755555555556	42.715111111111106	38.77744444444445	42.715111111111106	41.86288888888889	-18.5533333333333	40.74633333333333	46.44555555555556
psednc-PseEIIP-KNN	40.48755555555556	12.597333333333333	68.39911111111111	12.597333333333333	17.557555555555556	-22.636111111111111	40.49822222222222	47.93
psekn c-NAC-KNN	40.46077777777778	10.97	69.97244444444443	10.97	15.278111111111112	-23.92733333333333	40.471222222222224	48.065555555555555
psednc-NAC-KNN	40.26422222222223	11.20122222222222	69.34744444444443	11.20122222222222	15.450222222222221	-24.38266666666665	40.27433333333333	48.068333333333335
EIIP-CKSNAP-KNN	40.260666666666665	13.800888888888888	66.73966666666666	13.800888888888888	18.31111111111114	-23.46944444444445	40.27033333333333	47.76222222222222
pseknc-PseEIIP-KNN	40.248000000000005	12.056222222222223	68.45866666666667	12.056222222222223	16.87533333333333	-23.3168888888889	40.257333333333335	48.12455555555555
DNC-PseEIIP-LDA	39.148111111111106	39.11055555555556	39.18655555555555	39.11055555555556	39.07311111111111	-21.75888888888887	39.148666666666664	46.03988888888889
DNC-TNC-LDA	39.148111111111106	39.11055555555556	39.18655555555555	39.11055555555556	39.07311111111111	-21.75888888888887	39.148666666666664	46.03988888888889
TNC-pseknc-KNN	39.04922222222222	9.818222222222223	68.30211111111112	9.818222222222223	13.981333333333332	-26.43055555555557	39.06022222222222	47.989000000000004
psednc-DNC-KNN	38.68855555555555	9.04288888888889	68.35711111111112	9.04288888888889	12.776777777777777	-28.20977777777774	38.699888888888886	48.154555555555554

psednc-PseEIIP-ET	38.425444444444445	38.26177777777778	38.58711111111111	38.26177777777778	38.351888888888894	-23.2077777777778	38.42444444444445	46.92566666666667
PseEIIP-NAC-KNN	38.068333333333335	9.863666666666667	66.29155555555556	9.863666666666667	13.57	-29.16055555555554	38.077666666666666	47.82966666666667
psednc-TNC-ET	37.919333333333334	36.67011111111111	39.168	36.67011111111111	37.26822222222222	-24.186444444444444	37.91911111111111	46.83344444444444
DNC-NAC-KNN	37.897333333333336	8.700666666666667	67.11677777777778	8.700666666666667	12.292111111111112	-29.72511111111111	37.90888888888889	48.157000000000004
psednc-TNC-KNN	37.72311111111111	9.602555555555556	65.865	9.602555555555556	13.359	-29.522444444444446	37.733777777777775	47.598444444444446
TNC-NAC-KNN	37.51377777777778	8.931777777777777	66.116	8.931777777777777	12.543777777777779	-30.298222222222225	37.52377777777778	48.141666666666666
psekn c-PseEIIP-ET	37.282000000000004	35.351888888888894	39.214	35.351888888888894	36.111777777777775	-25.506777777777778	37.28288888888889	47.97322222222222
TNC-pseknc-ET	37.18244444444444	34.83433333333333	39.53377777777777	34.83433333333333	35.89666666666667	-25.717555555555556	37.18411111111111	47.840444444444444
DNC-PseEIIP-KNN	37.16777777777778	8.008000000000001	66.34855555555555	8.008000000000001	11.281777777777778	-31.587555555555557	37.17822222222222	48.10022222222222
DNC-TNC-KNN	36.379333333333335	8.8918888888889	63.885666666666665	8.8918888888889	12.252555555555555	-32.5892222222222	36.388999999999996	47.79211111111111
DNC-NAC-AB	36.11244444444444	34.754333333333335	37.465999999999994	34.754333333333335	35.12733333333333	-27.85811111111111	36.11033333333333	45.62855555555556
TNC-PseEIIP-KNN	35.77622222222222	8.392777777777777	63.18055555555556	8.392777777777777	11.607444444444445	-33.75611111111111	35.78644444444444	47.76933333333333
DNC-PseEIIP-AB	34.28366666666667	34.656555555555556	33.91188888888889	34.656555555555556	34.468777777777774	-31.566222222222223	34.28422222222222	45.08411111111111
DNC-TNC-AB	34.28366666666667	34.656555555555556	33.91188888888889	34.656555555555556	34.468777777777774	-31.566222222222223	34.28422222222222	45.08411111111111
TNC-PseEIIP-AB	33.13155555555555	31.91766666666667	34.34388888888889	31.91766666666667	32.16855555555556	-33.8593333333333	33.13077777777778	44.78988888888889
PseEIIP-NAC-AB	32.952111111111115	30.263444444444442	35.641	30.263444444444442	30.98177777777778	-34.28777777777784	32.95222222222222	44.911

TNC-NAC-AB	32.952111111111115	30.263444444444442	35.641	30.263444444444442	30.98177777777778	-34.28777777777784	32.95222222222222	44.911
TNC-PseEIIP-SVM	28.102666666666668	25.84888888888889	30.36266666666667	25.84888888888889	26.242444444444445	-44.16922222222224	28.105777777777778	44.68333333333333
DNC-NAC-GB	27.672333333333334	27.30311111111111	28.040555555555557	27.30311111111111	27.299777777777777	-44.81566666666665	27.671888888888887	44.390888888888895
DNC-PseEIIP-Bagging	26.60666666666667	23.69311111111111	29.517333333333333	23.69311111111111	24.38088888888889	-46.9973333333333	26.605444444444444	44.67466666666667
DNC-PseEIIP-GB	26.53377777777778	25.59155555555556	27.476333333333333	25.59155555555556	25.639666666666667	-47.15144444444444	26.53388888888889	44.425
DNC-TNC-GB	26.436777777777777	25.358888888888888	27.515555555555554	25.358888888888888	25.403222222222222	-47.3655555555556	26.43722222222222	44.449
PseEIIP-NAC-GB	26.420333333333335	26.038444444444444	26.799777777777777	26.038444444444444	25.974777777777778	-47.3292222222223	26.41911111111111	44.34177777777778
TNC-PseEIIP-GB	26.048111111111112	25.343222222222224	26.753555555555558	25.343222222222224	25.422222222222224	-48.0153333333334	26.048444444444446	44.159888888888894
TNC-NAC-Bagging	25.825111111111113	25.872111111111114	25.775333333333336	25.872111111111114	25.784888888888887	-48.46166666666666	25.823666666666668	44.22588888888889
DNC-TNC-Bagging	25.74711111111111	23.864333333333335	27.63222222222222	23.864333333333335	24.332555555555555	-48.59266666666666	25.748	44.67166666666667
PseEIIP-NAC-Bagging	25.715333333333334	23.558555555555557	27.871666666666666	23.558555555555557	24.096444444444444	-48.6967777777778	25.71522222222222	44.60155555555556
DNC-TNC-DT	25.57177777777778	18.930111111111113	32.214555555555556	18.930111111111113	20.149777777777775	-49.414	25.572444444444443	45.25011111111111
DNC-NAC-Bagging	25.461222222222222	25.016555555555556	25.90311111111111	25.016555555555556	25.274444444444445	-49.14799999999996	25.45988888888889	44.66744444444444
DNC-NAC-ET	25.25288888888889	25.179111111111112	25.325666666666663	25.179111111111112	25.209333333333333	-49.6672222222222	25.252222222222223	44.443222222222225
TNC-NAC-DT	25.150333333333336	17.18411111111111	33.11555555555556	17.18411111111111	18.35977777777778	-50.68111111111111	25.14977777777778	45.651444444444444
TNC-PseEIIP	25.03488888888889	23.578222222222223	26.49411111111111	23.578222222222223	23.912666666666667	-50.0162222222222	25.036	44.62466666666667

P-Bagging								
DNC-PseEIIP-ET	24.984555555555556	24.410666666666668	25.558333333333334	24.410666666666668	24.492444444444445	-50.160111111111114	24.984333333333336	44.623
DNC-NAC-RF	24.852888888888888	25.916555555555554	23.78688888888889	25.916555555555554	25.558222222222224	-50.55666666666665	24.851555555555553	44.342777777777776
DNC-PseEIIP-DT	24.85188888888889	16.937333333333335	32.76766666666666	16.937333333333335	18.34	-51.01866666666667	24.852444444444444	45.284444444444446
DNC-TNC-ET	24.755777777777777	24.743000000000002	24.766000000000002	24.743000000000002	24.752444444444443	-50.60577777777774	24.754444444444445	44.468333333333334
DNC-PseEIIP-RF	24.74488888888889	24.947555555555553	24.540777777777777	24.947555555555553	24.895666666666667	-50.619111111111111	24.744222222222223	44.470333333333336
TNC-PseEIIP-RF	24.64677777777778	24.651	24.639111111111113	24.651	24.563222222222223	-50.83844444444444	24.645111111111113	44.36655555555555
TNC-NAC-ET	24.63011111111111	23.413444444444444	25.845333333333333	23.413444444444444	23.66722222222222	-50.86955555555556	24.629222222222225	44.67044444444444
TNC-PseEIIP-ET	24.588444444444445	23.373333333333335	25.803	23.373333333333335	23.629555555555555	-50.91488888888896	24.58811111111111	44.55188888888889
PseEIIP-NAC-DT	24.526888888888887	17.011333333333333	32.041111111111114	17.011333333333333	18.094	-51.85377777777777	24.52622222222222	45.621111111111105
TNC-PseEIIP-DT	24.50711111111111	17.90111111111111	31.111666666666665	17.90111111111111	18.903555555555556	-51.70488888888889	24.506555555555554	45.449777777777776
PseEIIP-NAC-ET	24.294777777777778	23.387444444444444	25.197222222222223	23.387444444444444	23.47888888888889	-51.73699999999995	24.292333333333332	44.525555555555556
DNC-TNC-RF	24.159222222222223	25.179777777777776	23.134777777777778	25.179777777777776	24.64777777777778	-51.92366666666666	24.157333333333334	44.416111111111114
TNC-NAC-RF	23.959111111111113	25.175444444444445	22.739444444444445	25.175444444444445	24.864555555555555	-52.14622222222223	23.957444444444445	44.015
CKSNAP-PseEIIP-SVM	80.471	78.13977777777778	82.80533333333334	78.13977777777778	80.00888888888889	61.016111111111111	80.47244444444443	75.34855555555556
CKSNAP-scpsednc-SVM	79.524	77.495	81.5551111111111	77.495	79.08111111111111	59.10733333333334	79.525	74.23266666666667
CKSNAP-PseEIIP-LDA	73.851	72.077	75.6258888888889	72.077	73.51066666666667	47.78666666666666	73.85144444444444	68.79422222222223

CKSNAP-TNC-LDA	73.851	72.077	75.6258888888889	72.077	73.51066666666667	47.78666666666666	73.85144444444444	68.79422222222223
psednc-scpsednc-SVM	72.024	69.19522222222221	74.85477777777777	69.19522222222221	71.27744444444446	44.16866666666667	72.02477777777777	66.81966666666666
scpsednc-psebnc-GB	69.332	68.77744444444444	69.88444444444445	68.77744444444444	69.297	38.758	69.331	64.26833333333333
psednc-scpsednc-RF	65.247	64.44755555555555	66.04444444444444	64.44755555555555	65.03277777777778	30.55333333333335	65.24588888888889	60.78977777777777
ENAC-scpsednc-AB	60.803	60.38388888888889	61.21822222222223	60.38388888888889	60.77977777777778	21.68933333333334	60.80111111111111	57.04822222222222
scpsednc-ANF-RF	59.953	58.03355555555556	61.869888888888894	58.03355555555556	59.21711111111111	19.97199999999998	59.95166666666666	56.60755555555556
CKSNAP-TNC-ET	59.283	57.21577777777778	61.35288888888889	57.21577777777778	58.538333333333334	18.62188888888886	59.28422222222222	56.74488888888889
psednc-NAC-DT	59.061	62.43944444444445	55.68333333333333	62.43944444444445	60.355888888888884	18.20422222222222	59.06144444444445	55.638000000000005
EIIP-CKSNAP-DT	58.458	57.37800000000001	59.54277777777778	57.37800000000001	57.83044444444445	17.00433333333335	58.46044444444445	55.224666666666664
psednc-ANF-AB	58.391	57.675000000000004	59.10633333333333	57.675000000000004	58.227777777777774	16.81822222222222	58.39066666666666	55.592
psednc-pseknc-NB	57.397	42.40566666666667	72.39822222222222	42.40566666666667	49.62833333333334	15.58066666666666	57.401888888888884	55.24011111111111
CKSNAP-TNC-DT	56.833	55.88066666666666	57.78888888888889	55.88066666666666	56.25366666666667	13.70366666666667	56.83488888888889	54.29988888888889
EIIP-pseknc-AB	56.633	53.57033333333333	59.69755555555556	53.57033333333333	55.135777777777776	13.29077777777778	56.634	54.96077777777778
binary-pseknc-DT	56.436	56.93133333333334	55.940555555555555	56.93133333333334	56.65855555555556	12.87844444444444	56.43611111111111	54.01433333333333
pseknc-ANF-DT	56.052	56.452333333333335	55.654777777777774	56.452333333333335	56.12188888888889	12.18	56.053666666666665	53.73722222222222
ENAC-ANF-Bagging	53.651	39.94311111111111	67.3678888888889	39.94311111111111	46.107	7.650111111111111	53.65566666666667	52.07977777777778

psednc-NAC-ET	53.475	55.59177777777778	51.355	55.59177777777778	54.61622222222222	6.985555555555554	53.47333333333333	52.82011111111112
ENAC-ANF-ET	51.961	46.50111111111111	57.42688888888889	46.50111111111111	49.12433333333334	3.96411111111111	51.96377777777778	51.20911111111111
NCP-ANF-DT	51.289	48.32922222222223	54.25366666666667	48.32922222222223	49.735	2.58877777777776	51.291444444444444	50.75533333333333
psekn c-NCP-LDA	50.895	51.132333333333335	50.659666666666666	51.132333333333335	50.96622222222222	1.788111111111112	50.89588888888889	50.565555555555555
ENAC-DNC-AB	50.741	50.42666666666666	51.053444444444445	50.42666666666666	50.50622222222222	1.47944444444443	50.74011111111111	50.493
ENAC-NAC-AB	50.741	50.42666666666666	51.053444444444445	50.42666666666666	50.50622222222222	1.47944444444443	50.74011111111111	50.493
ENAC-PseEIIP-DT	50.509	51.50677777777778	49.513999999999996	51.50677777777778	50.97377777777778	1.02155555555556	50.510333333333335	50.37722222222222
ENAC-binary-DT	50.506	52.12422222222222	48.885555555555555	52.12422222222222	51.18277777777777	0.98888888888889	50.504888888888885	50.34411111111111
PseEIIP-ANF-Bagging	50.223	37.54377777777778	62.91799999999999	37.54377777777778	42.965666666666664	0.59522222222221	50.230777777777774	50.29677777777778
binary-PseEIIP-AB	49.693	49.86511111111111	49.51844444444444	49.86511111111111	49.53722222222222	-0.58055555555555	49.69188888888888	50.01166666666667
binary-TNC-AB	49.693	49.86511111111111	49.51844444444444	49.86511111111111	49.53722222222222	-0.58055555555555	49.69188888888888	50.01166666666667
EIIP-ENAC-RF	49.643	44.407555555555554	54.887	44.407555555555554	46.381	-0.820777777777778	49.64722222222222	49.98066666666667
psekn c-ANF-LDA	49.628	49.45366666666667	49.804111111111105	49.45366666666667	49.471444444444444	-0.72922222222219	49.629	50.13966666666667
binary-NCP-RF	49.482	46.19266666666667	52.77511111111111	46.19266666666667	47.57233333333333	-1.04999999999998	49.48388888888889	49.904333333333334
EIIP-binary-GB	49.324	50.06966666666667	48.57511111111111	50.06966666666667	49.58122222222222	-1.342	49.32222222222222	49.83422222222222
binary-TNC-KNN	49.323	20.756	77.92222222222222	20.756	28.28277777777778	-1.57566666666668	49.33922222222222	49.967999999999996
PseEIIP-ANF-AB	48.954	47.49333333333333	50.41766666666667	47.49333333333333	48.01422222222222	-2.11977777777778	48.955333333333336	49.64377777777778

TNC-ANF-AB	48.954	47.49333333333333	50.41766666666667	47.49333333333333	48.01422222222222	-2.1197777777777778	48.955333333333336	49.64377777777778
pseknc-NCP-ET	48.869	44.474999999999994	53.270111111111106	44.474999999999994	46.37544444444445	-2.2952222222222223	48.87244444444445	49.58588888888889
EIIP-binary-LR	48.244	47.867333333333335	48.61922222222222	47.867333333333335	47.901333333333326	-3.5285555555555557	48.24322222222222	49.22344444444444
EIIP-NCP-NB	48.146	47.02433333333333	49.255	47.02433333333333	44.21955555555556	-4.1266666666666667	48.13944444444444	49.18355555555556
ENAC-TNC-KNN	48.034	13.385222222222222	82.70911111111111	13.385222222222222	20.201666666666668	-5.358999999999999	48.047222222222224	49.40155555555556
ENAC-ANF-KNN	47.949	12.473888888888888	83.45244444444444	12.473888888888888	19.165333333333333	-5.646111111111111	47.963	49.21355555555556
EIIP-TNC-DT	47.703	46.42111111111112	48.986444444444444	46.42111111111112	47.00622222222222	-4.595888888888889	47.70388888888889	48.973222222222226
EIIP-DNC-GB	47.512	47.105555555555554	47.91777777777778	47.105555555555554	47.27777777777778	-4.980333333333333	47.51166666666667	48.91811111111111
NCP-ANF-LR	47.504	46.50355555555555	48.50366666666667	46.50355555555555	46.69566666666667	-5.039111111111111	47.50355555555555	48.900444444444446
PseEIIP-ANF-LDA	47.423	46.82844444444444	48.02022222222222	46.82844444444444	47.09166666666667	-5.158444444444445	47.42444444444445	48.830444444444446
TNC-ANF-LDA	47.423	46.82844444444444	48.02022222222222	46.82844444444444	47.09166666666667	-5.158444444444445	47.42444444444445	48.830444444444446
DNC-PseEIIP-NB	47.053	38.91388888888889	55.184333333333335	38.91388888888889	41.37288888888889	-6.066666666666666	47.04900000000001	49.058
DNC-TNC-NB	47.053	38.91388888888889	55.184333333333335	38.91388888888889	41.37288888888889	-6.066666666666666	47.04900000000001	49.058
EIIP-DNC-ET	46.928	42.24311111111111	51.61833333333333	42.24311111111111	44.23522222222222	-6.186222222222223	46.93055555555556	48.628
DNC-ANF-LDA	46.897	48.48222222222223	45.31166666666667	48.48222222222223	47.63122222222222	-6.215222222222222	46.897	48.63166666666667
EIIP-ENAC-LR	46.882	47.14533333333333	46.62	47.14533333333333	47.001333333333335	-6.239222222222222	46.882777777777775	48.71933333333333
EIIP-NAC-ET	46.878	42.67566666666667	51.083666666666666	42.67566666666667	44.45766666666666	-6.287444444444444	46.87966666666667	48.68366666666667
PseEIIP-NCP-ET	46.835	43.208	50.46344444444444	43.208	44.66477777777778	-6.385555555555555	46.83588888888889	48.67044444444444

CKSNAP-NCP-SVM	46.566	45.44477777777777	47.689	45.44477777777777	45.70355555555555	-6.943222222222222	46.56688888888888	48.47633333333333
DNC-pseknc-KNN	38.567	8.583	68.57255555555555	8.583	12.277999999999999	-28.437333333333335	38.577777777777776	48.07377777777778
DNC-TNC-SVM	35.393	30.160222222222224	40.63044444444444	30.160222222222224	31.856555555555556	-29.491111111111113	35.39522222222222	45.516888888888886
TNC-NAC-GB	26.191	25.84411111111111	26.535333333333334	25.84411111111111	25.78677777777778	-47.772333333333336	26.189777777777778	44.30244444444444
DNC-NAC-DT	25.883	18.74211111111111	33.02511111111111	18.74211111111111	20.153888888888886	-48.784	25.883555555555557	45.57666666666667
PseEIIP-NAC-RF	25.064	26.325777777777777	23.80022222222222	26.325777777777777	25.86088888888889	-50.07277777777777	25.063222222222223	44.38466666666667

Table S2 entails about the parameters utilized in 13 different sequence encoding methods. Gap represents the value with which different kmers are generated by skipping different nucleotides. PropertyName represents selection of properties for computing the correlation among different nucleotides.

Encoder	Range of hyperparameters	Op. Parameters
**CKSNAP**	Gap=[1-15]	Gap=10
**SCPSEDNC**	Gap=[1-15], PropertyName	Gap=10, PropertyName=['Base stacking', 'Stability']
**Binary**	**-**	**-**
**PseDNC**	Gap=[1-15], PropertyName	Gap=10,PropertyName= ['Base stacking', 'Stability'],
**PseKNC**	Gap==[1-15], PropertyName, kmer=[1-5]	Gap=10, PropertyName= ['Rise', 'Roll'], kmer=2
**DNC**	-	-
**TNC**	-	-
**PseEIIP**	**-**	**-**

**NAC**	**-**	**-**
**ANF**	**-**	**-**
**EIIP**	**-**	**-**
**ENAC**	Window_size	Window_size = 10
**NCP**	**-**	-
